# Unusual Inner-Salt Guaiazulene Alkaloids and *bis*-Sesquiterpene from the South China Sea Gorgonian *Muriceides collaris*

**DOI:** 10.1038/s41598-017-08100-z

**Published:** 2017-08-09

**Authors:** Pinglin Li, Xiaoling Liu, Hongyan Zhu, Xuli Tang, Xuefeng Shi, Yonghong Liu, Guoqiang Li

**Affiliations:** 1 Key Laboratory of Marine Drugs, Chinese Ministry of Education, School of Medicine and Pharmacy, Ocean University of China, Qingdao, 266003 China; 2 Laboratory of Marine Drugs and Biological Products, National Laboratory for Marine Science and Technology, Qingdao, 266235 P. R. China; 3 College of Chemistry and Chemical Engineering, Ocean University of China, Qingdao, 266100 China; 4 CAS Key Laboratory of Tropical Marine Bio-resources and Ecology, Guang dong Key Laboratory of Marine Materia Medica, Research Center for Marine Microbes, South China Sea Institute of Oceanology, Chinese Academy of Sciences, Guangzhou, 510301 China

## Abstract

Three new guaiazulene alkaloids muriceidines A–C (**1**–**3**) and one new *bis*-sesquiterpene muriceidone A (**4**), were isolated from the South China Sea gorgonian *Muriceides collari*s. Muriceidines are the first examples structurally architected by guaiazulene coupling with an inner-salt *Δ* ^1^-pipecolic acid *via* a unique sp^2^ methine-bridged linkage, and the *bis*-sesquiterpene was comprised by a guaiazulene and a indene units linked through a unprecedented carbon-carbon *σ*-bond between the high steric bridgehead carbon C-10 of guaiazulene moiety and C-2′ of indene moiety. The chiral compounds **2**–**4** were obtained initially as racemates and further separated by chiral HPLC methods. The inner-salt structures of **1**–**3** and absolute configurations of **2**–**4** were fully elucidated by calculated ^13^C NMR, ECD and OR with quantum chemical calculation methods. Compound **1** showed cytotoxicity against K562 cell lines with IC_50_ value of 8.4 *μ*M and antifouling activity against the larvae of the barnacle *Balanus albicostatus* with EC_50_ value of 11.9 *μ*g/mL and potent therapeutic index (LC_50_/EC_50_ = 3.66). Also the racemic (±)-**3** showed cytotoxicities against both HL-60 and K562 cell lines with IC_50_ values of 2.2 and 3.7 *μ*M, respectively. A semisynthetic trial was performed to validate the proposed biosynthetic hypotheses.

## Introduction

Azulene is one of the most important non-benzenoid aromatic compounds^1, 2^. Especially the azulenes containing nitrogen, mainly including aza-azulenes, N-heterocyclic fused azulene, and other azulene derivatives coupled with nitrogen units, possess special physico-chemical properties and significant pharmacological and therapeutic actions^3–5^. Although many terrestrial plants and marine corals are the rich resources of natural azulene derivatives with the biogenic fixed guaiazulene (GA) skeleton^6–10^, their structural variety almost limits to different exocyclic oxygenate patterns or only a few dimmers having the space comfort linkage of C-3–C-3′ or C-2–C-3′. In addition, almost all the azulenes containing nitrogen were obtained from chemical synthesis. However, gorgonian showed some specificity in GA derivation. In 1984, the first GA alkaloid, N,N-dimethylamino-3-guaiazulenyl methane, was isolated from an unidentified blue gorgonian^11^. A recent study of *Anthogorgia* species showed an interesting coexistence of GA and indene derivatives^10^.

**Figure 1:**
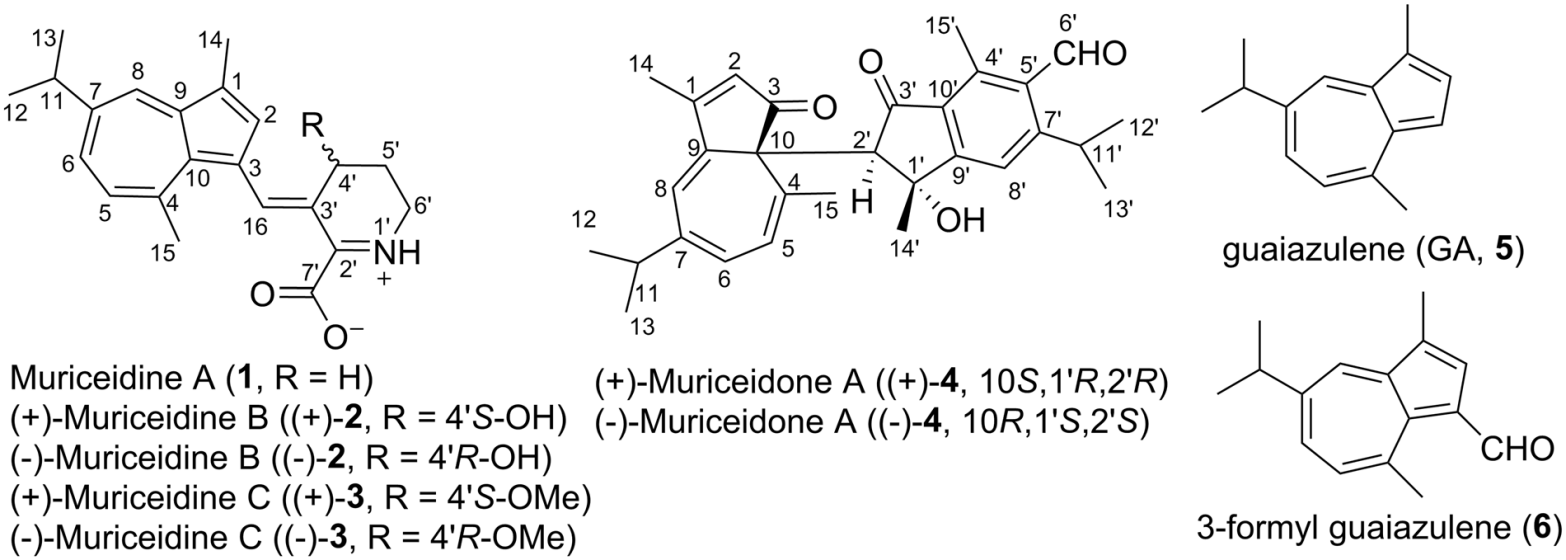
Structures of **1**–**6** from Gorgonian *Muriceides collaris*.

Recently we encountered a rare gorgonian *Muriceides collaris* shaping in fan with unique azure branches, which is distributed only in few regions of South China Sea. To date, apart from our previous preliminary work on *M. collaris*, resulting in the isolation of cholesterol, batylalcohol, uracil, thymine, (2′-deoxyuridine, 2′-deoxythymidine, and thymidine^12^. There were few reports on the chemical profiles of genus *Muriceides* (family Plexauridae) containing more than 20 species. In present study, bioassay-guided isolation of *M. collaris* yielded three new guaiazulene-type alkaloids and one new *bis*-sesquiterpene (Fig. 1), named as muriceidines A–C (**1**–**3**) and muriceidone A (**4**), together with the known GA (**5**) and 3-formyl guaiazulene (**6**). The muriceidines showed a novel structure architected by guaiazulene coupling with an inner-salt *Δ* ^1^-pipecolic acid *via* a unique sp^2^ methine-bridged linkage, and the unprecedented *bis*-sesquiterpene muriceidone A was characterized by a high steric linkage between azulene and indene units through a carbon-carbon *σ*-bond. All the chiral members **2**–**4** were initially obtained as racemates and were successfully separated by chiral HPLC methods. Apart from using the extensive spectroscopic analyses including IR, MS and NMR, especially the inner-salt structures in **1**–**3** and absolute configurations of **2**–**4** were fully elucidated by calculated ^13^C NMR, ECD and OR with quantum chemical calculation methods using time-dependent density functional theory (TDDFT). Additionally, cytotoxic and antifouling activities were assayed for all the new compounds **1**–**6**.

## Results and Discussion

### Structure Elucidation

**Figure 2:**
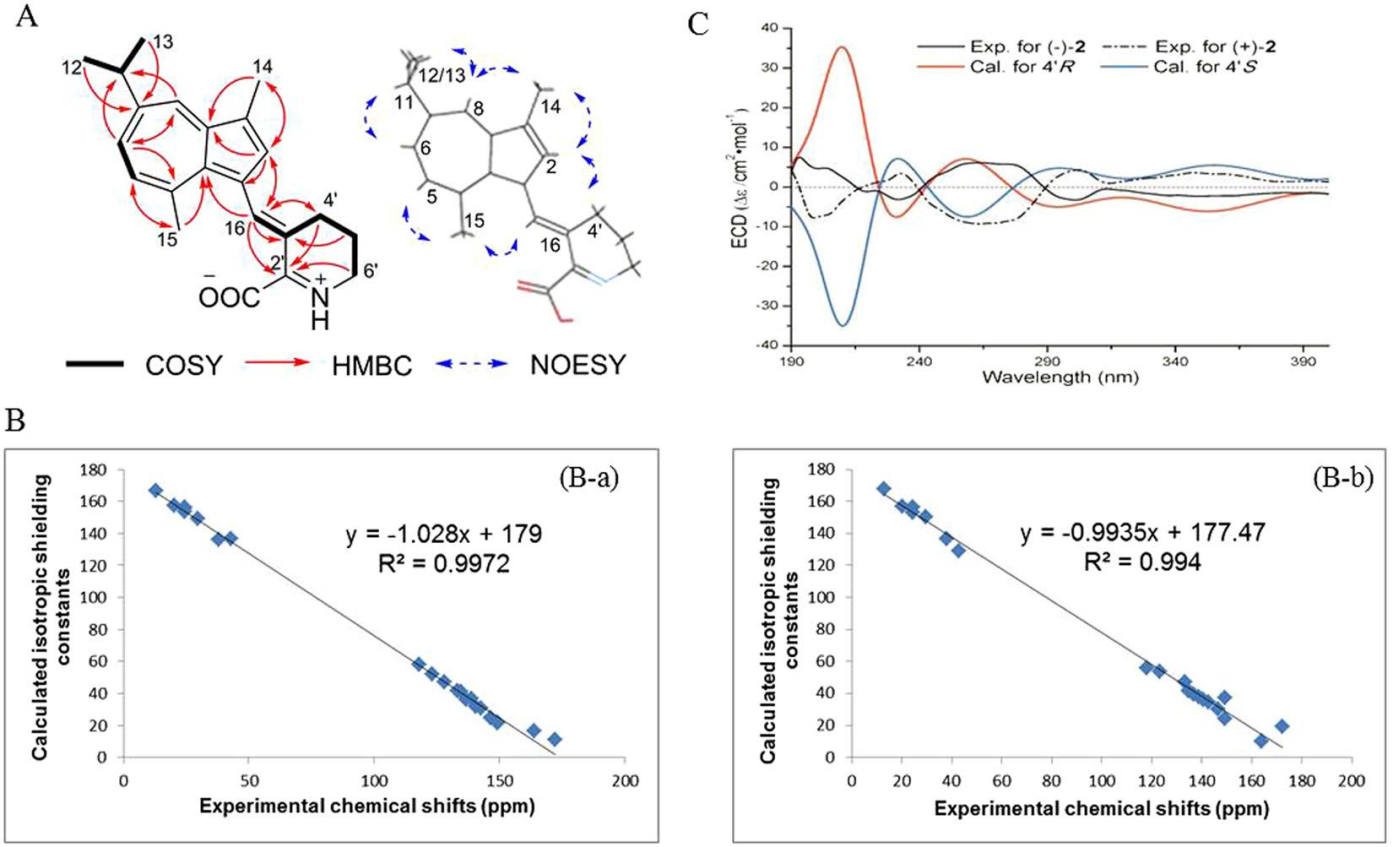
Structure elucidation of muriceidines A-C (**1**–**3**). (**A**) Key COSY, HMBC and NOESY correlations of **1**. (**B**) Correlation of experimental chemical shifts and calculated isotropic shielding constants of compound **1** with the respective inner-salt (B-a) and non-ionized (B-b) structure. (**C**) Calculated and experimental ECD curves of compounds (+)-**2** and (−)-**2**.

Muriceidine A (**1**) was obtained as dark red amorphous power. Its molecular formula was determined as C_22_H_25_NO_2_ by HRESIMS (*m*/*z* 336.1959 [M + H]^+^, calcd 336.1958; 358.1776 [M + Na]^+^, calcd 358.1778), indicating eleven degrees of unsaturation. The IR absorption bands indicated the presence of carbonyl (1701 cm^−1^) and imino (1649 cm^−1^) groups. In the ^1^H NMR spectrum of **1** (Table 1), the characteristic proton signals represented by an aromatic ABX coupling system at *δ*_H_ 8.17 (1H, d, *J* = 2.2 Hz, H-8), 7.51 (1H, dd, *J* = 11.0, 2.2 Hz, H-6), and 7.33 (1H, d, *J* = 11.0 Hz, H-5), one aromatic singlet proton signal at *δ*_H_ 7.81 (1H, s, H-2), two olefinic methyl proton signals at *δ*_H_ 2.58 (3H, s, H_3_-14) and 3.17 (3H, s, H_3_-15), as well as one isopropyl proton signals at *δ*_H_ 3.11 (1H, septet, *J* = 6.6, 6.6 Hz, H-11) and 1.37 (6H, d, *J* = 6.6 Hz, H_3_-12/13), strongly suggested the presence of a C-3 substituted GA moiety in **1** by comparing its NMR data with those of GA derivatives^13^. The key HMBC correlations from H-12 and H-13 to C-7 (*δ*_C_ 146.8) and C-11 (*δ*_C_ 38.2), from H-6 and H-8 to C-11, from H-14 to C-1 (*δ*_C_ 128.2), C-2 (*δ*_C_ 139.0) and C-9 (*δ*_C_ 142.8), and from H-15 to C-4 (*δ*_C_ 149.3), C-5 (*δ*_C_ 133.4) and C-10 (*δ*_C_ 141.0) further confirmed this speculation (Fig. 2A).

**Table 1:** NMR Data for muriceidines A–C (**1**–**3**) (*δ* in ppm).

no.	**1**		**2**		**3**	
	*δ*_C_^a^	*δ*_H_^*b*^ (mult *J* in Hz)	*δ*_C_^c^	*δ*_H_^*d*^ (mult *J* in Hz)	*δ*_C_^a^	*δ*_H_^*b*^ (mult *J* in Hz)
1	128.2, C		131.5, C		128.7, C	
2	139.0, CH	7.81 (s)	141.7, CH	8.51 (s)	140.0, CH	7.88 (s)
3	123.5, C		124.2, C		123.0, C	
4	149.3, C		149.9, C		149.3, C	
5	133.4, CH	7.33 (d, 11.0)	136.3, CH	7.58 (d, 10.4)	133.8, CH	7.36 (d, 10.4)
6	137.1, CH	7.51 (dd, 11.0, 2.2)	138.8, CH	7.75 (d, 10.4)	137.2, CH	7.54 (d, 10.4)
7	146.8, C		150.9, C		147.3, C	
8	134.8, CH	8.17 (d, 2.2)	136.2, CH	8.35 (br s)	134.8, CH	8.18 (s)
9	142.8, C		146.5, C		143.8, C	
10	141.0, C		143.9, C		141.5, C	
11	38.2, CH	3.11 (dq, 6.6, 6.6)	39.3, CH	3.21 (dq, 6.6, 6.6)	38.2, CH	3.12 (dq, 6.6, 6.6)
12	24.5, CH_3_	1.37 (d, 6.6)	24.6, CH_3_	1.40 (d, 6.6)	24.6, CH_3_	1.37 (d, 6.6)
13	24.5, CH_3_	1.37 (d, 6.6)	24.6, CH_3_	1.40 (d, 6.6)	24.6, CH_3_	1.37 (d, 6.6)
14	13.3, CH_3_	2.58 (s)	13.2, CH_3_	2.62 (s)	13.4, CH_3_	2.59 (s)
15	29.7, CH_3_	3.17 (s)	29.8, CH_3_	3.13 (s)	29.8, CH_3_	3.18 (s)
16	149.4, CH	9.56 (s)	151.4, CH	9.16 (s)	152.8, CH	9.90 (s)
2′	172.3, C		173.1, C		170.5, C	
3′	118.0, C		117.9, C		117.5, C	
4′	24.7, CH_2_	2.95 (t, 6.0)	61.6, CH	5.05 (s)	69.7, CH	4.69 (s)
5′	20.4, CH_2_	2.03 (m)	29.8, CH_2_	2.16 (br d, 13.8), 1.95 (t, 13.8)	23.5, CH_2_	2.43 (br d, 14.3), 1.82 (m)
6′	42.9, CH_2_	3.77 (t, 5.5)	38.5, CH_2_	3.81 (m), 3.63 (dd, 15.0, 4.8)	38.2, CH_2_	3.82 (br d, 7.7)
7′	163.9, C		167.2, C		162.9, C	
OMe					54.3, CH_3_	3.45 (s)

^a^Recorded at 150 MHz in CDCl_3_.

^b^Recorded at 600 MHz in CDCl_3_.

^c^Recorded at 150 MHz in CD_3_OD.

^d^Recorded at 600 MHz in CD_3_OD.

Another key fragment of -CH_2_-CH_2_-CH_2_- in **1** was readily recognized from COSY correlations between H_2_-4′/H_2_-5′ and H_2_-5′/H_2_-6′. Thus, the HMBC correlations from H_2_-6′ [*δ*_H_ 3.77 (2H, t, *J* = 5.5 Hz)] to C-2′ (*δ*_C_ 172.3), and from H_2_-4′ [*δ*_H_ 2.95 (2H, t, *J* = 6.0 Hz)] to C-2′, combined with the HRESIMS data could establish the *Δ* ^1^-pipecolic acid moiety in **1** (Fig. 2A). This elucidation was further confirmed by comparing the NMR data with those of furpipate derivatives formed from furfural or 5-hydroxy-methylfurfural in the presence of Lysine and those of anthosamines^14, 15^. The sequential connection between GA moiety and *Δ* ^1^-pipecolic acid was achieved by HMBC correlations from the methine-bridged proton of H-16 [*δ*_H_ 9.56 (1H, s, H-16)] to C-2′, C-3′ (*δ*_C_ 118.0), C-4′ (*δ*_C_ 24.7), C-2, C-3 (*δ*_C_ 123.5) and C-10, and from H_2_-4′ to C-16 (*δ*_C_ 149.4) to form the planar structure of **1** (Figs 2A and SS10 in Supporting Information).

The geometry of the double bond ∆^3′^ ^16^ in **1** was assigned as *E* evident from NOESY correlations of H-2/H_2_-4′ and H-16/H_3_-15 but lack of correlation of H-16/H_2_-4′ (Figs 2A and SS11 in Supporting Information), which was supported by a molecular model analysis (the spatial distance of H-2/H_2_-4′ is <2.5 Å in *E* geometry but >4.7 Å in *Z* geometry) on the basis of conformational search in the Spartan 10 package showing that **1** fits in a seemingly planar structure with the C-2–C-3–C-16–C-3′ dihedral angel of 22^°^ (Fig. S2 and Table S2 in Supporting Information). However, there was still a pending issue whether compound **1** could be inner salt or non-ionized structure. Therefore, the quantum-mechanical GIAO calculations for the ^13^C NMR chemical shifts of **1** were performed using the DFT theory method at the RB3LYP/6-311+g(2d,p) level^16^. The calculated isotropic shielding constants of inner salt structure of **1** were in good correlation with the experimental ^13^C NMR chemical shifts (Table 2). After linear regression which gives a reasonable *R* ^2^ of 0.9972 and slope of −1.028 (Fig. 2B)^16^, the mean absolute error with respect to the experimental data was 2.16 ppm for **1**. Whereas, the calculation data of non-ionized structure of **1** gave an unacceptable linear relation along with a low *R* ^2^ value of 0.9941 and a high mean absolute error of 3.09 ppm (Fig. 2B)^16^. It was noticeable that the calculated chemical shifts for C-2′ and C-7′ in the two cases showed distinct difference. The resonance of C-2′ in inner salt structure is more deshielded relative to C-7′, whereas it will be opposite in non-ionized structure of **1** (Tables 2 and S5 in Supporting Information). Thus, the structure of **1** was fully elucidated.

**Table 2:** The calculated ^13^C NMR chemical shifts of compound **1**.

no.	*δ*_exp_	*δ*_calc_	*δ*_scalc_	*δ*_mcalc_	*δ*_mscalc_	no.	*δ*_exp_	*δ*_calc_	*δ*_scalc_	*δ*_mcalc_	*δ*_mscalc_
1	128.2	135.0	128.5	132.0	127.3	12	24.5	25.9	22.4	25.9	22.0
2	139.0	145.1	138.3	144.5	139.7	13	24.5	25.8	22.3	25.9	21.9
3	123.5	130.1	123.7	128.8	124.2	14	13.3	15.2	11.9	14.8	10.9
4	149.3	160.7	153.4	158.5	153.7	15	29.7	32.7	29.0	32.1	28.0
5	133.4	140.4	133.7	135.3	130.6	16	149.4	160.1	152.9	145.2	140.5
6	137.1	145.5	138.7	143.1	138.4	2′	172.3	170.9	163.4	163.1	158.2
7	146.8	157.2	150.0	152.1	147.3	3′	118.0	123.8	117.6	126.8	122.2
8	134.8	140.7	134.0	140.9	136.2	4′	24.7	28.5	24.9	29.5	25.5
9	142.8	151.3	144.3	147.8	143.1	5′	20.4	24.4	20.9	25.5	21.6
10	141.0	150.3	143.3	146.2	141.4	6′	42.9	45.2	41.1	53.4	49.3
11	38.2	46.0	41.9	46.0	41.9	7′	163.9	165.4	158.1	172.3	167.4

***δ***_**calc**_: unscaled chemical shifts of inner salt structure relative to TMS at the same level of theory.

***δ***_**scalc**_: calculated chemical shifts of inner salt structure after linear scaling.

***δ***_**mcalc**_: unscaled chemical shifts of non-ionized structure relative to TMS at the same level of theory.

**Figure 3:**
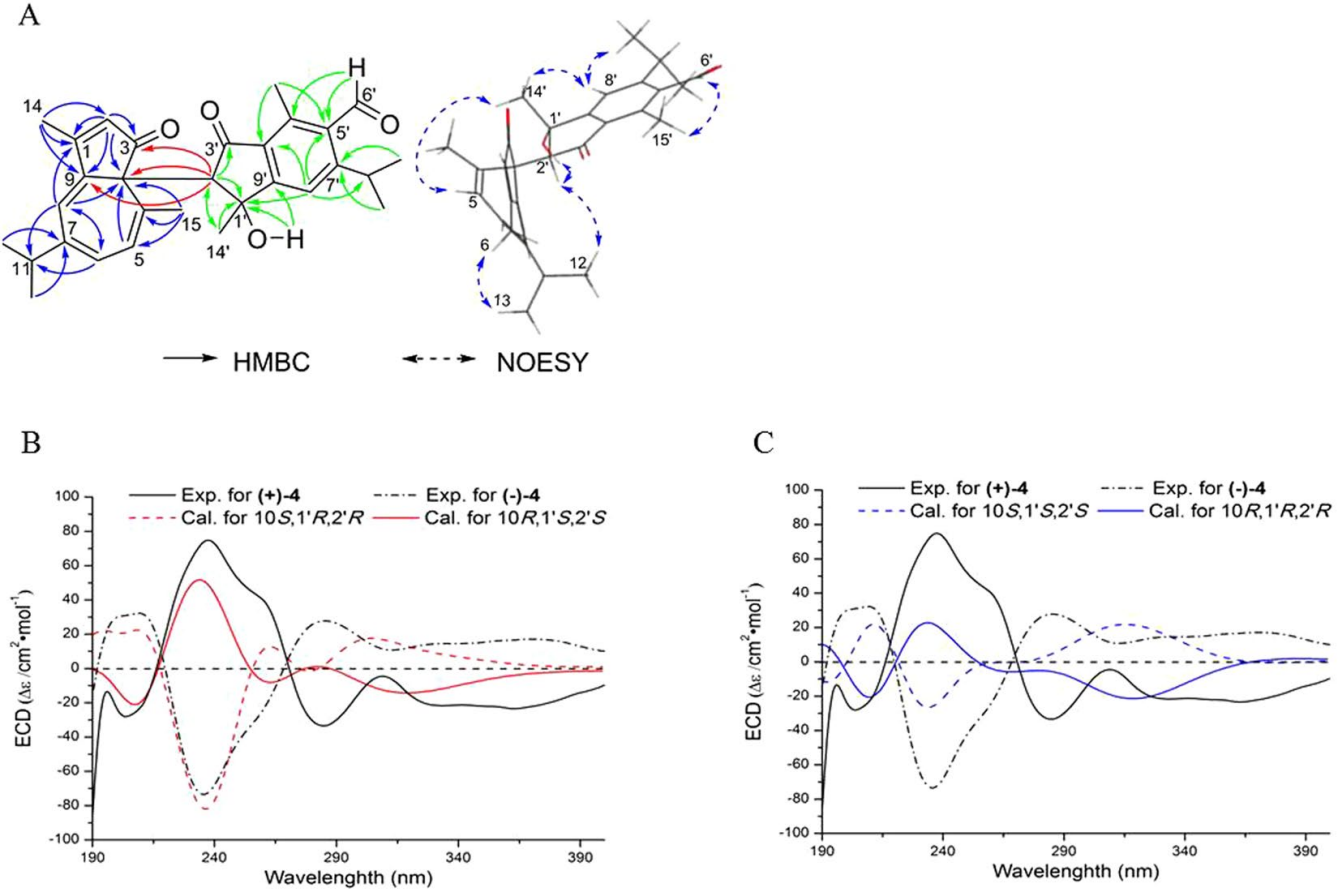
Structure elucidation of muriceidone A (**4**). (**A**) Key HMBC and NOESY correlations of **4**. (**B**) Calculated and experimental ECD curves of compounds (+)-**4** and (−)-**4** in 10*S*,1′*R*,2′*R*/10*R*,1′*S*,2′*S* configuration. (**C**) Calculated and experimental ECD curves of compounds (+)-**4** and (−)-**4** in 10*S*,1′*S*,2′*S*/10*R*,1′*R*,2′*R* configuration.

***δ***_**mscalc**_: calculated chemical shifts of non-ionized structure after linear scaling.

(±)-Muriceidine B (**2**) had molecular formula of C_22_H_25_NO_3_ from its HRESIMS. The UV, IR, and 1D NMR spectra of compound **2** were very similar to those of **1** (Table 1 and Supporting Information), except for an oxygenated methine signal (*δ*_H_ 5.05, s, H-4′; *δ*_C_ 61.6, d, C-4′) in **2** instead of one methylene signal in **1**, indicating that **2** was a hydroxylated product of **1**. The position of the hydroxyl group in **2** was assigned at C-4′ deduced from COSY correlations of H-4′/H_2_-5′ and H_2_-5′/H_2_-6′, as well as HMBC correlations from H-4′ to C-2′ (*δ*_C_ 173.1), C-3′ (*δ*_C_ 117.9), C-6′ (*δ*_C_ 38.5) and C-16 (*δ*_C_ 151.4). NOESY correlations of H_3_-15/H-16 and H-2/H-4′ suggested a *Z* geometry for the double bond ∆^3′^ ^16^. The initial speculation of racemic mixture for **2** was caused by the failed observation of optical rotation value and ECD spectrum. Further chiral separation was undertaken on chiral HPLC to yield (+)- and (−)-**2** (Fig. S1 in Supporting Information). (+)-**2** and (−)-**2** gave almost opposite optical rotation values and exhibited mirror-like ECD curves (Fig. 2C). To determine their absolute configurations, the stable conformers of respective (+)- and (−)-**2** were studied theoretically by TDDFT/ECD calculations at RB3LYP/DGDZVP level (Supporting Information)^17^. The experimental ECD spectrum of (+)-**2** exhibited three moderate positive Cotton effects (CEs) at 232.7, 302.0 and 347.3 nm and two strong negative CEs at 199.1 and 263.3 nm, which matched well with the calculated ECD data for 4′*S* configuration (Fig. 2C). On the contrary, calculated ECD of 4′*R* configuration exhibited mirror-like CEs consistent with the experimental data of (−)-**2**. Thus, 4′*S* and 4′*R* were finally assigned for (+)-**2** and (−)-**2**, respectively.

(±)-Muriceidine C (**3**) was also obtained as a racemic mixture in initial isolation. Appearance of an extra methoxyl signal (*δ*_H_ 3.45, s, 3H; *δ*_C_ 54.3, q) in **3** comparing to **2** indicated that **3** was a methylated derivative of **2**, which was supported by HMBC correlations from OMe to C-4′ (*δ*_C_ 69.7). After chiral separation, the experimental optical rotation values and ECD spectra of (+)-and (−)-**3** were consistent with those of respective (+)- and (−)-**2** (Fig. 2C), suggesting that they share the same configurations.

(±)-Muriceidone A (**4**) was obtained as yellow amorphous power. Its HRESIMS (*m*/*z* 481.2360 [M+Na]^+^ (calcd 481.2349)) provided molecular formula as C_30_H_34_O_4_, requiring fourteen degrees of unsaturation. The IR absorption bands indicated the presence of carbonyl (1701, 1697, 1695 cm^−1^), phenyl (1556, 1457 cm^−1^), and hydroxyl (3621 cm^−1^) groups. In accordance with the molecular formula, 30 carbon signals in its ^13^C NMR spectrum were distinguished as eight methyls, nine methines (five olefinic), thirteen quaternary carbons (one oxygenated, eight olefinic, and three carbonyl carbons) by DEPT and HMQC spectra (Table 3 and Figs SS39 and SS40 in Supporting Information). ^1^H NMR spectrum of compound **4** showed the presence of two isopropyl groups [(*δ*_H_ 2.69 (1H, dq, *J* = 6.6, 7.2 Hz), 3.59 (1H, dq, *J* = 6.6, 6.6 Hz), 1.30 (3H, d, *J* = 6.6 Hz), 1.27 (3H, d, *J* = 6.6 Hz), 1.17 (3H, d, *J* = 7.2 Hz), 1.13 (3H, d, *J* = 6.6 Hz)], four olefinic methyl groups [*δ*_H_ 2.69 (3H, s), 2.31 (3H, s), 1.87 (3H, s), 1.83 (3H, s)], and an aromatic AB system [*δ*_H_ 6.54 (1H, d, *J* = 6.6 Hz) and 6.26 (1H, d, *J* = 6.6 Hz)]. The aforementioned spectral information strongly suggested that compound **4** could be a *bis*-sesquiterpene with derivative GA or indene unit^10^. HMBC correlations from H_3_-12/13 to C-7 and C-11, from H_3_-14 to C-1, C-2 and C-9, from H_3_-15 to C-4, C-5 and C-10, from H-2 to C-3 and C-10, from H-8 to C-9, C-10, and C-11, further confirmed the presence of 3-oxo-10-substituted dihydroguaiazulene moiety (Fig. 3A). The presence of 1′-hydroxyl-5′-aldehyde-3′-oxo-indene moiety in **4** was evident from the HMBC correlations from H_3_-12′/13′ to C-7′ and C-11′, from H_3_-14 to C-1′, C-2′ and C-9′, from H_3_-15′ to C-4′, C-5′ and C-10′, from H-8′ to C-1′, C-5′, C-10′ and C-11′, from H-6′ to C-5′, from H-2′ to C-3′, and from the hydroxyl proton (*δ*_H_ 2.17, br s) to C-2′ and C-9′ (Figs 3A and SS42 in Supporting Information). Finally, the key HMBC correlations from H-2′ to C-3, C-9 and C-10 could establish the planar structure of **4** as shown in Fig. 3A by a carbon-carbon σ-bond between C-10 and C-2′.

**Table 3:** ^1^H and ^13^C NMR data for muriceidone A (**4**) in CDCl_3_.

Position	*δ*_C_^*a*^	*δ*_H_^*b*^ (*J* in Hz)	Position	*δ*_C_^*a*^	*δ*_H_^*b*^ (*J* in Hz)
1	167.3, C		1′	76.7, C	
2	132.1, CH	6.19 (s)	2′	59.5, CH	3.03 (s)
3	206.4, C		3′	202.3, C	
4	133.4, C		4′	140.0, C	
5	124.3, CH	6.26 (d, 6.6)	5′	134.4, C	
6	126.3, CH	6.54 (d, 6.6)	6′	194.6, CH	10.61 (s)
7	147.5, C		7′	157.4, C	
8	117.1, CH	6.44 (s)	8′	118.7, CH	7.49 (s)
9	138.3, C		9′	162.4, C	
10	55.7, C		10′	129.3, C	
11	36.0, CH	2.69 (dq, 6.6, 7.2)	11′	29.6, CH	3.59 (dq, 6.6, 6.6)
12	23.0, CH_3_	1.13 (d, 6.6)	12′	23.9, CH_3_	1.27 (d, 6.6)
13	23.5, CH_3_	1.17 (d, 7.2)	13′	24.0, CH_3_	1.30 (d, 6.6)
14	15.1, CH_3_	2.31 (s)	14′	28.4, CH_3_	1.87 (s)
15	24.1, CH_3_	1.83 (s)	15′	14.3, CH_3_	2.69 (s)
			OH-3′		2.17 (br s)

^*a*^Recorded at 150 MHz. ^*b*^Recorded at 600 MHz.

In NOESY spectrum of **4**, the NOE correlation of hydroxyl proton with H_3_-14′ (Figs 3A and SS44 in Supporting Information) and lack of NOE correlation of H_3_-14′ with H-2′ indicated that H-2′ and the hydroxyl group was located on the same side of the indene plane. The initial optical rotation value near to be zero suggested its enantisomeric feature, and a successful chiral separation on HPLC for **4** yielded optical pure compounds (+)-**4** and (−)-**4**, showing opposite optical rotation values of 308.7 and −316.7, respectively. To determine the absolute configurations of **4**, the stable conformers of structural candidates of 10*R*,1′*S*,2′*S*, 10*S*,1′*R*,2′*R*, 10*R*,1′*R*,2′*R*, and 10*S*,1′*S*,2′*S* for **4** were studied theoretically by TDDFT/ECD calculations at RB_3_LYP/DGDZVP level^17^. It was evident that whether the main CE at about 230 nm could be positive or negative depends on the C-10 configuration, while the configurations of C-2′ and C-3′ were sensitive to the intensity of the CE (Figs 3B and S6–S9 Supporting Information). Accordingly, the calculated ECD for 10*S*,1′*R*,2′*R* configuration matched well with the experimental ECD spectrum of (+)-**4** with a strong negative CE at 235.4 nm and a relatively weak positive CE at 368.4 nm, which was opposite to the experimental ECD spectrum of (−)-**4** and calculated ECD for 10*R*,1′*S*,2′*S* configuration (Fig. 3B). The result was further supported by the computed optical rotation (OR) for the optimal B3LYP/6-31G(d,p)-optimized geometries of the four structural candidates (Supporting Information). The calculated OR values for **4a1** and **4b1** with respective 10*S*,2′*R*,3′*R* and 10*R*,2′*S*,3′*S* absolute configurations were correspondingly 276.4 and −278.5 at the B3LYP/6-311++G(2d,p) level^17^, which were close to the experimental data of 308.7 and −316.7 and differed greatly from the calculated 693.4 and −746.2 for the respective 10*S*,1′*S*,2′*S* and 10*R*,1′*R*,2′*R* candidates (**4c1** and **4d1**). It indicated that the C-10 configuration played a decisive role in the nature of OR, while C-1′ and C-2′ configurations together reflected their degrees. And this trend was still consistent in those OR of all the dominant conformers and in the combined ones after Boltzmann weighting (Table S18 in Supporting Information). Therefore, the absolute configurations of (+)-**4** and (−)-**4** were determined as 10*S*,2′*R*,3′*R* and 10*R*,2′*S*,3′*S*, respectively.

### Biological Activity

It was reported that GA derivatives generally possess antioxidant, antiallergic, and anti-inflammatory activities, and have wide application as cosmetic color additive and anti-ulcer drug^13, 18, 19^. However, the novel muriceidines showed significant cytotoxic and antifouling bioactivities. In cytotoxic assay against HeLa, K562, HL-60, and A549 human tumor cell lines using the MTT method (Table 4)^20^, the racemic (±)-**3** showed strong cytotoxicities against both HL-60 and K562 cell lines with IC_50_ values of 2.19 and 3.68 *μ*M, respectively, and compound **1** showed moderate cytotoxicity against K562 cell lines with IC_50_ value of 8.37 *μ*M, neither racemic (±)-**2** nor the optically pure enantiomers, (+)-**2** and (−)-**2**, as well as (±)-**4**, (+)-**4** and (−)-**4**, were active against the selected tumor cell lines. The optically pure enantiomer (+)-**3** was also inactive to the selected four tumor cell lines while (−)-**3** showed moderate cytotoxic activity with IC_50_ value of 5.08 *μ*M against HL-60 cell line. Furthermore, compound **1** also had antifouling activity against the larvae of the barnacle *Balanus albicostatus* with EC_50_ value of 11.9 *μ*g/mL with high therapeutic index (LC_50_/EC_50_ = 3.66), stronger than the positive control (Cu^2+^, EC_50_ and LC_50_ = 1.0 mg/mL, LC_50_/EC_50_ = 1.0)^21^. Interestingly, 3-formyl guaiazulene (**6**) showed a lower EC_50_ value of 2.39 *μ*g/mL but lower therapeutic ratio of LC_50_/EC_50_ = 2.60.

**Table 4:** Cytotoxic activities of compounds **1**–**4** (IC_50_ *μ*M) with adramycin as positive control.

compounds	A549	HeLa	K562	HL-60
**1**	>100	17.9	8.4	>100
**2**	>100	>100	>100	>100
(+)-**2**	>100	>100	>100	>100
(−)-**2**	>100	>100	>100	>100
**3**	82.7	>100	3.7	2.2
(+)-**3**	>100	53.7	47.2	22.7
(−)-**3**	>100	53.2	53.2	5.1
**4**	>100	>100	>100	>100
(+)-**4**	>100	42.7	88.4	54.2
(−)-**4**	>100	40.0	>100	>100
Adramycin	0.6	0.6	0.2	0.3

### Proposed Biogenesis

**Figure 4:**
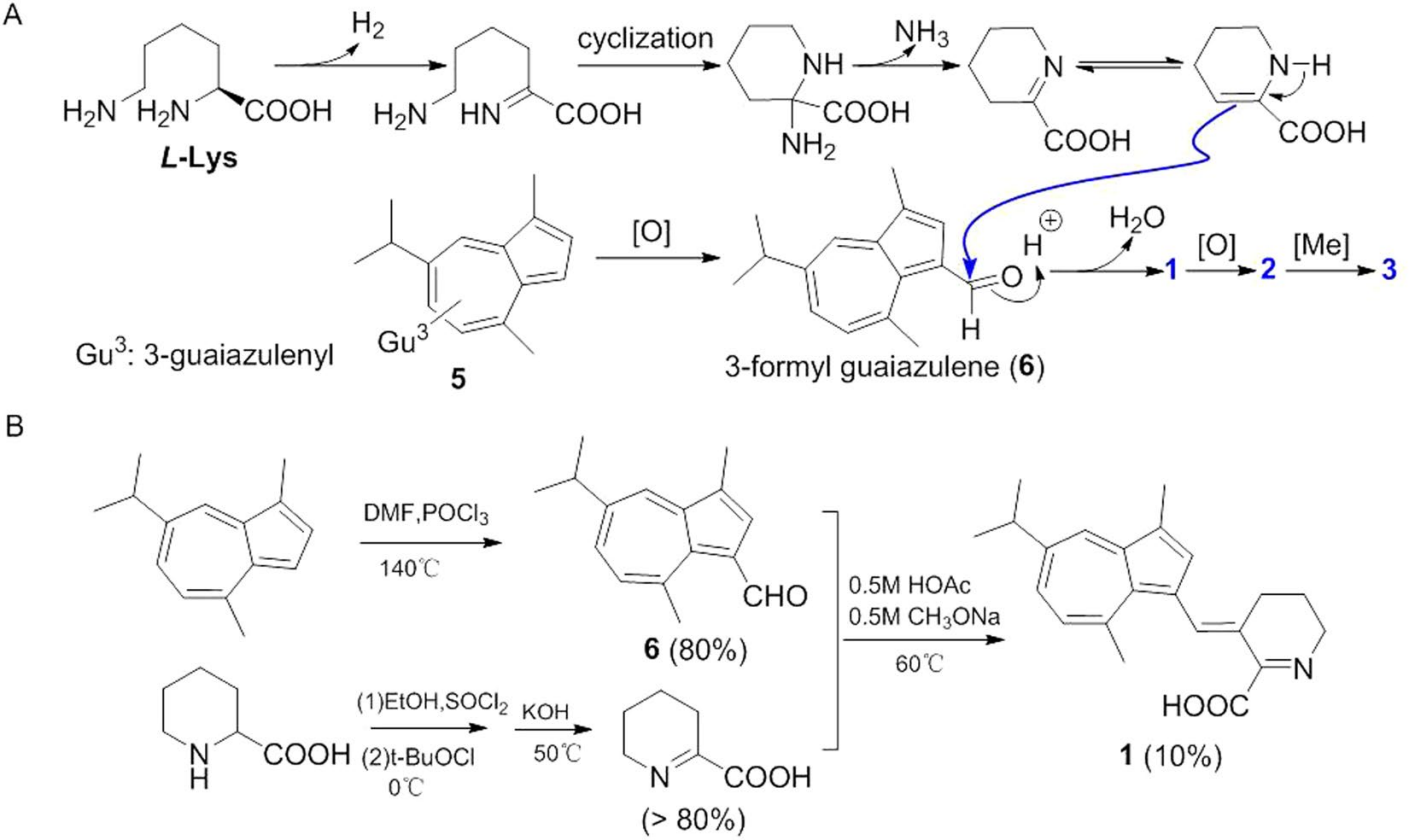
Biosynthetic pathway and semisynthetic procedure of the new compounds **1**–**3**. (**A**) Proposed Biosynthetic Pathway for Compounds **1**–**3**. (**B**) Semisynthetic procedure of muriceidine A (**1**).

Chemically, GA is known as having rich-electronic property and high reactivity, especially the diverse oxygenation has been proved to be a normal chemical transformation in many synthetic and natural products^10, 22, 23^. In the present study, the GA showed a unusual derivative pattern featured by coupling to Δ^1^-pipecolic acid (3,4,5,6-tetrahydropyridine-2-carboxylic acid) and indene moiety. From a biogenic view, Δ^1^-pipecolic acid was considered to be generated from *L*-Lys^24^, and the important precursor molecules as a part of the new skeleton, the co-isolated 3-formyl guaiazulene (**6**), could be derived from the co-isolated GA (**5**) through oxygenations. Δ^1^-pipecolic acid and **6** were speculated to form muriceidines A by an aldol-condensation-like step. Muriceidines B and C were regarded to be the products of oxidation and following methylation in allylic position of muriceidine A. Therefore, a plausible biogenetic pathway for these unusual guaiazulene derivatives (**1**–**3**) was proposed as shown in Fig. 4A.

### Semisynthesis of muriceidines A and B

In order to validate the proposed hypotheses, brief investigations were carried out using a precursor-directed approach (Fig. 4B). Firstly, the Δ^1^-pipecolic acid was synthesized by L-pipecolinic acid after esterification, chlorination and dehydrochlorination^25, 26^. The other precusor 3-formyl guaiazulene could be easily prepared by Vilsmeier-Hacck reaction starting with guaiazulene in 80% yield^27^. Under the presence of sodium methoxide and acetic acid, muriceidine A could be obtained by an aldol-condensation-like reaction between the Δ^1^-pipecolic acid and 3-formyl guaiazulene in 8% yield^28^, which not only proved the rationality of the proposed biogenetic pathway but also afforded an available reference for large-scale preparation of the family molecules. Furthermore, the further oxidation, either direct exposure to air, even using relative severe oxylation conditions of SeO_2_, H_2_O_2_ or bromination-hydrolysis with NBS-NaHCO_3_, in allylic position of muriceidine A to get muriceidines B and C failed. These efforts also indirectly confirmed the natural occurrence of (±)-Muriceidine B and (±)-Muriceidine C.

## Experimental Section

### General Experimental Procedures

Optical rotations were measured on a Jasco P-1020 digital polarimeter. UV spectra were recorded on a Beckman DU640 spectrophotometer. CD spectra were obtained on a Jasco J-810 spectropolarimeter. IR spectra were taken on a Nicolet NEXUS 470 spectrophotometer in KBr discs. NMR spectra were measured by JEOL JNMECP 600 and Bruker AVANCE III 600 spectrometers. The 7.2600 and 3.3100 ppm resonances of residual CDCl_3_ and CD_3_OD, and 77.16 and 49.00 ppm resonances of CDCl_3_ and CD_3_OD were used as internal references for ^1^H and ^13^C NMR spectra, respectively. HRESIMS spectra were measured on a Micromass Q-Tof Ultima GLOBAL GAA076 LC and Thermo Scientific LTQ orbitrap XL mass spectrometers. Semi-preparative HPLC utilized an ODS column [YMC-Pack ODS-A, 10 × 250 mm, 5 *μ*m, 1.5 mL/min]. Chiral HPLC utilized chiral analytical columns [Daicel Chiralpack IB and IC: 5 *μ*m, 4.6 mm × 250 mm]. Silica gel (200–300 mesh, Qingdao, China) was used for column chromatography, and pre-coated Silica gel plates (GF254, Qingdao, China) were used for TLC, and spots visualized by heating SiO_2_ plates sprayed with 5% H_2_SO_4_ in EtOH.

### Animal Material

The marine gorgonian *Muriceides collaris* was collected off the coast of Weizhou Island of South China Sea South Sea in April 2010, and was frozen immediately after collection. The specimen was identified by Prof. *Lin-Ren Zou*, South China Sea Institute of Oceanology, Chinese Academy of Sciences. The voucher specimen (No. WZD-2010-04) was deposited at State Key Laboratory of Marine Drugs, Ocean University of China, P. R. China.

### Extraction and isolation

A frozen specimen of *Muriceides collaris* (9.6 kg, wet weight) was homogenized and then extracted with MeOH four times (each time, 3 days) at room temperature. The combined solutions were concentrated *in vacuo* and the concentrated extract was subsequently desalted by redissolving with MeOH to yield a residue (221 g). The crude extract was subjected to silica gel vacuum liquid chromatography (VLC), eluting with a gradient of petroleum/acetone (from 10:0 to 1:1, v:v) and subsequently CH_2_Cl_2_/MeOH (from 20:1 to 0:1, v-v) to obtain nine fractions (Fr.A−Fr.I). Each fraction was detected by TLC, and was tested for their cytotoxicity against HeLa and K562 cell lines at 50 *μ*g/mL. The unusual spots with natural pink color and strong ultraviolet absorption (254 nm) on UV analyzer were observed on TLC of the most bioactive fraction Fr.G (CH_2_Cl_2_/MeOH 20:1) with inhibition rations of 89.0% and 92.8% against HeLa and K562 tumor cell lines, respectively. Fr.G (3.5 g) was then subjected to a Sephadex LH-20 column eluted with CH_2_Cl_2_/MeOH (1:1, v/v) to give three subfractions Fr.G-1 and Fr.G-4. Fr.G-3 (1.018 g) was separated by silica gel CC (CH_2_Cl_2_/MeOH, 30:1, v-v) to give six subfractions (Fr.G-3-1 to Fr.G-3-6). Fr.G-3-2 (223 mg) was purified by ODS CC with a gradient of MeOH/H_2_O (from 20% to 100%) to yield four subfractions (Fr.G-3-2-1 to Fr.G-3-2-4). Fr.G-3-2-3 (25.0 mg) was purified by semi-preparative HPLC (ODS, 5 μm, 250 × 10 mm; MeOH/H_2_O, 65:35, v/v; 1.5 mL/min) to afford muriceidines A (**1**, 10.3 mg) and B (**2**, 9.6 mg). Fr.G-3-4 (137 mg) was purified by ODS column chromatograph with a gradient of MeOH/H_2_O (from 20% to 100%) to yield four subfractions (Fr.G-3-4-1 to Fr.G-3-4-4). And Fr.G-3-4-1 (9.6 mg) was further purified by semi-preparative HPLC (ODS, 5 μm, 250 × 10 mm; MeOH/H_2_O, 60:40, v/v; 1.5 mL/min) to afford muriceidine C (**3**, 4.6 mg). Similarly, muriceidone A (**4**, 3.7 mg) and the co-isolated known 3-formylguaiazulene (**6**, 9.1 mg) was obtained in subfraction Fr. D. (4.9 g) with medium inhibition ratios of 69.4% and 51.9% against HeLa and K562 cell lines, repectively. And GA (**5**, 1.5 mg) was obtained in subfraction Fr. A (0.24 g) with only low inhibition ratio of 31.4% against HeLa cell line. Chiral seperations of partial **2** [Daicel Chiralpack IB, 5 *μ*m, 4.6 mm × 250 mm], **3** [Daicel Chiralpack IC, 5 *μ*m, 4.6 mm × 250 mm; MeOH, 1.5 mL/min], and **4** [Daicel Chiralpack IC, 5 *μ*m, 4.6 mm × 250 mm; Hexane/isopropyl alcohol 97:3, 1.5 mL/min] were performed on Agilent analytical HPLC system to afford optically pure (+)-**2** (0.9 mg), (−)-**2** (1.2 mg), (+)-**3** (2.5 mg), (−)-**3** (2.2 mg), (+)-**4** (1.5 mg), and (−)-**4** (1.6 mg) (Fig. S1).

Muriceidine A (**1**). dark red amorphous power; UV (MeOH) (log *ε*) *λ*_max_ 205 (3.23), 235 (3.38), 305 (3.04), 351 (3.11); IR (KBr)*ν*_max_ 2957, 2863, 1701, 1669, 1649, 1640, 1621, 1557, 1539, 1511, 1493, 1391, 1337, 1260, 1189, 1169, 1034, 859, 761 cm^−1^; ^1^H and ^13^C data, see Table 1; ESIMS *m*/*z* 292.2 [M − CO_2_ + H]^+^, 336.1 [M + H]^+^, *m*/*z* 358.2 [M + Na]^+^ ; HRESIMS *m*/*z* 336.1959 [M + H]^+^ (calcd for C_22_H_26_NO_2_, 336.1958), *m*/*z* 358.1776 [M + Na]^+^ (calcd for C_22_H_25_NO_2_Na, 358.1778).

(±)-Muriceidine B (**2**). dark red amorphous power; UV (MeOH) (log *ε*) *λ*_max_ 207 (2.70), 237 (2.76), 308 (2.45), 354 (2.48); IR (KBr)*ν*_max_ 3747,2958, 2926, 1689, 1682, 1649, 1556, 1539, 1509, 1520, 1493, 1458, 1390, 1337, 1263, 1171, 1097, 1933, 794, 666 cm^−1^; ^1^H and ^13^C data (Table 1); ESIMS *m*/*z* 308.2 [M - CO_2_ + H]^+^, 352.2 [M + H]^+^; HRESIMS *m*/*z* 352.1912 [M + H]^+^ (calcd for C_22_H_26_O_3_N, 352.1907), *m*/*z* 374.1732 [M + Na]^+^ (calcd for C_22_H_25_NO_3_Na, 374.1727). (+)-(**2**): [*α*]^25^_D_ 501.0 (*c* 0.017, MeOH); CD (*c* 0.0008 M, MeOH) *λ*_max_ (*Δε*) 199.1 (−7.8), 232.8 (3.4), 263.3 (−9.3), 302.0 (4.3), 347.3 (3.6) nm; (−)-(**2**): [*α*]^25^_D_ −586.7 (*c* 0.038, MeOH); CD (*c* 0.08 M, MeOH) *λ*_max_ (*Δε*) 193.3 (7.5), 231.8 (−3.1), 263.6 (6.1), 300.8 (−3.3), 348.8 (−2.3) nm.

(±)-Muriceidine C (**3**). dark red amorphous powder; UV (MeOH) (log *ε*) *λ*_max_ 207 (3.00), 236 (3.11), 293 (2.94), 352 (2.82); IR (KBr)*ν*_max_ 2957, 2927, 1700, 1682, 1650, 1557, 1539, 1509, 1458, 1433, 1419, 1391, 1366, 1337, 1264, 1169, 691, 669 cm^−1^; ^1^H and ^13^C data (Table 1); ESIMS *m*/*z* 322.2 [M - CO_2_ + H]^+^, 366.1 [M + H]^+^, *m*/*z* 388.2 [M + Na]^+^; HRESIMS *m*/*z* 388.1890 [M + Na]^+^ (calcd for C_23_H_27_NO_3_Na, 388.1883). (+)-(**3**): [*α*]^25^_D_ 662.0 (*c* 0. 025, CDCl_3_); CD (*c* 0.0008 M, MeOH) *λ*_max_ (*Δε*)) 199.1 (−2.3), 232.2 (2.6), 262.8 (−3.7), 304.7 (0.6), 341.6 (1.7) nm; (−)-(**3**): [*α*]^25^_D_ −506.6 (*c* 0. 053, CDCl_3_); CD (*c* 0.0007 M, MeOH) *λ*_max_ (*Δε*) 197.4 (2.3), 221.0 (−2.2), 267.4 (3.5), 300.9 (−1.1), 335.7 (−2.3) nm.

(±)-Muriceidone A (**4**). dark red amorphous powder; UV (MeOH) (log *ε*) *λ*_max_ 217 (3.49), 253 (3.24); IR (KBr)*ν*_max_ 3621, 2958, 1701, 1697, 1695, 1649, 1556, 1539, 1520, 1509, 1457, 1432, 1420, 1337, 1232, 1107 cm^−1^; ^1^H and ^13^C data (Table 3); ESIMS *m*/*z* 481.2 [M + Na]^+^; HRESIMS *m*/*z* 481.2360 [M + Na]^+^ (calcd for C_30_H_34_O_4_Na, 481.2349). (+)-(**4**): [*α*]^25^_D_ 308.7 (*c* 0.14, MeOH); CD (*c* 0.0013 M, MeOH) *λ*_max_ (*Δε*) 210.2 (31.4), 235.4 (−80.2), 281.9 (28.5), 368.4 (15.8) nm; (−)-(**4**): [*α*]^25^_D_ −316.7 (*c* 0.12, MeOH); CD (*c* 0.0008 M, MeOH) *λ*_max_ (*Δε*) 193.8 (−27.0), 241.6 (26.8), 284.9 (−18.6), 365.6 (−10.9) nm.

### Computational Section

The quantum chemical calculations were performed by using the density functional theory (DFT) as carried out in the Gaussian 09^29^. Conformational search was performed by Spartan 10 software using MMFF force filed, and conformers occurring within a 10 kcal/mol energy window from the global minimum were chosen for geometry optimization in the gas phase with the DFT method at the B3LYP/6-31G(d,p) and B3LYP/DGDZVP levels. The stable conformers for **1** were used in ^13^C NMR shifts calculations using DFT-GIAO model at RB3LYP/6-311+G(2d,p) level in chloroform by using the SCRF/PCM model in agreement with the experiment condition^16^. The experimental, calculated ^13^C NMR shifts (relative to TMS-resonance calculated at the same level of DFT) combined after Boltzmann weighting. The spin-allowed excitation energies and rotatory (*R*n) and oscillator strengths (*f*n) of the lowest excited states of stable conformers were calculated for ECD spectra using TD-DFT method with the basis set RB3LYP/DGDZVP^17^. Solvent effects of methanol solution were evaluated at the same DFT level by using the SCRF/PCM method. Electronic transitions were expanded as Gaussian curves with a FQHM (full width at half maximum) for each peak of 0.32 eV. The ECD spectra were combined after Boltzmann weighting according to their population contribution. And the optical rotation values of the dominant B3LYP/6-31G(d,p)-optimized geometries were calculated at RB3LYP-SCRF(PCM, methanol)/6-311+G(2d,p) level (Figs S6–S9 and Table S18 in Supporting Information)^17^.

### Cytotoxicity assay

The cytotoxicity of Fr. A–I against K562 and HeLa cell lines and compounds **1**–**4** against K562, HeLa, A-549, and HL-60 human tumor cell lines were determined by MTT method with Adriamycin as a positive control^20^. The IC_50_ value of each compound was calculated by Reed and Muench’s method. Experiments were repeated three times and carried out in triplicate.

### Antifouling assay

The antifouling activity of compounds **1**–**6** against against larval of the barnacle *Balanus albicostatus* Pilsbry were determined according to the literature^21^. EC_50_ was calculated as the concentration where 50% of the larval population was inhibited to settle as compared to the control while LC_50_ was calculated as the concentration where 50% of the larval population was dead. Experiments were repeated three times and carried out in triplicate.

## Electronic supplementary material

Supporting Information
