# Supplementary material for: Unusual Inner-Salt Guaiazulene Alkaloids and bis-Sesquiterpene from the South China Sea Gorgonian Muriceides collaris
Source: Sci Rep. 2017 Aug 9;7:7697. doi: 10.1038/s41598-017-08100-z (PMC5550455; doi:10.1038/s41598-017-08100-z)
Supplement: Supplementary file 1 — Supporting Information [file 41598_2017_8100_MOESM1_ESM.pdf]

Supporting Information

Unusual Inner-Salt Guaiazulene Alkaloids and *bis*-Sesquiterpene from the South China Sea Gorgonian *Muriceides collaris*

Pinglin Li[1,2,†], Xiaoling Liu[1,2,†], Hongyan Zhu[1,2,†], Xuli Tang[3], Xuefeng Shi[1,2], Yonghong Liu[4], and Guoqiang Li[1,2]

[1] Key Laboratory of Marine Drugs, Chinese Ministry of Education, School of Medicine and Pharmacy, Ocean University of China, Qingdao 266003, China.

[2] Laboratory of Marine Drugs and Biological Products, National Laboratory for Marine Science and Technology, Qingdao 266235, P. R. China.

[3] College of Chemistry and Chemical Engineering, Ocean University of China, Qingdao 266100, China.

[4] CAS Key Laboratory of Tropical Marine Bio-resources and Ecology, Guang dong Key Laboratory of Marine Materia Medica, Research Center for Marine Microbes, South China Sea Institute of Oceanology, Chinese Academy of Sciences, Guangzhou 510301, China.

Table of contents

Experimental Section		S3
1. Chiral HPLC purification of compounds **2-4**		S3
2. 1D NMR Data of compounds **5** and **6**		S3
3. Semisynthesis of muriceidine A (**1**)		S4
Computational details		S7
1. ^{13}C NMR calculation for **1** to distinguish inner-salt and non-ionized structures		S7
2. ECD calculations for **2** to determine the absolute configurations		S13
3. ECD and OR calculations for **4** to determine the absolute configurations.		S17
1D and 2D NMR spectra of the new compounds (**1**–**4**)		S28
SS1	The positive ESIMS spectrum of muriceidine A (**1**)	S28
SS2	The positive HRESIMS spectrum of muriceidine A (**1**)	S29
SS3	^{1}H NMR (600 MHz, $CDCl_3$) spectrum of muriceidine A (**1**)	S30
SS4	The amplificatory ^{1}H NMR spectrum of muriceidine A (**1**)	S31
SS5	^{13}C NMR (150 MHz, $CDCl_3$) spectrum of muriceidine A (**1**)	S32
SS6	DEPT spectrum of muriceidine A (**1**)	S33
SS7	^{1}H-^{1}H COSY spectrum of muriceidine A (**1**)	S34
SS8	HMQC spectrum of muriceidine A (**1**)	S35
SS9	HMBC spectrum of muriceidine A (**1**)	S36
SS10	The key amplificatory HMBC spectrum of muriceidine A (**1**)	S37
SS11	NOESY spectrum of muriceidine A (**1**)	S38
SS12	The positive ESIMS spectrum of muriceidine B (**2**)	S39
SS13	The positive HRESIMS spectrum of muriceidine B (**2**)	S40
SS14	^{1}H NMR (600 MHz, MeOD) spectrum of muriceidine B (**2**)	S41
SS15	The amplificatory ^{1}H NMR spectrum of muriceidine B (**2**)	S42
SS16	^{13}C NMR (150 MHz, MeOD) spectrum of muriceidine B (**2**)	S43
SS17	DEPT spectrum of muriceidine B (**2**)	S44
SS18	^{1}H-^{1}H COSY spectrum of muriceidine B (**2**)	S45
SS19	HMQC spectrum of muriceidine B (**2**)	S46
SS20	HMBC spectrum of muriceidine B (**2**)	S47
SS21	The key amplificatory HMBC spectrum of muriceidine B (**2**)	S48
SS22	NOESY spectrum of muriceidine B (**2**)	S49
SS23	The positive ESIMS spectrum of muriceidine C (**3**)	S50
SS24	The positive HRESIMS spectrum of muriceidine C (**3**)	S51
SS25	^{1}H NMR (600 MHz, $CDCl_3$) spectrum of muriceidine C (**3**)	S52
SS26	The amplificatory ^{1}H NMR spectrum of muriceidine C (**3**)	S53
SS27	^{13}C NMR (150 MHz, $CDCl_3$) spectrum of muriceidine C (**3**)	S54
SS28	DEPT spectrum of muriceidine C (**3**)	S55
SS29	^{1}H-^{1}H COSY spectrum of muriceidine C (**3**)	S56
SS30	HMQC spectrum of muriceidine C (**3**)	S57
SS31	HMBC spectrum of muriceidine C (**3**)	S58
SS32	NOESY spectrum of muriceidine C (**3**)	S59
SS33	The positive ESIMS spectrum of muriceidone A (**4**)	S60
SS34	The positive HRESIMS spectrum of muriceidone A (**4**)	S61
SS35	^{1}H NMR (600 MHz, $CDCl_3$) spectrum of muriceidone A (**4**)	S62
SS36	The amplificatory ^{1}H NMR spectrum of muriceidone A (**4**)	S63
SS37	^{13}C NMR (150 MHz, $CDCl_3$) spectrum of muriceidone A (**4**)	S64
SS38	The amplificatory ^{13}C NMR spectrum of muriceidone A (**4**)	S65
SS39	DEPT spectrum of muriceidone A (**4**)	S66
SS40	HMQC spectrum of muriceidone A (**4**)	S67
SS41	HMBC spectrum of muriceidone A (**4**)	S68
SS42	The key amplificatory HMBC spectrum of muriceidone A (**4**)	S69
SS43	NOESY spectrum of muriceidine C (**4**)	S70
SS44	The amplificatory NOESY spectrum of muriceidine C (**4**)	S71

Experimental Section

1. Chiral *HPLC purification of compounds 2–4:*

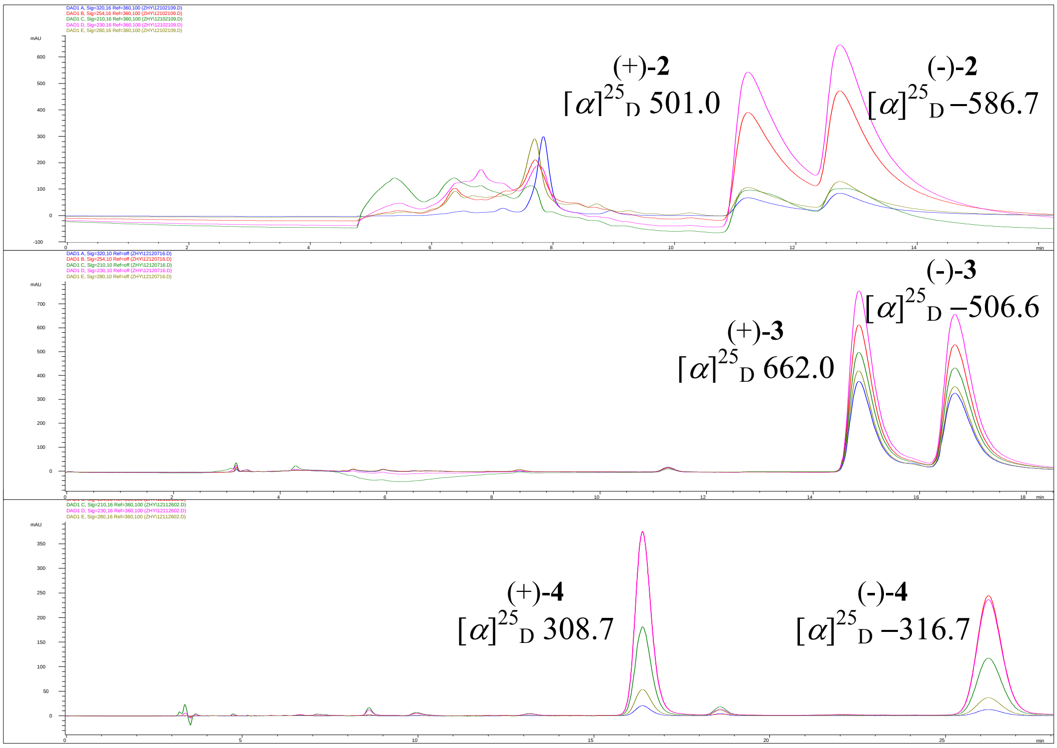

Figure S1. Chiral HPLC seperations of compounds **2**–**4**.

2. *1D NMR Data of the compounds 5, 6 and synthetic muriceidine A:*

Guaiazulene (GA, 5): Azure amorphous powder; ESI-MS *m/z* 199.1 $[M+H]^+$; ^{1}H NMR ($CDCl_3$, 600 MHz) δ_H: 8.20 (1H, s, H-4), 7.62 (1H, d, *J* = 3.7 Hz, H-1), 7.41 (1H, d, *J* = 10.3 Hz, H-6), 7.22 (1H, d, *J* = 3.3 Hz, H-2), 7.01 (1H, d, *J* = 10.6 Hz, H-7), 3.08 (1H, m, H-11), 2.83 (3H, s), 2.67 (3H, s, H-14), 1.36 (6H, d, *J* = 7.0 Hz, H-12/13); ^{13}C NMR ($CDCl_3$, 150 MHz) δ_C: 144.3 (s, C-8), 139.9 (s, C-5), 137.2 (s, C-9), 136.2 (s, C-10), 136.1 (d, C-1), 134.9 (d, C-6), 133.3 (d, C-4), 125.2 (s, C-3), 125.0 (d, C-7), 112.7 (d, C-2), 38.3 (d, C-11), 24.8 (q, C-12/13), 24.1 (q，C-15), 12.9 (q, C-14).

***3-formylguaiazulene (6)*:** Purple amorphous powder; ^{1}H NMR ($CDCl_3$, 600 MHz): δ_H 10.64 (1H, s, CHO), 8.29 (1H, d, *J* = 2.2 Hz, H-8), 8.23 (1H, s, H-2), 7.60 (1H, dd, *J* = 11.0, 2.2 Hz, H-6), 7.43 (1H, d, *J* = 11.0 Hz, H-5), 3.17 (1H, dq, *J* = 7.1, 7.1 Hz, H-11), 2.59 (3H, s, H-15), 2.27 (3H, s, H-14), 1.39 (6H, d, *J* = 7.1 Hz, H-12/13).

Synthetic muriceidine A: ^{1}H NMR ($CDCl_3$, 600 MHz): δ_H 1.36 (6H, d, *J* = 6.86 Hz), 1.98 (2H, br s), 2.56 (3H, s), 2.87 (2H, br s), 3.07 (1H, m), 3.14 (3H, s), 3.70 (2H, br s), 7.31 (1H, d, *J* = 10.67 Hz), 7.49 (1H, d, *J* = 10.72 Hz), 7.76 (1H, s), 8.14 (1H, s), 9.56 (1H, br s). ^{13}C NMR ($CDCl_3$, 150 MHz): δ_C 13.3, 20.3, 24.5, 29.7, 38.1, 42.8, 117.9, 123.6, 128.3, 133.7, 134.7, 137.2, 139.1, 141.2, 143.0, 147.0, 149.4, 149.5, 164.0, 171.4.

3. Semisynthesis of muriceidine A (1):

Et_2O and DMF were purified by distillation. ^1H and ^{13}C NMR spectra were recorded on a JEOL JNMECP 500 spectrometer (JEOL Ltd., Tokoyo, Japan), using $CDCl_3$ or CD_3OD as the solvent. HRESIMS spectra were acquired using a Micromass Q-TOF Ultima Global GAA076 LC mass spectrometer (Thermo Fisher Scientific Inc., Waltham, MA, USA).All reactions were routinely followed by thin-layer chromatography on commercial TLC precoated silica gel plates (GF254, Qingdao Haiyang Chemical Co. Ltd., Qingdao, China)). Silica gel (300－400 mesh, Qingdao Haiyang Chemical Co. Ltd., Qingdao, China) and ODS silica gel (50 μm, Merck, Darmstadt, Germany) were used for column chromatography (CC). The analysis of intermediates and products were performed on an Agilent 1100 series instrument with DAD detector (Agilent Technologies, Palo Alto, CA, USA), equipped with a semi-preparative ODS column (YMC-Pack ODS-A, 5μm, 250 × 10 mm). UV spectra were measured on a Beckman DU640 spectrophotometer (Beckman Coulter Inc., Brea, CA, USA). All the solvents were evaporated under reduced pressure using a rotary evaporator.

Synthetic route:

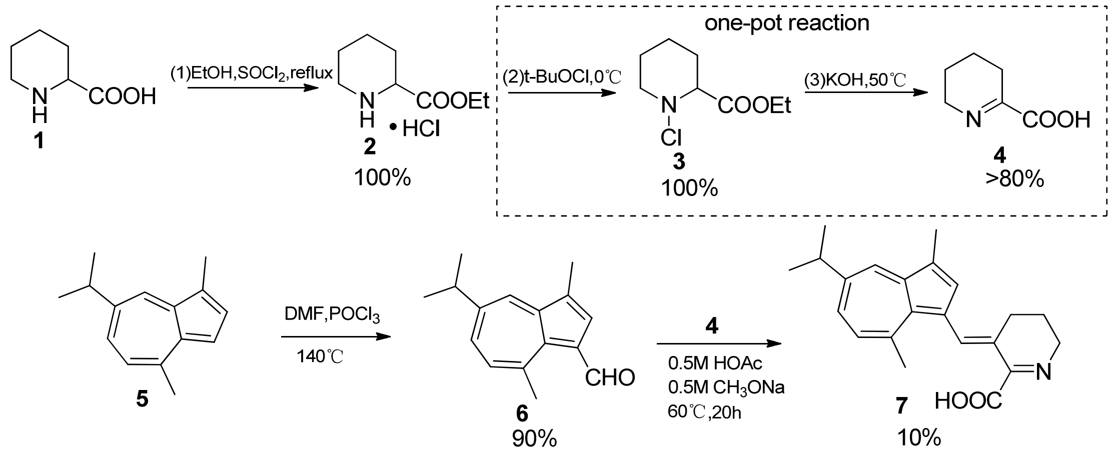

Synthesis of compound **2**.

L-pipecolic acid (1.3g,100mmol) was dissolved in 40 mL of anhydrous ethanol, and $SOCl_2$ (7.3mL,100mmol) was added dropwise at 0℃. Then the mixture was refluxed for 4 h under stirring. After the reaction had ceased, the solvent was removed in vacuo. The residue was resolved in 5 mL of MeOH, adding $NaHCO_3$ gradually until neutral pH. The mixture was filtered and concentrated at reduced pressure to yield compound **2** as a white solid (1.6 g,100%). ^1H-NMR (500 MHz, CD_3OD) δ 4.20-4.24 (q, 7.1Hz,2H), 4.00 (dd, J = 11.5、3.4 Hz, 1H), 3.37 (dt, J = 12.6 Hz, 1H), 3.27 (m, 1H), 3.03 (td, J = 12.3、3.3 Hz, 1H), 2.27-2.22 (m, 1H), 1.91 -1.80 (m, 2H), 1.76-1.59 (m, 3H), 1.29 (t, J = 7.1 Hz, 3H). ^{13}C-NMR (126 MHz, CD_3OD) δ 169.74, 63.56, 57.78, 48.86, 45.10, 27.06, 22.85, 22.69, 14.21.

Synthesis of compound **3** and **4**

t-Butyl hypochlorite: (Caution! This procedure should be conducted in dim light and exposure to the hypochlorite should be avoided. The product should not be exposed to direct sun light or rubber. Do not heat the product over its boiling point (77-78 ℃ at 760 mm Hg). In a 25 mL round-bottom flask a mixture of t-BuOH (2 mL, 20.9 mmol), aqueous NaOCl solution(active chlorine ≥5% ,12mL,18.6mmol), and acetic acid (2 mL, 34.3 mmol) was added in order and stirred at 0 ℃ for 5 minutes. The desired product was isolated from the lower aqueous phase as a yellow liquid. The organic fraction was directly used as chlorine source in the next step without further purification.

N-Chloropipeclic acid ester: Compound **2** (400 mg, 2.1 mmol) was dissolved with 15 mL of anhydrous ether in a 50 mL flask, and the solution was added the freshly prepared t-BuOCl dropwise at 0℃ under N_2 atmosphere. Then the mixture was stirred at 0℃ for 3h. TLC tracking monitoring

and ninhydrin colorimetry method were used till the reactant is fully converted. The N-chloropipecolic acid ester was quite unstable and sensitive to light. So after the reaction had ceased, excessive solid KOH (500mg, 8.9 mmol) was added in one batch, followed by 10 mL dry MeOH. The mixture was heated in an oil bath at 50℃ for 1h and then kept at room temperature overnight. The mixture was concentrated by rotary evaporation at 20℃ to remove the ether and get the methanol solution of compound **4** ($\triangle^1$-pipecolic acid). The white solid was removed by filtration, and this crude material was used in the next step without further purification. TLC proved that compound **4** existed as a near equimolar tautomeric mixture of enamine and imine. It was sensitive to the air and couldn't be kept without solvents.

Synthesis of compound **6**

$POCl_3$was added dropwise to stirred, cold DMF. The mixture was stirred at 0℃ for 0.5 h. Then a solution of commercially available guaiazulene (7-isopropyl-1,4-dimethylazulene) (**5**) (1.0 g,504 mmol) in DMF (10 mL) was added. The mixture was stirred at 0 ℃ for 1 h and then kept at room temperature overnight. After the reaction, the solution was poured into 50mL ice water, carefully neutralized with aq NaOH and then the resulting product was extracted with dichloromethane (50mL×3). The extract was washed with distilled water and evaporated in vacuo. The residue thus obtained was carefully separated by silica gel column chromatography with petroleum ether/acetone (5:1, vol/vol). The product was purified as a purple solid (108 mg, 477 mmol, 90% yield). ^{1}H-NMR (500 MHz, $CDCl_3$) δ 10.67 (s, 1H), 8.33 (d, J = 1.5 Hz, 1H), 8.26 (s, 1H), 7.62 (d, J = 10.8 Hz, 1H), 7.46 (d, J = 10.8 Hz, 1H), 3.22-3.16 (m, 1H), 3.18(s,3H), 2.62 (s, 3H), 1.43 (d, J = 6.9 Hz, 6H).

Synthesis of compound **7**

CH_3ONa (93 mg, 0.29 mmol) was dissolved in methanol (5 mL) in a 100mL flask. And then the methanol solution of compound **4** was added. The solution was stirred at room temperature for a while and then added 2mL methanol solution of compund **6** (mg, mmol) containing acetic acid (72 μL, 1.2 mmol) , and the mixture was added to the flask dropwise. Then the reaction was heated to 60 ℃ and stirred for 20h. After cooling, the solution was evaporated to remove the methnol. The residue was dissolved with distilled water and EtOAc .The EtOAc layer was separated from the water phase. The lower solution was extracted three times with EtOAc. The organic layers were combined and concentrated by evaporation. The crude product was purified with column chromatography (eluent: CH_2Cl_2/CH_3OH 15:1) to obtain a red solid (20mg, 10%).MS:$[M+H]^1$ =336. H-NMR (500 MHz, $CDCl_3$) δ 9.57 (s, 1H), 8.15 (s, 1H), 7.77 (s, 1H), 7.50 (d, J = 10.7 Hz, 1H), 7.32 (d, J = 10.7 Hz, 1H), 3.71 (s, 2H), 3.15 (s, 3H), 3.10 (m, 1H), 2.88 (s, 2H), 2.57 (s, 3H), 1.99 (s, 2H), 1.37 (d, J = 6.9 Hz, 6H). ^{13}C-NMR (126 MHz, CD_3OD) δ 170.93, 164.48, 149.25, 148.71, 146.76, 145.62, 142.79, 141.01, 138.85, 136.91, 134.48, 133.45, 128.06, 123.32, 117.63, 77.15, 76.90, 76.65, 42.58, 37.89, 29.47, 24.40, 24.30, 20.06, 13.06.

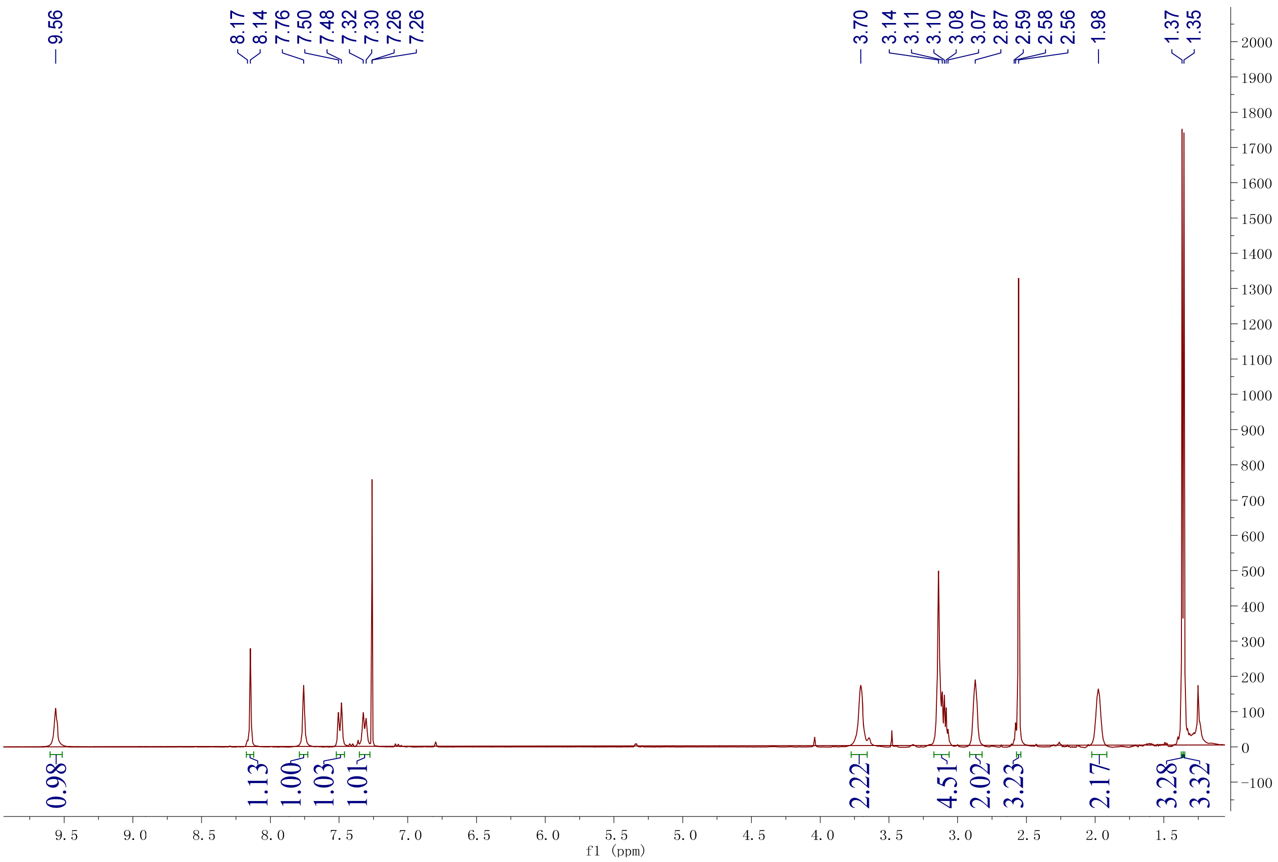

Figure C1 ^{1}H NMR (600 MHz, $CDCl_3$) spectrum of synthetic muriceidine A (**1**)

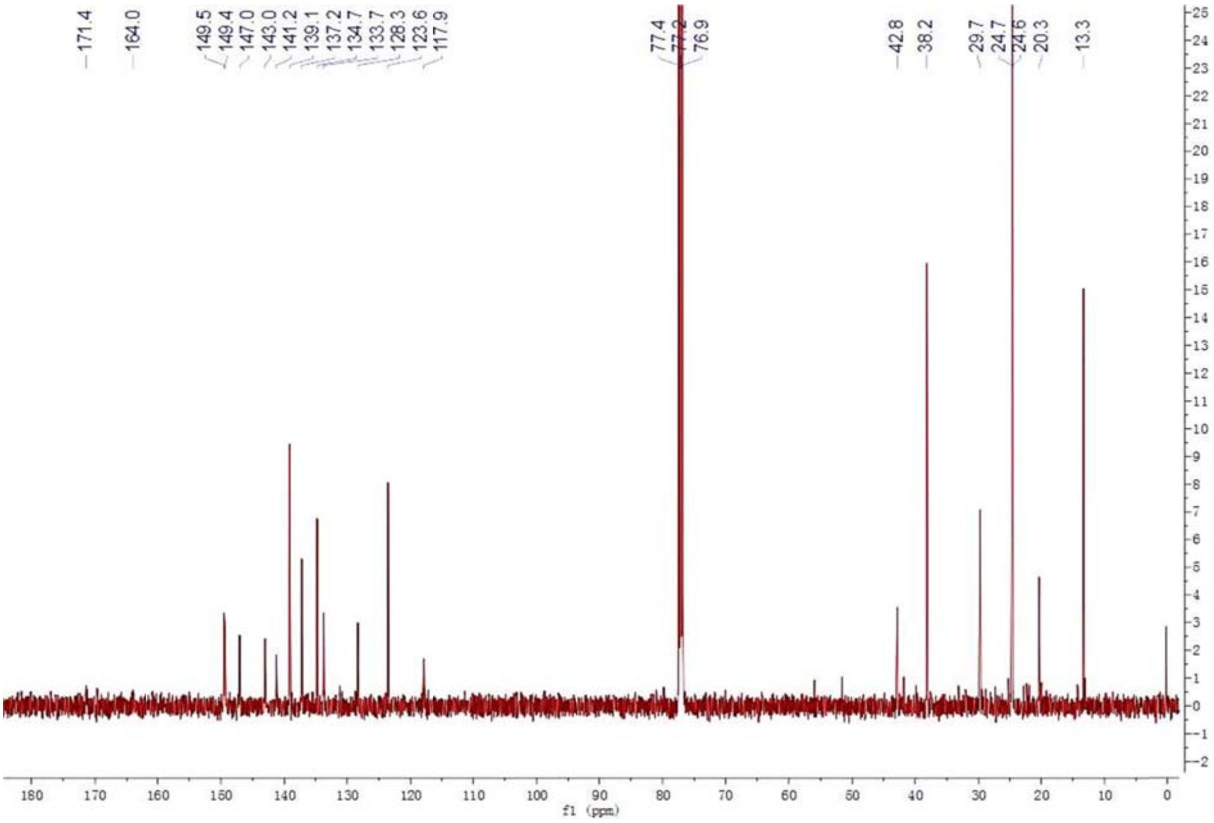

Figure C2 ^{13}C NMR (150 MHz, $CDCl_3$) spectrum of synthetic muriceidine A (**1**)

Computational details

1. ^{13}C NMR calculation for 1 to distinguish inner-salt and non-ionized structures

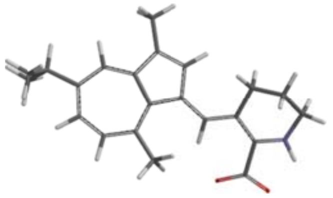

1a1 (12.1%)

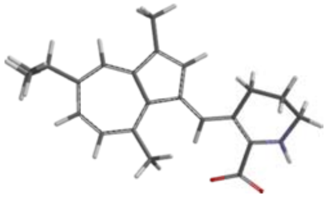

1a2 (12.1%)

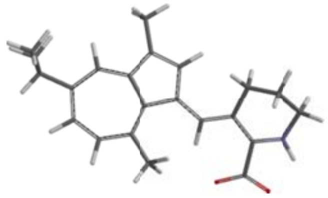

1a3 (10.8%)

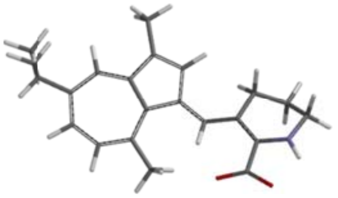

1a4 (10.8%)

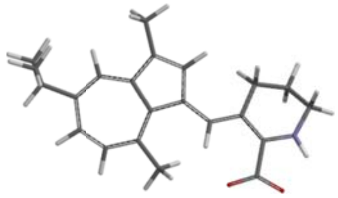

1a5 (10.8%)

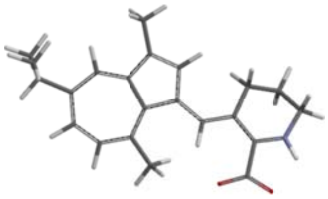

1a6 (10.8%)

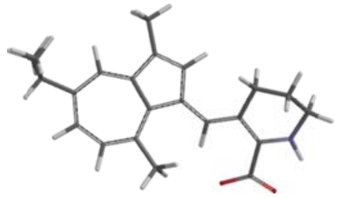

1a7 (10.8%)

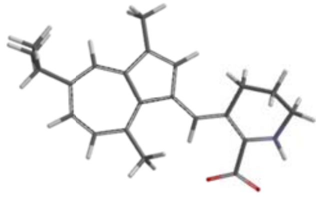

1a8 (10.8%)

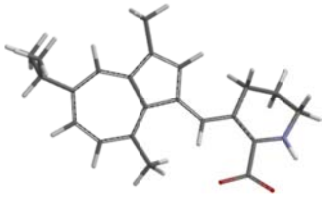

1a9 (10.7%)

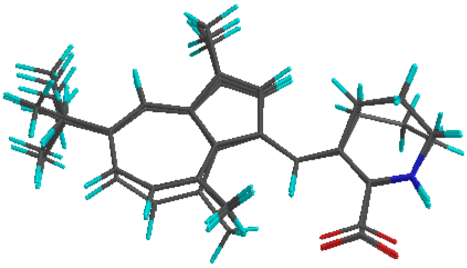

overlaid image

Figure S2 Stable conformers of compound **1** with inner salt structure.

Table S1. Important thermodynamic parameters (a.u.) of the optimized compound **1** with inner salt structure at B3LYP/DGDZVP level in the gas phase

conformations	E+ZPE	G
**1a1**	-1058.242494	-1058.296857
**1a2**	-1058.242494	-1058.296857
**1a3**	-1058.242413	-1058.296750
**1a4**	-1058.242413	-1058.296750
**1a5**	-1058.242413	-1058.296750
**1a6**	-1058.242413	-1058.296750
**1a7**	-1058.242413	-1058.296750
**1a8**	-1058.242413	-1058.296749
**1a9**	-1058.242414	-1058.296745

Table S2. Optimized Z-Matrixes of compound **1** with inner salt structure in the Gas Phase (Å) at B3LYP/DGDZVP level.

**1a1 1a2**								**1a3**			
C	-4.19779	-0.16623	0.001269	C	-4.19779	-0.16623	0.001263	C	4.193099	-0.5708	-0.06655
C	-3.77189	-1.4723	0.294981	C	-3.77189	-1.4723	0.294982	C	3.682058	-1.84485	-0.34569
C	-2.4975	-1.98884	0.5194	C	-2.4975	-1.98884	0.519414	C	2.369775	-2.28267	-0.54376
C	-1.19952	-1.42288	0.468899	C	-1.19952	-1.42287	0.468918	C	1.113733	-1.63896	-0.47379
C	-0.91454	-0.09059	0.10485	C	-0.91454	-0.09058	0.104865	C	0.919551	-0.28426	-0.12029
C	-1.92707	0.978709	-0.1054	C	-1.92707	0.978714	-0.10538	C	2.003122	0.718507	0.069363
C	-3.32387	0.915425	-0.14333	C	-3.32387	0.915428	-0.14333	C	3.389525	0.567215	0.087156
C	0.37521	0.546635	-0.02689	C	0.375209	0.54664	-0.02687	C	-0.32138	0.435904	0.022186
C	0.127465	1.936683	-0.26343	C	0.127465	1.936689	-0.26341	C	0.018814	1.810628	0.250526
C	-1.23032	2.207749	-0.31725	C	-1.23032	2.207755	-0.31723	C	1.388827	1.993536	0.286462
C	-5.69898	0.090969	-0.14942	C	-5.69898	0.090965	-0.14944	C	5.71079	-0.41404	0.055337
C	-6.47237	-0.19172	1.154753	C	-6.47238	-0.19172	1.154724	C	6.135873	0.063457	1.458994
C	-6.30332	-0.68698	-1.3362	C	-6.3033	-0.68699	-1.33623	C	6.291697	0.498486	-1.04416
C	-0.09551	-2.38928	0.858142	C	-0.09551	-2.38927	0.858173	C	-0.05796	-2.53735	-0.82692
C	-1.84862	3.552679	-0.5888	C	-1.84862	3.552685	-0.58878	C	2.097594	3.295508	0.545173
C	1.647991	-0.10399	-0.08547	C	1.647989	-0.10399	-0.08546	C	-1.63637	-0.12475	0.088945
C	2.924017	0.421793	0.024977	C	2.924018	0.421792	0.024978	C	-2.87209	0.489508	-0.02245
C	4.034356	-0.45999	-0.20209	C	4.034352	-0.46	-0.20209	C	-4.04174	-0.3096	0.214334
N	5.274586	-0.01063	-0.14338	N	5.274584	-0.01064	-0.1434	N	-5.24728	0.224889	0.150912
C	5.691507	1.346953	0.195013	C	5.691512	1.346946	0.194987	C	-5.56888	1.604101	-0.20386
C	4.614625	2.011728	1.049112	C	4.614639	2.011725	1.049094	C	-4.44789	2.182599	-1.06405
C	3.238582	1.86896	0.38176	C	3.23859	1.86896	0.381756	C	-3.08573	1.951884	-0.39253
C	4.041629	-2.03255	-0.54249	C	4.04162	-2.03256	-0.54249	C	-4.15935	-1.87375	0.573023
O	5.207897	-2.45429	-0.7266	O	2.941795	-2.61993	-0.56689	O	-5.35182	-2.20875	0.767361
O	2.941805	-2.61993	-0.56689	O	5.207886	-2.4543	-0.72661	O	-3.10416	-2.53771	0.598626
H	-4.56479	-2.21198	0.394348	H	-4.56478	-2.21198	0.394344	H	4.428637	-2.6312	-0.45463
H	-2.50285	-3.04336	0.786422	H	-2.50285	-3.04335	0.786441	H	2.307509	-3.33713	-0.80349
H	-3.80603	1.87273	-0.33693	H	-3.80603	1.872731	-0.33693	H	3.930457	1.494116	0.268218
H	0.895011	2.677253	-0.45164	H	0.895011	2.67726	-0.45162	H	-0.69781	2.598878	0.445013
H	-5.82003	1.158057	-0.36983	H	-5.82003	1.158052	-0.36986	H	6.14641	-1.40918	-0.09081
H	-6.0648	0.384102	1.992233	H	-6.06482	0.384102	1.992206	H	5.749229	-0.60022	2.23927
H	-6.43319	-1.25221	1.427533	H	-6.4332	-1.25221	1.427507	H	5.773002	1.075483	1.670273
H	-7.52773	0.078737	1.037649	H	-7.52774	0.07873	1.037608	H	7.22849	0.082627	1.539296
H	-5.77443	-0.46459	-2.26875	H	-6.25826	-1.77004	-1.17644	H	5.931531	1.528986	-0.94802
H	-6.25828	-1.77003	-1.17641	H	-5.77441	-0.4646	-2.26877	H	6.019713	0.141072	-2.04279
H	-7.35707	-0.41803	-1.46999	H	-7.35706	-0.41804	-1.47003	H	7.385164	0.524996	-0.97851
H	-0.51385	-3.25651	1.373896	H	0.636124	-1.92492	1.522413	H	-0.76127	-2.04233	-1.49996
H	0.636126	-1.92494	1.522386	H	0.461422	-2.76338	-0.00657	H	-0.63204	-2.84646	0.051857
H	0.461423	-2.76338	-0.00661	H	-0.51385	-3.25649	1.373936	H	0.294366	-3.44615	-1.31967
H	-1.07193	4.312801	-0.71238	H	-2.4536	3.552175	-1.50388	H	2.717985	3.255966	1.449046
H	-2.49902	3.880963	0.231283	H	-1.07193	4.312828	-0.71225	H	1.373857	4.103968	0.682407
H	-2.4537	3.552144	-1.50383	H	-2.49912	3.880921	0.231245	H	2.752328	3.581661	-0.28724
H	1.658374	-1.16777	-0.28324	H	1.658367	-1.16777	-0.28323	H	-1.7215	-1.1838	0.293486
H	5.940616	-0.77247	-0.35711	H	5.940608	-0.77249	-0.35713	H	-5.96463	-0.48568	0.375551
H	5.864045	1.913701	-0.73011	H	5.864043	1.913691	-0.73014	H	-6.52598	1.604917	-0.73449

H	6.646998	1.287604	0.7252	H	6.647007	1.287595	0.725166	H	-5.70198	2.192387	0.71426
H	4.862364	3.067499	1.199187	H	4.597402	1.535539	2.036207	H	-4.62138	3.251353	-1.22595
H	4.597381	1.535539	2.036223	H	4.862382	3.067496	1.199165	H	-4.46294	1.695517	-2.04586
H	2.470301	2.25656	1.057271	H	2.470315	2.256562	1.057274	H	-3.01716	2.585727	0.503324
H	3.210798	2.498066	-0.51965	H	3.210798	2.498064	-0.51965	H	-2.29176	2.280078	-1.06964
**1a4 1a5**								**1a6**			
C	-4.1931	-0.5708	-0.06655	C	-4.1931	-0.5708	-0.06655	C	4.193095	-0.5708	-0.06654
C	-3.68205	-1.84485	-0.34569	C	-3.68206	-1.84485	-0.34569	C	3.682051	-1.84485	-0.34568
C	-2.36977	-2.28266	-0.54376	C	-2.36977	-2.28266	-0.54377	C	2.369769	-2.28266	-0.54377
C	-1.11373	-1.63895	-0.4738	C	-1.11373	-1.63895	-0.4738	C	1.113731	-1.63894	-0.47382
C	-0.91955	-0.28425	-0.1203	C	-0.91955	-0.28425	-0.1203	C	0.919551	-0.28425	-0.12032
C	-2.00312	0.71851	0.069355	C	-2.00312	0.718514	0.069348	C	2.003124	0.71852	0.069332
C	-3.38952	0.567216	0.087155	C	-3.38953	0.567218	0.08715	C	3.389526	0.56722	0.087146
C	0.321374	0.435908	0.02218	C	0.321375	0.435911	0.022171	C	-0.32138	0.435919	0.022152
C	-0.01882	1.810634	0.250517	C	-0.01882	1.810637	0.250504	C	0.018817	1.810646	0.250478
C	-1.38883	1.993539	0.286449	C	-1.38883	1.993544	0.28644	C	1.388829	1.993552	0.286413
C	-5.71079	-0.41404	0.055342	C	-5.71079	-0.41405	0.055357	C	5.710785	-0.41405	0.055382
C	-6.13587	0.063447	1.459001	C	-6.29171	0.498474	-1.04413	C	6.135844	0.063449	1.459045
C	-6.29169	0.498488	-1.04414	C	-6.13586	0.063453	1.459017	C	6.29172	0.498464	-1.0441
C	0.057962	-2.53734	-0.82691	C	0.057962	-2.53733	-0.82694	C	-0.05796	-2.53732	-0.82697
C	-2.0976	3.295513	0.545158	C	-2.0976	3.295517	0.54515	C	2.097599	3.295525	0.54512
C	1.636362	-0.12475	0.08895	C	1.636363	-0.12475	0.088938	C	-1.63636	-0.12474	0.088928
C	2.872092	0.489513	-0.02244	C	2.872095	0.48951	-0.02245	C	-2.8721	0.489509	-0.02245
C	4.04173	-0.30959	0.214344	C	4.041732	-0.3096	0.214342	C	-4.04173	-0.3096	0.21435
N	5.247272	0.224883	0.150926	N	5.247274	0.22488	0.150932	N	-5.24727	0.224872	0.150954
C	5.568882	1.604094	-0.20384	C	5.568885	1.60409	-0.20383	C	-5.56889	1.604083	-0.2038
C	4.447898	2.182592	-1.06404	C	4.447906	2.182594	-1.06403	C	-4.44792	2.182591	-1.06401
C	3.085737	1.951886	-0.39252	C	3.085741	1.951884	-0.39253	C	-3.08575	1.951883	-0.39252
C	4.159333	-1.87375	0.573027	C	4.159332	-1.87376	0.573027	C	-4.15932	-1.87377	0.573029
O	5.35181	-2.20876	0.767345	O	5.351805	-2.20876	0.767361	O	-3.10413	-2.53772	0.598611
O	3.104151	-2.53771	0.598608	O	3.104146	-2.53771	0.59862	O	-5.35179	-2.20877	0.767371
H	-4.42863	-2.6312	-0.45462	H	-4.42863	-2.63121	-0.45461	H	4.428628	-2.63121	-0.45459
H	-2.3075	-3.33713	-0.80349	H	-2.30751	-3.33713	-0.8035	H	2.307501	-3.33712	-0.8035
H	-3.93046	1.494115	0.26822	H	-3.93046	1.494117	0.268213	H	3.93046	1.494118	0.268209
H	0.697805	2.598886	0.445005	H	0.69781	2.598888	0.444984	H	-0.69781	2.5989	0.444951
H	-6.14641	-1.40919	-0.09081	H	-6.14641	-1.40919	-0.09079	H	6.146401	-1.4092	-0.09076
H	-7.22849	0.08261	1.539303	H	-6.01973	0.141059	-2.04277	H	5.749179	-0.60022	2.239315
H	-5.74922	-0.60023	2.239273	H	-5.93155	1.528976	-0.948	H	5.772975	1.075479	1.67031
H	-5.773	1.075474	1.670283	H	-7.38518	0.524979	-0.97847	H	7.228459	0.082611	1.539368
H	-5.93153	1.528988	-0.948	H	-5.77299	1.075481	1.670289	H	5.93156	1.528967	-0.94798
H	-6.01971	0.141081	-2.04278	H	-5.74921	-0.60022	2.23929	H	6.019752	0.141047	-2.04274
H	-7.38516	0.524996	-0.97849	H	-7.22848	0.082619	1.539329	H	7.385186	0.524966	-0.97844
H	0.76128	-2.04234	-1.49996	H	0.632045	-2.84646	0.051831	H	-0.76127	-2.04228	-1.50001
H	-0.29436	-3.44616	-1.31965	H	0.761274	-2.04231	-1.49998	H	-0.63205	-2.84646	0.051788
H	0.632031	-2.84645	0.051874	H	-0.29436	-3.44614	-1.3197	H	0.294363	-3.44611	-1.31975
H	-2.75234	3.581655	-0.28724	H	-2.75237	3.581643	-0.28724	H	2.717909	3.256015	1.449051
H	-1.37386	4.103974	0.682374	H	-1.37386	4.103987	0.682331	H	1.373866	4.104006	0.682248

H	-2.71797	3.255973	1.449041	H	-2.71795	3.25599	1.449051	H	2.752413	3.581623	-0.28724
H	1.721493	-1.1838	0.293488	H	1.72149	-1.1838	0.293481	H	-1.72148	-1.18379	0.293472
H	5.964619	-0.4857	0.375567	H	5.964622	-0.4857	0.375576	H	-5.96462	-0.48571	0.3756
H	5.701976	2.192381	0.714274	H	5.701974	2.192375	0.71429	H	-5.70197	2.192364	0.714326
H	6.525991	1.604905	-0.73447	H	6.525997	1.604906	-0.73445	H	-6.52601	1.6049	-0.7344
H	4.621392	3.251345	-1.22595	H	4.462962	1.695511	-2.04584	H	-4.46299	1.69551	-2.04582
H	4.462948	1.695506	-2.04585	H	4.621404	3.251346	-1.22594	H	-4.62143	3.251343	-1.22591
H	3.01717	2.585733	0.503323	H	2.291781	2.280081	-1.06965	H	-2.2918	2.280081	-1.06966
H	2.291775	2.280081	-1.06964	H	3.017166	2.58573	0.503322	H	-3.01716	2.585729	0.503323
**1a7 1a8**								**1a9**			
C	4.193094	-0.5708	-0.06654	C	-4.19311	-0.5708	-0.06657	C	-4.19313	-0.57086	-0.06677
C	3.682051	-1.84485	-0.34568	C	-3.68207	-1.84485	-0.34569	C	-3.68204	-1.84484	-0.34617
C	2.369768	-2.28266	-0.54377	C	-2.36979	-2.28268	-0.54373	C	-2.36972	-2.28262	-0.54409
C	1.113731	-1.63895	-0.47381	C	-1.11374	-1.63898	-0.47374	C	-1.11367	-1.63892	-0.47384
C	0.919552	-0.28425	-0.12032	C	-0.91956	-0.28428	-0.12026	C	-0.91953	-0.28426	-0.12012
C	2.003124	0.718516	0.069338	C	-2.00313	0.718497	0.069385	C	-2.00317	0.718471	0.069711
C	3.389525	0.567219	0.087148	C	-3.38953	0.567212	0.087166	C	-3.38956	0.567129	0.087368
C	-0.32137	0.435916	0.022161	C	0.321373	0.435885	0.022218	C	0.321355	0.43594	0.022645
C	0.018818	1.810643	0.25049	C	-0.01882	1.810612	0.250561	C	-0.01888	1.810632	0.250992
C	1.38883	1.993548	0.286423	C	-1.38883	1.993524	0.28649	C	-1.3889	1.99351	0.286951
C	5.710783	-0.41405	0.055366	C	-5.7108	-0.41402	0.05529	C	-5.71086	-0.41411	0.054911
C	6.291709	0.498477	-1.04412	C	-6.29167	0.498509	-1.04422	C	-6.29154	0.49874	-1.04443
C	6.13585	0.063442	1.45903	C	-6.13592	0.06348	1.458936	C	-6.13616	0.062987	1.458634
C	-0.05796	-2.53733	-0.82694	C	0.057953	-2.53739	-0.8268	C	0.058097	-2.53729	-0.82672
C	2.0976	3.295521	0.54513	C	-2.09759	3.2955	0.545209	C	-2.09773	3.2954	0.545804
C	-1.63636	-0.12474	0.088937	C	1.63637	-0.12476	0.088971	C	1.636384	-0.1247	0.089342
C	-2.87209	0.489513	-0.02245	C	2.872092	0.48952	-0.02244	C	2.872084	0.489538	-0.02234
C	-4.04173	-0.3096	0.21435	C	4.041743	-0.30958	0.214326	C	4.041811	-0.30955	0.214214
N	-5.24727	0.224875	0.150946	N	5.247283	0.224903	0.150894	N	5.247306	0.224952	0.150423
C	-5.56889	1.604086	-0.20381	C	5.56888	1.604111	-0.20389	C	5.568823	1.60408	-0.20461
C	-4.44792	2.182591	-1.06402	C	4.447874	2.1826	-1.06407	C	4.447574	2.18255	-1.06451
C	-3.08575	1.951886	-0.39252	C	3.085727	1.951893	-0.39253	C	3.085618	1.951892	-0.39256
C	-4.15932	-1.87376	0.573027	C	4.159369	-1.87374	0.572998	C	4.15956	-1.87372	0.573269
O	-5.3518	-2.20877	0.767361	O	3.104203	-2.53772	0.598554	O	3.104344	-2.53758	0.599507
O	-3.10414	-2.53772	0.598596	O	5.351855	-2.20871	0.767331	O	5.352115	-2.20861	0.767179
H	4.428628	-2.63121	-0.45461	H	-4.42866	-2.6312	-0.45464	H	-4.42862	-2.63116	-0.4554
H	2.307501	-3.33713	-0.8035	H	-2.30753	-3.33715	-0.80344	H	-2.30739	-3.33706	-0.80391
H	3.930459	1.494117	0.268213	H	-3.93046	1.494115	0.268229	H	-3.93054	1.493987	0.268571
H	-0.69781	2.598896	0.444971	H	0.697812	2.598861	0.445047	H	0.697781	2.598869	0.445495
H	6.146402	-1.40919	-0.09078	H	-6.14643	-1.40916	-0.09087	H	-6.14643	-1.40922	-0.0916
H	5.931548	1.52898	-0.94798	H	-6.01966	0.141098	-2.04284	H	-5.93136	1.529208	-0.94793
H	6.019734	0.141069	-2.04275	H	-5.93151	1.529008	-0.94807	H	-7.38502	0.525271	-0.97897
H	7.385175	0.52498	-0.97845	H	-7.38514	0.525021	-0.9786	H	-6.01938	0.141615	-2.04312
H	5.749191	-0.60024	2.239299	H	-5.77306	1.075509	1.670218	H	-7.22879	0.082146	1.538768
H	5.772983	1.07547	1.670305	H	-5.74929	-0.60019	2.239223	H	-5.77332	1.074956	1.670264
H	7.228466	0.082604	1.539346	H	-7.22854	0.082642	1.539212	H	-5.74964	-0.60091	2.238789
H	0.294362	-3.44613	-1.3197	H	0.632029	-2.84645	0.051999	H	0.761362	-2.04223	-1.49985

H	-0.76128	-2.04231	-1.49998	H	0.761268	-2.04242	-1.49988	H	-0.29409	-3.44621	-1.31935
H	-0.63204	-2.84645	0.051834	H	-0.29437	-3.44623	-1.31949	H	0.632362	-2.84622	0.051987
H	2.752389	3.581634	-0.28725	H	-2.75237	3.58163	-0.28717	H	-1.37409	4.103913	0.683233
H	1.373864	4.103995	0.682289	H	-1.37385	4.103967	0.682387	H	-2.75244	3.581675	-0.28659
H	2.717932	3.256001	1.449044	H	-2.71793	3.255971	1.449116	H	-2.71823	3.255724	1.449607
H	-1.72149	-1.18379	0.293476	H	1.721524	-1.18381	0.293509	H	1.72153	-1.18378	0.293788
H	-5.96461	-0.48571	0.37559	H	5.964623	-0.48571	0.375518	H	5.964724	-0.48568	0.374845
H	-5.70197	2.192368	0.71431	H	6.525977	1.604924	-0.73454	H	5.702273	2.1925	0.713378
H	-6.526	1.604899	-0.73443	H	5.701994	2.192406	0.714215	H	6.525761	1.604841	-0.73555
H	-4.46298	1.695508	-2.04583	H	4.621362	3.251352	-1.22599	H	4.462312	1.695408	-2.0463
H	-4.62142	3.251344	-1.22592	H	4.462904	1.69551	-2.04588	H	4.621079	3.251277	-1.22655
H	-3.01717	2.585731	0.503327	H	3.017179	2.585741	0.503321	H	3.017322	2.585781	0.50329
H	-2.29179	2.280084	-1.06965	H	2.29175	2.280091	-1.06963	H	2.291426	2.280086	-1.06941

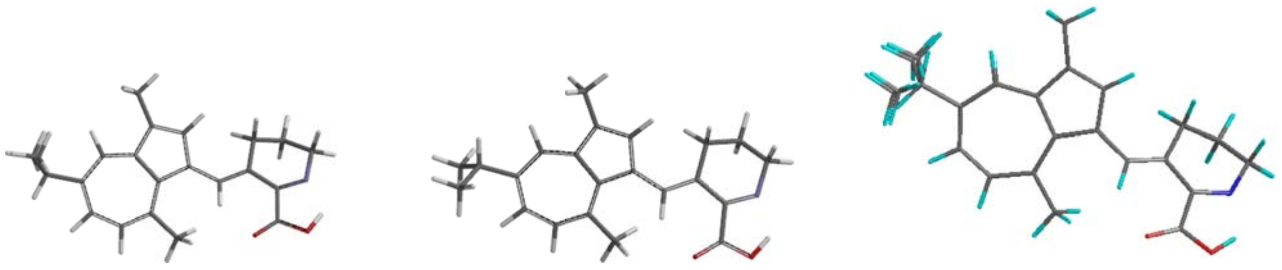

1b1 (52.1%) **1b2 (47.8%)**

Figure S3 Stable conformers of compound **1** with non-ionized salt structure.

Table S3. Important thermodynamic parameters (a.u.) of the optimized compound **1** with non-ionized structure at B3LYP/6-31g(d,p) level in the gas phase

conformations	E+ZPE	G
**1b1**	-1058.188364	-1058.242606
**1b2**	-1058.188287	-1058.242523

Table S4. Optimized Z-Matrixes of compound **1** with non-ionized structure in the Gas Phase (Å) at B3LYP/6-31g(d,p) level.

**1b1**				**1b2**			
C	4.188305	-0.58631	0.117631	C	4.19695	-0.18532	0.041813
C	3.669949	-1.84244	0.440426	C	3.763543	-1.47531	0.372748
C	2.354454	-2.27409	0.616216	C	2.484066	-1.98997	0.560917
C	1.107555	-1.63368	0.478571	C	1.193786	-1.42943	0.43612
C	0.918204	-0.29657	0.07036	C	0.914348	-0.1124	0.025878
C	2.01249	0.701118	-0.14167	C	1.937935	0.953637	-0.20239
C	3.393249	0.544388	-0.11373	C	3.329699	0.886932	-0.18661
C	-0.31055	0.411513	-0.12928	C	-0.36324	0.514193	-0.16107
C	0.031429	1.765812	-0.42048	C	-0.1128	1.883495	-0.45922
C	1.402554	1.955063	-0.43369	C	1.246102	2.161255	-0.48976
C	5.708742	-0.43251	0.034023	C	5.702656	0.072016	-0.05661

C	6.173519	-0.00597	-1.37144	C	6.419837	-0.15295	1.288083
C	6.254236	0.522726	1.112729	C	6.358316	-0.75281	-1.18042
C	-0.07934	-2.51517	0.822563	C	0.072111	-2.38532	0.799062
C	2.109886	3.244497	-0.73561	C	1.862788	3.494274	-0.80189
C	-1.64362	-0.14391	-0.18273	C	-1.65717	-0.12828	-0.19748
C	-2.84465	0.481376	0.015678	C	-2.89342	0.416991	0.019875
C	-4.10586	-0.23638	-0.18387	C	-4.1081	-0.38138	-0.15989
N	-5.29166	0.262277	-0.02869	N	-5.32127	0.039131	0.013914
C	-5.47084	1.631147	0.433763	C	-5.58194	1.394079	0.478397
C	-4.28525	2.149031	1.244571	C	-4.42005	1.989825	1.269974
C	-2.98711	1.926438	0.461276	C	-3.12164	1.850053	0.468033
C	-4.19576	-1.71607	-0.62779	C	-4.10804	-1.86467	-0.60066
O	-3.25913	-2.46743	-0.81098	O	-3.12736	-2.55393	-0.79697
O	-5.45977	-2.1075	-0.78534	O	-5.3461	-2.33811	-0.73856
H	4.414392	-2.61963	0.608141	H	4.554727	-2.20445	0.536903
H	2.281814	-3.31557	0.919644	H	2.47942	-3.03347	0.866117
H	3.943452	1.459435	-0.32382	H	3.82104	1.833992	-0.40479
H	-0.68538	2.537992	-0.66713	H	-0.87995	2.608109	-0.69913
H	6.139416	-1.42222	0.230614	H	5.827929	1.13045	-0.31704
H	7.267231	0.026339	-1.41946	H	5.979871	0.460716	2.080095
H	5.819207	-0.70427	-2.13573	H	6.360652	-1.19986	1.605138
H	5.803102	0.99127	-1.63259	H	7.4807	0.1064	1.205152
H	5.884379	1.543932	0.969364	H	6.297069	-1.82774	-0.97847
H	7.348202	0.560269	1.074208	H	5.874143	-0.56636	-2.14379
H	5.959196	0.198846	2.115454	H	7.418696	-0.49575	-1.27588
H	-0.80726	-1.98772	1.442329	H	-0.43971	-2.77895	-0.08522
H	0.250619	-3.40257	1.36674	H	0.464768	-3.24012	1.353733
H	-0.61241	-2.86018	-0.06925	H	-0.68773	-1.90082	1.415178
H	1.38649	4.038291	-0.94118	H	2.514301	3.453736	-1.68355
H	2.763975	3.163684	-1.61268	H	1.086314	4.237115	-1.00442
H	2.734401	3.580927	0.101445	H	2.469788	3.875044	0.028979
H	-1.71794	-1.18559	-0.45116	H	-1.66664	-1.1726	-0.46641
H	-5.63987	2.266643	-0.44903	H	-6.50085	1.370518	1.075056
H	-6.39901	1.667507	1.015241	H	-5.80709	2.01523	-0.40214
H	-4.41917	3.210032	1.481337	H	-4.32468	1.455348	2.22322
H	-4.23908	1.608063	2.197762	H	-4.61902	3.040419	1.507384
H	-2.98604	2.593409	-0.41412	H	-3.17539	2.515386	-0.40693
H	-2.12237	2.211053	1.067978	H	-2.26763	2.189034	1.061934
H	-5.98417	-1.29643	-0.54776	H	-5.91854	-1.56254	-0.49373

Table S5. Calculated ^{13}C NMR chemical shifts for the conformers of **1** with respective inner salt (**1a1**-**1a9**) and non-ionized structure (**1b1** and **1b2**).

No.	**1a1**	**1a2**	**1a3 1**	**a4 1**	**a5 1**	**a6 1**	**a7 1**	**a8**	**1a9**	**1b1**	**1b2**
1	135.8	135.8	135.3	135.3	135.3	135.3	135.3	135.3	135.3	131.9	132.1
2	146.1	146.1	145.3	145.3	145.3	145.3	145.3	145.3	145.3	144.2	144.8
3	130.3	130.3	130.6	130.6	130.6	130.6	130.6	130.6	130.6	129.1	128.6
4	161.5	161.5	161.0	161.0	161.0	161.0	161.0	161.0	161.0	158.4	158.6
5	140.5	140.5	140.9	140.9	140.9	140.9	140.9	140.9	140.9	135.6	135.0
6	143.2	143.2	146.8	146.8	146.8	146.8	146.8	146.8	146.8	144.7	141.4
7	157.6	157.6	157.6	157.6	157.6	157.6	157.6	157.6	157.6	152.4	151.8
8	143.2	143.2	140.4	140.4	140.4	140.4	140.4	140.4	140.4	139.4	142.7
9	151.5	151.5	151.8	151.8	151.8	151.8	151.8	151.8	151.8	148.1	147.6

10	150.6	150.6	150.7	150.7	150.7	150.7	150.7	150.7	150.7	146.1	146.2
11	46.5	46.5	46.0	46.0	46.0	46.0	46.0	46.0	46.0	45.9	46.1
12	25.9	25.9	26.0	26.0	26.1	26.0	26.1	26.1	26.1	25.9	25.9
13	25.4	25.4	26.1	26.1	26.0	26.1	26.0	26.0	26.0	26.2	25.6
14	15.1	15.1	15.3	15.3	15.3	15.3	15.3	15.3	15.3	14.8	14.8
15	32.9	32.9	32.8	32.8	32.8	32.8	32.8	32.8	32.8	32.1	32.1
16	160.1	160.1	160.7	160.7	160.7	160.7	160.7	160.7	160.7	145.8	144.6
2'	171.4	171.4	171.4	171.4	171.4	171.4	171.4	171.4	171.4	163.1	163.1
3'	124.0	124.0	124.2	124.2	124.2	124.2	124.2	124.2	124.2	126.9	126.8
4'	28.5	28.5	28.7	28.7	28.7	28.7	28.7	28.7	28.7	29.7	29.3
5'	24.4	24.4	24.4	24.4	24.4	24.4	24.4	24.4	24.5	25.5	25.6
6'	45.4	45.4	45.3	45.3	45.3	45.3	45.3	45.3	45.3	53.5	53.4
7'	166.0	166.0	165.9	165.9	165.9	165.9	165.9	165.9	165.9	172.2	172.4

2. ECD calculations for 2 to determine the absolute configurations.

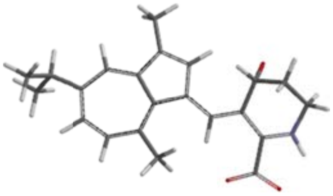

2a1 (25.6%)

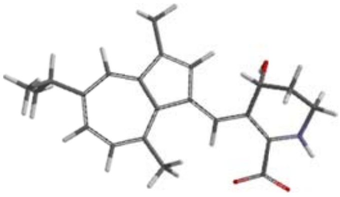

2a2 (25.6%)

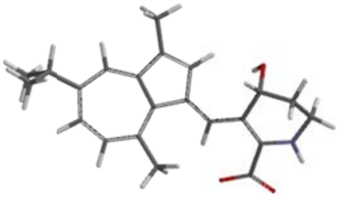

2a3 (25.5%)

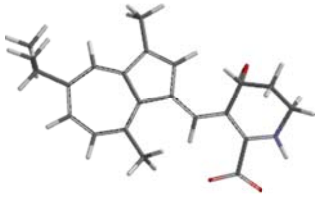

2a4 (23.1%)

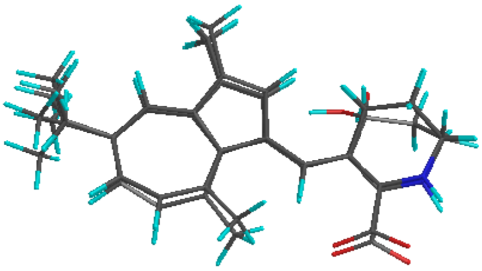

overlaid image

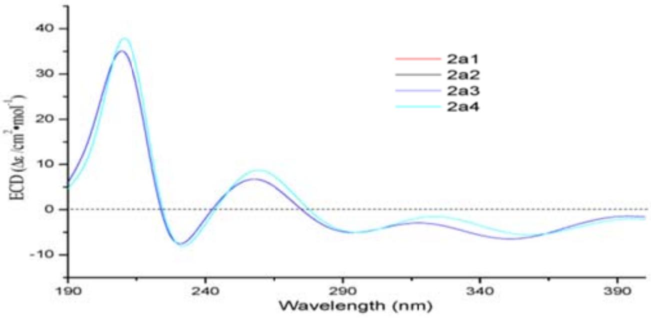

Figure S4. Structural candidates of compound **2** with 4'-*R* absolute configuration and the overlaid ECD calculated on Gaussian program.

Table S6. Important thermodynamic parameters (a.u.) of the optimized **2** with 4'-R absolute configuration at B3LYP/6-31G(d,p) level in the gas phase

conformations	E+ZPE	G
**2a1**	-1133.468941	-1133.524646
**2a2**	-1133.468941	-1133.524647
**2a3**	-1133.468940	-1133.524644
**2a4**	-1133.468923	-1133.524551

Table S7. Optimized *Z*-Matrixes of compound **2** with 4'-*R* absolute configuration in the Gas Phase (Å) at B3LYP/DGDZVP level.

	**2a1 2a2**						
C	-4.33893	-0.15106	-0.00081	C	-4.33886	-0.15114	-0.00093
C	-3.95644	-1.48386	0.224366	C	-3.95635	-1.48391	0.224447
C	-2.70035	-2.04851	0.434549	C	-2.70026	-2.04844	0.434956
C	-1.38543	-1.52108	0.426136	C	-1.38536	-1.52093	0.426511
C	-1.05672	-0.18172	0.128945	C	-1.05673	-0.1816	0.12917
C	-2.03301	0.926519	-0.02612	C	-2.03302	0.92663	-0.02589
C	-3.43091	0.909018	-0.07745	C	-3.43091	0.909021	-0.07738
C	0.253348	0.42424	0.032609	C	0.253328	0.424369	0.032891
C	0.049941	1.835133	-0.13213	C	0.049916	1.835268	-0.13185
C	-1.29789	2.145538	-0.1686	C	-1.29793	2.145666	-0.16824
C	-5.82941	0.16009	-0.15292	C	-5.82932	0.159969	-0.1534
C	-6.62905	-0.17783	1.121892	C	-6.43739	-0.52702	-1.39325
C	-6.43767	-0.52671	-1.39281	C	-6.62921	-0.17777	1.121318
C	-0.31702	-2.53989	0.779721	C	-0.31692	-2.53967	0.780262
C	-1.87647	3.519893	-0.37064	C	-1.87653	3.520033	-0.37014
C	1.501749	-0.26122	-0.04319	C	1.501709	-0.2611	-0.04307
C	2.801283	0.219207	0.072773	C	2.801282	0.219239	0.072748
C	3.892615	-0.68993	-0.14217	C	3.892522	-0.69	-0.14234
N	5.141518	-0.28053	-0.03515	N	5.141465	-0.28065	-0.03554
C	5.580062	1.069073	0.31141	C	5.580083	1.068964	0.310775
C	4.500755	1.752149	1.144368	C	4.501029	1.752039	1.144082
C	3.141696	1.650244	0.446408	C	3.141842	1.650236	0.446364
C	3.85375	-2.25175	-0.51815	C	3.853548	-2.25185	-0.51811
O	3.15923	2.532414	-0.70674	O	3.159216	2.532449	-0.70677
O	5.007869	-2.71579	-0.66746	O	2.731032	-2.79026	-0.6024
O	2.731282	-2.79023	-0.60235	O	5.007614	-2.71604	-0.66731
H	-4.77212	-2.20346	0.274133	H	-4.772	-2.20352	0.274167
H	-2.73978	-3.11496	0.645317	H	-2.73966	-3.11484	0.645967
H	-3.88148	1.890027	-0.22118	H	-3.88154	1.890001	-0.22114
H	0.842375	2.55826	-0.289	H	0.842345	2.558405	-0.28868
H	-5.91613	1.24164	-0.30945	H	-5.91601	1.241492	-0.31011
H	-6.21638	0.332932	1.998155	H	-7.48144	-0.22118	-1.52303
H	-6.62673	-1.25365	1.329474	H	-6.42355	-1.61863	-1.30002
H	-7.67367	0.13243	1.00822	H	-5.88985	-0.26237	-2.30373
H	-5.89033	-0.26176	-2.30332	H	-6.21667	0.333038	1.997615
H	-6.42361	-1.61834	-1.29982	H	-6.62699	-1.25358	1.328967
H	-7.48179	-0.22101	-1.52232	H	-7.67379	0.132545	1.007441
H	-0.76943	-3.41998	1.242097	H	0.243474	-2.88571	-0.09382
H	0.415547	-2.13361	1.480206	H	0.415885	-2.1332	1.480364
H	0.243686	-2.88553	-0.09432	H	-0.76931	-3.41951	1.243132
H	-1.07716	4.261744	-0.4501	H	-2.47657	3.584948	-1.28617
H	-2.52177	3.821643	0.463484	H	-2.52173	3.821729	0.464079
H	-2.47641	3.58475	-1.28675	H	-1.07722	4.261887	-0.44964
H	1.481457	-1.32132	-0.26009	H	1.481319	-1.32119	-0.26005
H	5.792866	-1.05562	-0.24408	H	5.792797	-1.05575	-0.24443
H	6.525082	0.992826	0.856492	H	6.525336	0.992824	0.855462
H	5.767949	1.636466	-0.60871	H	5.767535	1.636372	-0.60943
H	4.752883	2.806624	1.289307	H	4.434809	1.279676	2.13137
H	4.434375	1.279914	2.131705	H	4.753266	2.806474	1.289084
H	2.376413	2.030348	1.131824	H	2.376698	2.030391	1.131911
H	2.941034	2.006848	-1.49218	H	2.940834	2.006921	-1.49219
	**2a3 2a4**						

C	-4.33891	-0.15103	-0.00079	C	-4.33885	0.564654	-0.05067
C	-3.95643	-1.48385	0.224279	C	-3.86858	1.865922	-0.27061
C	-2.70035	-2.0485	0.434528	C	-2.57098	2.351382	-0.45129
C	-1.38544	-1.52104	0.426292	C	-1.29507	1.744575	-0.41388
C	-1.0567	-0.18169	0.129121	C	-1.05863	0.381384	-0.12398
C	-2.03299	0.926559	-0.02589	C	-2.10901	-0.66082	0.01374
C	-3.43088	0.909058	-0.07727	C	-3.49971	-0.55359	0.04458
C	0.253354	0.42425	0.03283	C	0.204572	-0.31053	-0.01818
C	0.049963	1.835164	-0.13182	C	-0.09312	-1.70797	0.13984
C	-1.29786	2.145591	-0.16823	C	-1.45608	-1.92842	0.162811
C	-5.82937	0.160121	-0.15306	C	-5.84995	0.355866	0.070793
C	-6.43731	-0.52629	-1.39333	C	-6.25023	-0.18677	1.457818
C	-6.6293	-0.17827	1.121441	C	-6.40832	-0.53284	-1.05936
C	-0.31708	-2.53989	0.779967	C	-0.15435	2.696269	-0.72662
C	-1.87644	3.519955	-0.37021	C	-2.12767	-3.26133	0.353907
C	1.501743	-0.26121	-0.04308	C	1.498692	0.285216	0.05885
C	2.801295	0.219181	0.072787	C	2.759647	-0.28649	-0.06863
C	3.892582	-0.68996	-0.14231	C	3.915137	0.539654	0.147909
N	5.141506	-0.28052	-0.03565	N	5.130644	0.043473	0.025785
C	5.580114	1.069062	0.310901	C	5.469277	-1.32922	-0.34124
C	4.500979	1.752071	1.144139	C	4.337282	-1.9231	-1.1727
C	3.141793	1.650198	0.446436	C	2.995007	-1.73402	-0.4599
C	3.853615	-2.2518	-0.51805	C	3.990883	2.095194	0.543332
O	3.159103	2.532448	-0.70666	O	2.95983	-2.62714	0.684285
O	2.7311	-2.79027	-0.60186	O	2.910596	2.712087	0.638661
O	5.007678	-2.7159	-0.6676	O	5.176145	2.472411	0.693275
H	-4.7721	-2.20346	0.27391	H	-4.63889	2.633441	-0.34042
H	-2.73979	-3.11496	0.645228	H	-2.54171	3.418278	-0.661
H	-3.88145	1.890081	-0.22094	H	-4.00971	-1.5052	0.181456
H	0.842383	2.558319	-0.28862	H	0.649549	-2.48136	0.299812
H	-5.9161	1.241721	-0.30924	H	-6.31726	1.341604	-0.03577
H	-6.42317	-1.61794	-1.30072	H	-5.88177	0.460481	2.260369
H	-7.48143	-0.22063	-1.52296	H	-5.85182	-1.19287	1.629801
H	-5.88979	-0.26097	-2.30363	H	-7.34102	-0.24587	1.54224
H	-7.67389	0.132019	1.007636	H	-6.01611	-1.55455	-1.00407
H	-6.21684	0.332191	1.99798	H	-6.15439	-0.12983	-2.04529
H	-6.62702	-1.25416	1.328653	H	-7.49987	-0.59573	-0.98865
H	0.415631	-2.13357	1.480267	H	0.556528	2.260712	-1.43219
H	-0.76953	-3.41983	1.242563	H	0.41867	2.975414	0.162731
H	0.243467	-2.88573	-0.0941	H	-0.53827	3.61916	-1.16647
H	-2.52217	3.821453	0.463668	H	-2.74641	-3.28649	1.259442
H	-1.07714	4.261887	-0.44908	H	-1.38057	-4.05427	0.446735
H	-2.47592	3.584987	-1.28661	H	-2.77654	-3.52036	-0.49183
H	1.48142	-1.32133	-0.25992	H	1.555376	1.342029	0.28462
H	5.792848	-1.05558	-0.24466	H	5.837279	0.766905	0.240681
H	6.525256	0.992774	0.855766	H	5.623366	-1.92054	0.570064
H	5.767805	1.636531	-0.60921	H	6.413099	-1.31339	-0.89346
H	4.434836	1.279785	2.131467	H	4.512606	-2.99081	-1.33307
H	4.753115	2.806546	1.28907	H	4.296037	-1.43448	-2.15342
H	2.376619	2.030267	1.131992	H	2.198635	-2.05257	-1.14093
H	2.941057	2.006884	-1.49214	H	2.806448	-2.09304	1.479257

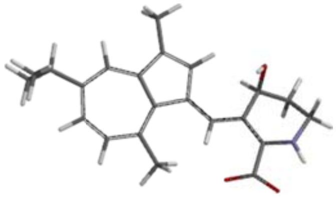

2b1 (34.9%)

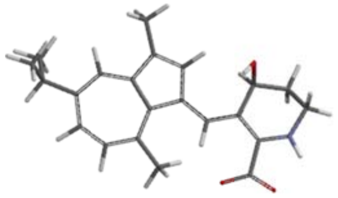

2b2 (31.6%)

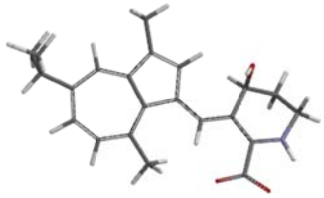

2b3 (31.5%)

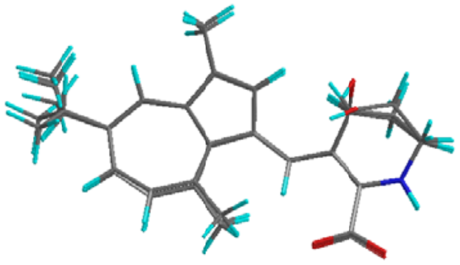

Overlaid image

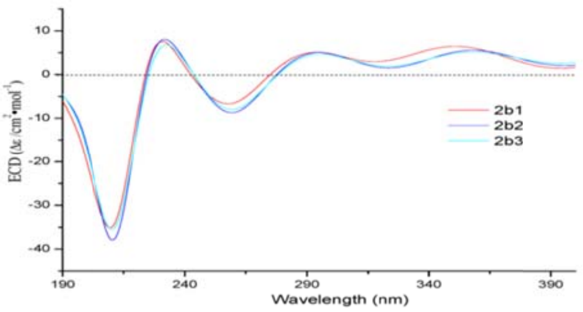

Figure S5. Structural candidates of compound **2** with 4'-*S* absolute configuration and the overlaid ECD calculated on Gaussian program.

Table S8. Important thermodynamic parameters (a.u.) of the optimized **2** with 4'-*S* absolute configuration at B3LYP/6-31G(d,p) level in the gas phase

conformations	E+ZPE	G
**2b1**	-1133.468942	-1133.524646
**2b2**	-1133.468924	-1133.524552
**2b3**	-1133.468923	-1133.524551

Table S9. Optimized Z-Matrixes of compound **2** with 4'-*S* absolute configuration in the Gas Phase (Å) at B3LYP/DGDZVP level.

**2b1**				**2b2**				**2b3**			
C	4.338869	-0.15105	-0.00084	C	4.338882	0.564657	-0.05074	C	4.338836	0.564667	-0.0505
C	3.956375	-1.48387	0.224297	C	3.868593	1.865938	-0.2706	C	3.868528	1.865956	-0.27031
C	2.700306	-2.04847	0.434691	C	2.570977	2.351418	-0.45114	C	2.570937	2.351376	-0.45119
C	1.385407	-1.52096	0.426433	C	1.295058	1.744614	-0.41366	C	1.295047	1.744519	-0.41397
C	1.056724	-0.18163	0.129191	C	1.058632	0.381404	-0.12382	C	1.05861	0.381324	-0.12403
C	2.032994	0.926615	-0.02593	C	2.109026	-0.66079	0.013873	C	2.10901	-0.66085	0.013646
C	3.430896	0.909076	-0.07728	C	3.499731	-0.55357	0.044583	C	3.499708	-0.55361	0.044492
C	-0.25334	0.424304	0.032834	C	-0.20457	-0.31053	-0.01803	C	-0.20458	-0.3106	-0.01828
C	-0.04996	1.835199	-0.13193	C	0.093151	-1.70797	0.140004	C	0.093136	-1.70806	0.139694
C	1.297872	2.145625	-0.16836	C	1.456115	-1.9284	0.162955	C	1.456096	-1.92849	0.162636
C	5.82934	0.16004	-0.15315	C	5.849983	0.355857	0.070553	C	5.849918	0.355912	0.071008
C	6.437307	-0.5265	-1.39332	C	6.408188	-0.53299	-1.05957	C	6.40836	-0.53259	-1.05928
C	6.629244	-0.17823	1.121411	C	6.250454	-0.18664	1.457587	C	6.250179	-0.18695	1.457953
C	0.317008	-2.53972	0.780232	C	0.154326	2.69634	-0.72628	C	0.154351	2.696134	-0.72707
C	1.876443	3.520008	-0.37024	C	2.127729	-3.2613	0.353952	C	2.127732	-3.26139	0.353565
C	-1.50172	-0.26117	-0.04309	C	-1.49871	0.2852	0.058925	C	-1.49867	0.285169	0.058788
C	-2.80127	0.219202	0.072819	C	-2.75966	-0.28649	-0.06866	C	-2.75967	-0.28649	-0.06865
C	-3.89253	-0.68997	-0.14236	C	-3.91517	0.539632	0.147851	C	-3.91509	0.5397	0.14797

N	-5.14146	-0.28059	-0.0356	N	-5.13067	0.043401	0.025764	N	-5.13065	0.043539	0.026059
C	-5.58009	1.06896	0.310963	C	-5.46929	-1.32928	-0.34132	C	-5.4694	-1.32908	-0.34109
C	-4.50097	1.752001	1.144207	C	-4.33724	-1.92319	-1.17266	C	-4.33745	-1.92304	-1.17255
C	-3.1418	1.650203	0.446461	C	-2.99499	-1.73404	-0.45986	C	-2.99512	-1.73402	-0.45986
C	-3.85357	-2.25181	-0.51819	C	-3.99097	2.09517	0.543166	C	-3.99072	2.095211	0.54333
O	-3.15918	2.532432	-0.70665	O	-2.95985	-2.62709	0.684385	O	-2.95989	-2.62712	0.684324
O	-5.00763	-2.7159	-0.6677	O	-2.91069	2.712059	0.638671	O	-2.91038	2.712066	0.638531
O	-2.73106	-2.79028	-0.60208	O	-5.17626	2.472446	0.69281	O	-5.17594	2.47252	0.693392
H	4.772052	-2.20348	0.273916	H	4.638902	2.633453	-0.34046	H	4.638821	2.63351	-0.33992
H	2.739724	-3.11491	0.645507	H	2.541697	3.418327	-0.66078	H	2.541642	3.418279	-0.66087
H	3.881499	1.890082	-0.22094	H	4.009736	-1.50519	0.181377	H	4.009719	-1.50524	0.181196
H	-0.84239	2.558322	-0.2888	H	-0.64949	-2.48139	0.299915	H	-0.64954	-2.48143	0.299685
H	5.916113	1.241621	-0.30944	H	6.317302	1.341573	-0.03618	H	6.317208	1.34168	-0.03537
H	5.889828	-0.26126	-2.30367	H	6.154134	-0.1301	-2.04551	H	6.154474	-0.12942	-2.04515
H	6.423156	-1.61815	-1.30062	H	6.015972	-1.55469	-1.0041	H	7.499903	-0.59549	-0.98852
H	7.481437	-0.22087	-1.52295	H	7.499744	-0.59589	-0.989	H	6.016152	-1.55431	-1.00417
H	6.626985	-1.25411	1.328703	H	5.852047	-1.1927	1.629735	H	5.851824	-1.19311	1.629731
H	6.216753	0.332295	1.997898	H	5.882124	0.460714	2.260119	H	7.340964	-0.24599	1.542409
H	7.67383	0.132077	1.007602	H	7.341251	-0.24575	1.541855	H	5.881639	0.460123	2.260608
H	-0.24352	-2.88566	-0.09381	H	-0.41876	2.975317	0.163068	H	-0.55646	2.260327	-1.43256
H	-0.4157	-2.13334	1.48048	H	-0.55649	2.260867	-1.43197	H	0.538338	3.618865	-1.16719
H	0.769447	-3.41962	1.242942	H	0.538244	3.619312	-1.16596	H	-0.41874	2.975581	0.162136
H	2.5209	3.822057	0.464432	H	2.775999	-3.5206	-0.49216	H	2.74728	-3.28634	1.258546
H	1.077094	4.261723	-0.45065	H	1.380633	-4.05417	0.447491	H	2.775806	-3.52074	-0.49268
H	2.477242	3.584722	-1.28579	H	2.747073	-3.28629	1.259073	H	1.380643	-4.05424	0.447317
H	-1.48136	-1.32127	-0.26005	H	-1.5554	1.34203	0.28463	H	-1.55534	1.342007	0.284484
H	-5.7928	-1.05566	-0.24458	H	-5.83731	0.76686	0.24056	H	-5.83721	0.766981	0.241161
H	-5.76776	1.636461	-0.60914	H	-5.62356	-1.92059	0.569959	H	-5.62371	-1.92044	0.570153
H	-6.52525	0.992691	0.855808	H	-6.41304	-1.3134	-0.89369	H	-6.41317	-1.3131	-0.89342
H	-4.75318	2.80644	1.289236	H	-4.51253	-2.99091	-1.33297	H	-4.29623	-1.43444	-2.15328
H	-4.43474	1.279634	2.131492	H	-4.29596	-1.43464	-2.15342	H	-4.51284	-2.99073	-1.33288
H	-2.37663	2.030316	1.132003	H	-2.19861	-2.0526	-1.14089	H	-2.19882	-2.05261	-1.14096
H	-2.94074	2.006948	-1.49207	H	-2.80611	-2.09301	1.479294	H	-2.80657	-2.09301	1.479298

3. ECD and OR calculations for 4 to determine the absolute configurations.

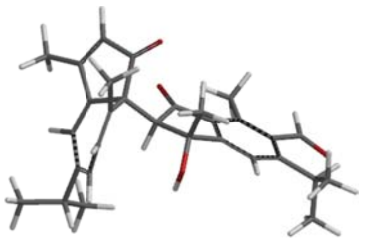

4a1 (96.0%, $[\alpha]_D = 276.44$)

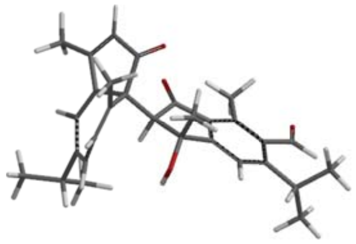

4a2 (3.9%)

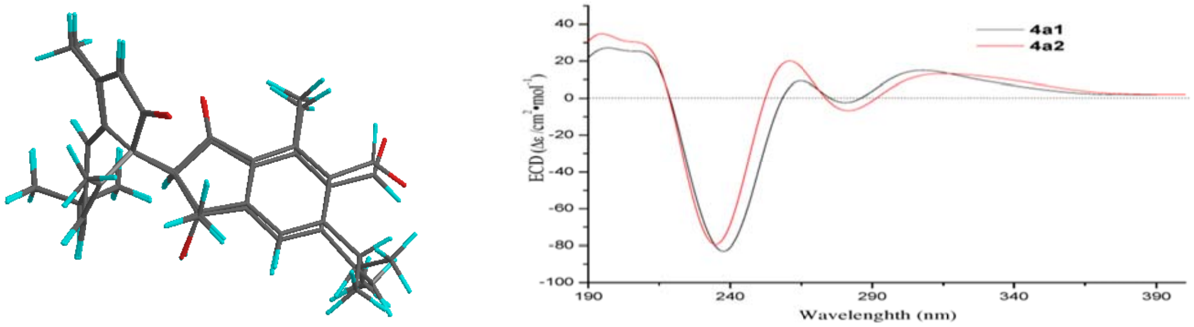

Figure S6. Structural candidates of compound **4a** with 10*S*,1'*R*,2'*R* absolute configuration, and the optical rotation and overlaid ECD calculated on Gaussian program.

Table S10. Important thermodynamic parameters (a.u.) of the optimized **2** with 10*S*,1'*R*,2'*R* absolute configuration at B3LYP/6-31G(d,p) level in the gas phase

conformations	E+ZPE	G
**4a1**	-1464.009683	-1464.073696
**4a2**	-1464.005352	-1464.070662

Table S11. Optimized Z-Matrixes of compound **4** with 10*S*,1'*R*,2'*R* absolute configuration in the Gas Phase (Å) at B3LYP/6-31g(d,p) level.

**4a1**				**4a2**			
C	3.146499	1.946038	0.092825	C	-3.21375	1.8782	-0.30885
C	2.898552	1.825494	1.439365	C	-3.01979	1.579911	-1.63615
C	2.941951	0.62662	2.233995	C	-3.06389	0.282883	-2.25843
C	2.693673	-0.65461	1.854718	C	-2.76519	-0.92914	-1.7213
C	2.043395	-0.95305	0.497709	C	-2.05411	-1.02653	-0.36512
C	3.079096	-0.48464	-0.51719	C	-3.05945	-0.4448	0.622084
C	3.457498	0.805828	-0.73257	C	-3.46266	0.854888	0.676241
C	1.989214	-2.48656	0.200866	C	-1.94783	-2.50331	0.135097
C	3.027923	-2.7877	-0.78398	C	-2.93753	-2.68796	1.196382
C	3.6186	-1.64868	-1.22362	C	-3.53923	-1.51198	1.503045
C	3.272086	3.33907	-0.52287	C	-3.34134	3.339242	0.11993
C	3.083406	-1.80693	2.741207	C	-3.15496	-2.19929	-2.42921
C	4.676672	-1.55533	-2.27819	C	-4.55486	-1.29573	2.581019
O	1.210486	-3.2808	0.705932	O	-1.17104	-3.34374	-0.29179
C	-0.11112	-0.79432	-0.94308	C	0.150225	-0.61552	0.947193
C	0.652462	-0.26845	0.287864	C	-0.67569	-0.28998	-0.3131
C	-0.43159	-0.14198	1.421161	C	0.359087	-0.30312	-1.4967
C	-1.72529	-0.12348	0.611099	C	1.678818	-0.12437	-0.75069
C	-1.55797	-0.5578	-0.70525	C	1.579435	-0.35851	0.621189
C	-2.97044	0.264403	1.083577	C	2.890358	0.231017	-1.32455
C	-4.09267	0.20695	0.249938	C	4.045368	0.324809	-0.54099
C	-3.92567	-0.26307	-1.09185	C	3.950097	0.057349	0.861932
C	-2.65244	-0.65831	-1.58169	C	2.705221	-0.2827	1.464165
O	-0.30188	1.053974	2.194268	O	0.161126	0.759111	-2.43368
C	-0.47592	-1.27308	2.452632	C	0.407833	-1.57328	-2.35051
C	-5.44402	0.655668	0.801664	C	5.320716	0.728912	-1.29731
O	0.421371	-1.26304	-1.93618	O	-0.33074	-0.96862	2.010361
C	-5.67735	2.148455	0.48477	C	6.373093	-0.3951	-1.41348
C	2.334963	3.525572	-1.72999	C	-2.34734	3.700952	1.238614
C	4.727653	3.679701	-0.89955	C	-4.78275	3.703962	0.526629
C	-5.61761	0.386953	2.306205	C	5.930656	2.074309	-0.85063
H	0.875762	0.773138	0.021372	H	-0.91727	0.773851	-0.18768
C	-2.43576	-1.1472	-3.00057	C	2.557282	-0.555	2.939981
C	-5.06988	-0.38296	-2.03068	C	5.176767	0.131555	1.694361
O	-6.16703	0.140551	-1.91812	O	5.232297	0.036098	2.909394
H	2.813005	2.75431	2.002381	H	-2.98063	2.424455	-2.32361
H	3.258145	0.776803	3.265767	H	-3.42526	0.284789	-3.28626

H	4.09473	0.994269	-1.59438	H	-4.06959	1.147281	1.5309
H	3.233145	-3.79535	-1.12477	H	-3.10202	-3.64359	1.679417
H	2.964047	4.055258	0.249679	H	-3.09013	3.949437	-0.75718
H	3.849761	-2.42008	2.246255	H	-3.88676	-2.7571	-1.828
H	2.24433	-2.47982	2.94205	H	-2.30575	-2.87471	-2.5698
H	3.500384	-1.4525	3.686977	H	-3.61437	-1.98549	-3.39734
H	4.317133	-0.96783	-3.13119	H	-4.17122	-0.59725	3.334015
H	4.961152	-2.54438	-2.64331	H	-4.80533	-2.23222	3.083668
H	5.572546	-1.0534	-1.89613	H	-5.47469	-0.85973	2.175753
H	-3.06042	0.614192	2.105165	H	2.933857	0.441937	-2.38879
H	-0.29553	1.806529	1.586848	H	0.120038	1.590904	-1.94211
H	0.377664	-1.18031	3.125738	H	-0.47538	-1.61134	-2.9896
H	-0.4439	-2.25031	1.971846	H	0.435725	-2.46906	-1.73119
H	-1.38722	-1.18028	3.049446	H	1.28946	-1.53799	-2.99642
H	-6.22005	0.099782	0.273258	H	4.976194	0.900124	-2.32384
H	-5.65722	2.32635	-0.5922	H	5.913616	-1.32206	-1.76959
H	-4.91429	2.774379	0.961413	H	6.877048	-0.61523	-0.46886
H	-6.65665	2.463589	0.860225	H	7.143459	-0.1024	-2.13483
H	1.289803	3.350174	-1.45477	H	-1.31435	3.504723	0.933134
H	2.584434	2.832176	-2.54035	H	-2.53954	3.119726	2.146938
H	2.412307	4.54348	-2.12695	H	-2.42583	4.762015	1.498883
H	5.10426	3.029304	-1.69661	H	-5.10035	3.163394	1.424906
H	5.393668	3.575081	-0.03783	H	-5.49074	3.470942	-0.27438
H	4.796778	4.710942	-1.26201	H	-4.85777	4.773941	0.748191
H	-4.98786	1.033557	2.92652	H	6.405422	2.029253	0.132653
H	-5.39216	-0.65301	2.562964	H	5.165931	2.856384	-0.8162
H	-6.6558	0.585467	2.590842	H	6.696441	2.38578	-1.5691
H	-2.81085	-0.43242	-3.73936	H	2.843598	0.321653	3.527473
H	-2.95235	-2.09769	-3.17626	H	3.240436	-1.34722	3.258142
H	-1.37474	-1.31073	-3.17951	H	1.532774	-0.83832	3.170361
H	-4.87319	-1.02192	-2.91151	H	6.112076	0.280224	1.130991

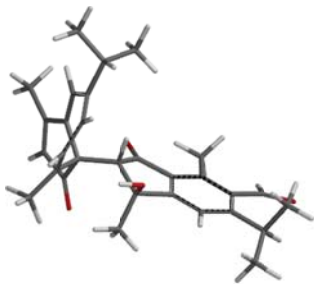

4b1 (34.3%, $[\alpha]_D$ = -278.5)

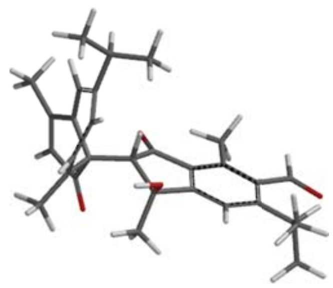

4b2 (15.7%, $[\alpha]_D$ = -513.1)

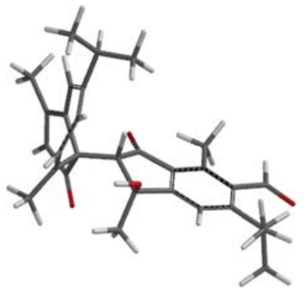

4b3 (15.6%, $[\alpha]_D$ = -513.5)

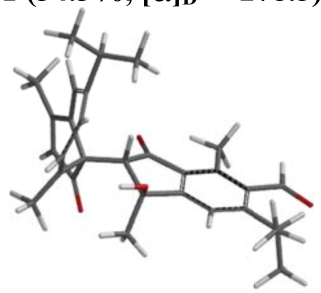

4b4 (15.6%, $[\alpha]_D$ = -513.3)

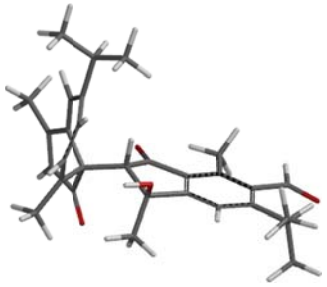

4b5 (14.3%, $[\alpha]_D$ = -457.7)

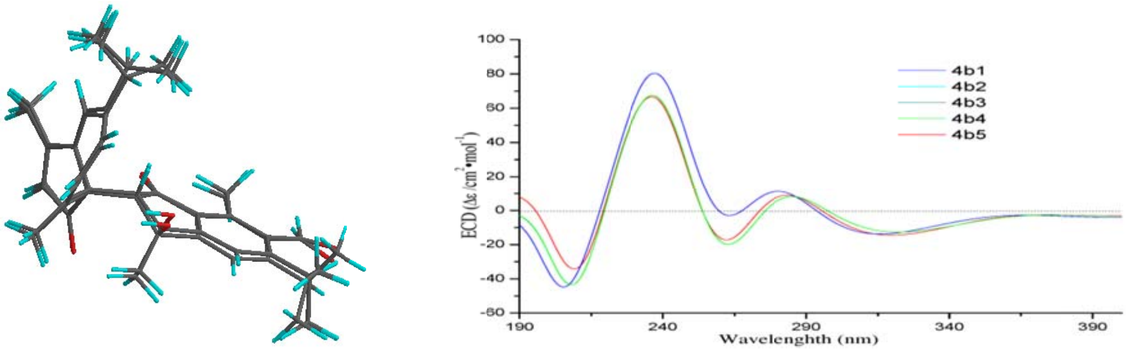

Figure S7. Structural candidates of compound **4b** with 10*R*,1'*S*,2'*S* absolute configuration, and the optical rotation and overlaid ECD calculated on Gaussian program.

Table S12. Important thermodynamic parameters (a.u.) of the optimized **4b** with 10R,1'S,2'S absolute configuration at B3LYP/6-31G(d,p) level in the gas phase

conformations	E+ZPE	G
**4b1**	-1464.011839	-1464.075662
**4b2**	-1464.010444	-1464.074926
**4b3**	-1464.010444	-1464.074922
**4b4**	-1464.010443	-1464.074921
**4b5**	-1464.010432	-1464.074838

Table S13. Optimized Z-Matrixes of compound **4** with 10R,1'S,2'S absolute configuration in the Gas Phase (Å) at B3LYP/6-31g(d,p) level.

**4b1**				**4b2**				**4b3**			
C	-3.11763	1.96959	0.111572	C	-3.24211	1.704692	-0.64394	C	-3.24239	1.704455	-0.64419
C	-2.87266	1.839355	1.456079	C	-3.0147	2.041176	0.668557	C	-3.01486	2.041126	0.668248
C	-2.92521	0.63162	2.242391	C	-3.01415	1.159388	1.807344	C	-3.01422	1.159501	1.807154
C	-2.69235	-0.65184	1.848615	C	-2.70995	-0.16889	1.858468	C	-2.71007	-0.16878	1.858397
C	-2.05309	-0.95193	0.484839	C	-2.03326	-0.86777	0.671547	C	-2.03327	-0.86768	0.671532
C	-3.08202	-0.45732	-0.52482	C	-3.07282	-0.79139	-0.43979	C	-3.07297	-0.79157	-0.43965
C	-3.44467	0.839706	-0.72282	C	-3.49391	0.348602	-1.05583	C	-3.49415	0.348303	-1.05588
C	-2.0289	-2.48454	0.177706	C	-1.92415	-2.41207	0.888292	C	-1.92397	-2.41194	0.888485
C	-3.0615	-2.75664	-0.82228	C	-2.92663	-3.05463	0.038021	C	-2.92668	-3.05471	0.038623
C	-3.63337	-1.6042	-1.25066	C	-3.55227	-2.14076	-0.74486	C	-3.55247	-2.14102	-0.74434
C	-3.21234	3.367154	-0.49813	C	-3.42516	2.802409	-1.69718	C	-3.42541	2.802068	-1.69754
C	-3.10916	-1.80449	2.722544	C	-3.08449	-0.99039	3.062905	C	-3.08451	-0.9902	3.062908
C	-4.68134	-1.48133	-2.31212	C	-4.58998	-2.43142	-1.78352	C	-4.59043	-2.43181	-1.7827
O	-1.27757	-3.29932	0.690649	O	-1.13902	-2.9688	1.639764	O	-1.13853	-2.96849	1.639751
C	0.109871	-0.81223	-0.951	C	0.143752	-1.09031	-0.73476	C	0.143646	-1.09021	-0.73475
C	-0.65179	-0.2901	0.279707	C	-0.66657	-0.23478	0.254062	C	-0.66672	-0.23454	0.253945
C	0.428045	-0.19222	1.416935	C	0.386096	0.296527	1.291843	C	0.385914	0.29721	1.291494
C	1.718453	-0.14289	0.611882	C	1.684074	0.157218	0.510268	C	1.683937	0.157501	0.510076
C	1.55494	-0.57088	-0.70728	C	1.567851	-0.69138	-0.59282	C	1.567759	-0.69137	-0.59279
C	2.959803	0.246899	1.089872	C	2.890587	0.763338	0.823365	C	2.890477	0.76361	0.823167
C	4.08298	0.201883	0.256745	C	4.032336	0.499317	0.05896	C	4.032296	0.499288	0.058999
C	3.922019	-0.26364	-1.08728	C	3.924332	-0.40176	-1.0482	C	3.924375	-0.4022	-1.04786
C	2.651575	-0.66229	-1.58146	C	2.681429	-0.99653	-1.39411	C	2.681458	-0.99691	-1.3938
O	0.339611	1.040851	2.133721	O	0.213131	1.68852	1.564947	O	0.212946	1.689367	1.563914
C	0.4883	-1.36574	2.408148	C	0.498431	-0.48103	2.613538	C	0.498115	-0.47965	2.613619
C	5.429594	0.660891	0.811179	C	5.348345	1.168242	0.449928	C	5.348338	1.168223	0.449906
O	-0.42086	-1.28396	-1.94401	O	-0.34198	-1.89909	-1.50942	O	-0.34215	-1.899	-1.50937
C	5.600872	0.399294	2.317205	C	6.148146	0.251764	1.400529	C	6.148202	0.251787	1.400482

C	-4.65402	3.730861	-0.90447	C	-2.20511	3.73617	-1.78408	C	-2.20424	3.73426	-1.78599
C	-2.24399	3.542209	-1.68221	C	-4.72273	3.602987	-1.47466	C	-4.72171	3.604371	-1.47391
C	5.653063	2.15443	0.490222	C	5.170949	2.566075	1.067064	C	5.17103	2.566108	1.066956
H	-0.83875	0.764064	0.042073	H	-0.90646	0.668448	-0.3198	H	-0.90673	0.66849	-0.32018
C	2.438856	-1.14475	-3.00329	C	2.52009	-1.94074	-2.57028	C	2.520071	-1.94137	-2.56978
C	5.068442	-0.37612	-2.02362	C	5.087896	-0.73646	-1.90657	C	5.088049	-0.73748	-1.90586
O	6.168175	0.140461	-1.90258	O	6.265132	-0.52098	-1.66394	O	6.265219	-0.52117	-1.66362
H	-2.77487	2.764036	2.023316	H	-2.98432	3.103304	0.905127	H	-2.98451	3.103294	0.904655
H	-3.26401	0.772181	3.269518	H	-3.37301	1.611618	2.732664	H	-3.373	1.611883	2.732435
H	-4.07884	1.046526	-1.5825	H	-4.11804	0.233806	-1.94135	H	-4.11836	0.233372	-1.94132
H	-3.27812	-3.75676	-1.17786	H	-3.08564	-4.12621	0.033632	H	-3.08569	-4.12629	0.034468
H	-2.90929	4.074275	0.284187	H	-3.51483	2.298242	-2.66795	H	-3.51674	2.297726	-2.66806
H	-3.50996	-1.45356	3.676636	H	-3.52847	-0.36966	3.845009	H	-3.52869	-0.36946	3.844895
H	-2.29023	-2.50693	2.903341	H	-3.82617	-1.74985	2.778123	H	-3.82602	-1.74983	2.778134
H	-3.89726	-2.38452	2.222144	H	-2.23293	-1.54331	3.470698	H	-2.23286	-1.54287	3.470868
H	-5.57216	-0.96916	-1.93182	H	-5.51104	-1.8702	-1.59089	H	-5.51162	-1.87096	-1.58957
H	-4.97871	-2.46136	-2.69089	H	-4.23226	-2.13306	-2.77598	H	-4.2332	-2.13303	-2.77521
H	-4.30478	-0.89029	-3.15524	H	-4.83137	-3.49587	-1.81586	H	-4.83146	-3.49634	-1.81525
H	3.046914	0.593045	2.11229	H	2.935741	1.449557	1.659901	H	2.935527	1.450056	1.659517
H	-0.49128	1.012578	2.629456	H	-0.62425	1.773588	2.043293	H	-0.62432	1.774609	2.042424
H	0.49489	-2.32966	1.898803	H	-0.3825	-0.30131	3.235049	H	-0.38287	-0.29948	3.234929
H	-0.37855	-1.34513	3.073674	H	1.374287	-0.12327	3.161654	H	1.373945	-0.12166	3.161624
H	1.385933	-1.26459	3.024228	H	0.577737	-1.55615	2.450382	H	0.577368	-1.55486	2.451011
H	6.210844	0.108359	0.286853	H	5.947138	1.277981	-0.45549	H	5.947057	1.277892	-0.45557
H	5.380722	-0.64093	2.578005	H	5.593849	0.070999	2.328475	H	5.594014	0.071124	2.328515
H	4.965904	1.045358	2.93272	H	6.364805	-0.70806	0.927752	H	6.364755	-0.70811	0.927781
H	6.637281	0.604726	2.603568	H	7.102242	0.722226	1.661048	H	7.102355	0.72222	1.660836
H	-5.34198	3.629374	-0.0596	H	-2.06219	4.299585	-0.85612	H	-2.05945	4.29745	-0.85818
H	-5.02177	3.094223	-1.71664	H	-2.33645	4.462048	-2.5937	H	-2.33561	4.460285	-2.59547
H	-4.69896	4.766095	-1.25887	H	-1.28552	3.175191	-1.97587	H	-1.28562	3.172071	-1.9789
H	-2.48571	2.855201	-2.50055	H	-4.87276	4.330014	-2.2802	H	-4.87165	4.331303	-2.27955
H	-1.20958	3.350869	-1.38047	H	-4.68878	4.154479	-0.52889	H	-4.68608	4.156193	-0.52839
H	-2.29812	4.562469	-2.07683	H	-5.59711	2.945036	-1.44437	H	-5.59689	2.947546	-1.44247
H	4.882578	2.775531	0.960701	H	4.729146	2.532664	2.068623	H	4.729399	2.532807	2.068591
H	5.636218	2.328433	-0.58741	H	6.151276	3.042792	1.167008	H	6.151376	3.042831	1.166689
H	6.628541	2.477992	0.868716	H	4.544755	3.211219	0.442743	H	4.544734	3.21118	0.442665
H	2.961484	-2.0908	-3.18498	H	3.188296	-2.80355	-2.4866	H	3.188333	-2.80412	-2.48608
H	2.809421	-0.42294	-3.73751	H	2.74721	-1.44507	-3.5207	H	2.746989	-1.4458	-3.52031
H	1.378952	-1.31368	-3.18399	H	1.496091	-2.30673	-2.61427	H	1.496077	-2.30739	-2.6136
H	4.871554	-1.00378	-2.91255	H	4.825376	-1.23358	-2.8585	H	4.825683	-1.23605	-2.85704
		**4b4 4b5**									
C	-3.24276	1.70431	-0.64413	C	-3.22834	1.698565	-0.62251				
C	-3.01523	2.041043	0.668288	C	-2.99498	2.041876	0.685659				
C	-3.01431	1.15945	1.807226	C	-2.98833	1.162758	1.828736				
C	-2.70991	-0.16878	1.858504	C	-2.67708	-0.16265	1.88513				
C	-2.03321	-0.86772	0.671613	C	-2.00963	-0.86539	0.694681				
C	-3.07289	-0.79172	-0.43961	C	-3.05576	-0.79745	-0.41142				
C	-3.49429	0.348101	-1.05578	C	-3.48614	0.338676	-1.0253				
C	-1.92375	-2.41198	0.888576	C	-1.89341	-2.40813	0.917003				
C	-2.92624	-3.05485	0.038546	C	-2.89678	-3.05837	0.073281				
C	-3.55214	-2.14122	-0.74441	C	-3.52956	-2.15049	-0.71054				
C	-3.42598	2.801857	-1.6975	C	-3.43367	2.772558	-1.69532				
C	-3.08415	-0.99013	3.063144	C	-3.03615	-0.97903	3.097996				
C	-4.59005	-2.43217	-1.78279	C	-4.56976	-2.44916	-1.74435				
O	-1.1384	-2.96843	1.640016	O	-1.10225	-2.95855	1.6668				

C	0.143704	-1.09015	-0.73476	C	0.158736	-1.09049	-0.72689
C	-0.6667	-0.23451	0.25388	C	-0.64764	-0.23088	0.261758
C	0.385924	0.297328	1.291433	C	0.410399	0.313235	1.286921
C	1.683934	0.157768	0.509955	C	1.703574	0.171256	0.497983
C	1.567772	-0.69109	-0.59293	C	1.582507	-0.68595	-0.59778
C	2.89048	0.763821	0.823125	C	2.910547	0.782603	0.799097
C	4.032325	0.499443	0.059007	C	4.048244	0.515462	0.029841
C	3.924357	-0.40184	-1.048	C	3.93527	-0.39333	-1.07056
C	2.681467	-0.99657	-1.39394	C	2.691733	-0.99376	-1.40415
O	0.21285	1.689414	1.563969	O	0.234181	1.707229	1.548202
C	0.498271	-0.47967	2.613481	C	0.534602	-0.45196	2.614793
C	5.348439	1.168088	0.450083	C	5.365506	1.188767	0.408867
O	-0.34199	-1.89911	-1.50927	O	-0.32957	-1.90571	-1.49314
C	6.148138	0.251229	1.400422	C	6.173954	0.277588	1.35731
C	-2.20478	3.734003	-1.78618	C	-2.51305	3.992238	-1.5377
C	-4.72223	3.604187	-1.47373	C	-4.91236	3.206045	-1.7646
C	5.171338	2.565791	1.067585	C	5.189717	2.588443	1.022191
H	-0.90686	0.668474	-0.32026	H	-0.89528	0.666535	-0.31783
C	2.520209	-1.94103	-2.56993	C	2.525247	-1.9464	-2.57281
C	5.08789	-0.73658	-1.90637	C	5.093779	-0.73015	-1.93491
O	6.265129	-0.52085	-1.66392	O	6.271927	-0.50781	-1.70308
H	-2.9851	3.103223	0.904673	H	-2.95615	3.10341	0.918953
H	-3.37305	1.611782	2.732541	H	-3.34431	1.618366	2.753513
H	-4.11852	0.233042	-1.94119	H	-4.11567	0.219988	-1.9067
H	-3.08505	-4.12646	0.034343	H	-3.05062	-4.13072	0.073186
H	-3.51747	2.297465	-2.66799	H	-3.18447	2.30435	-2.65705
H	-3.52817	-0.36933	3.845177	H	-3.47424	-0.35524	3.881021
H	-3.82575	-1.74974	2.778544	H	-3.7781	-1.74259	2.825294
H	-2.23246	-1.54282	3.470948	H	-2.17858	-1.52641	3.500398
H	-5.51177	-1.87242	-1.58892	H	-5.49297	-1.89223	-1.54937
H	-4.23343	-2.13219	-2.77514	H	-4.21747	-2.15124	-2.73889
H	-4.83002	-3.49691	-1.81613	H	-4.80586	-3.5149	-1.77329
H	2.935563	1.450226	1.65951	H	2.959155	1.474941	1.630385
H	-0.62475	1.774626	2.041904	H	-0.60033	1.793267	2.031461
H	-0.38265	-0.29957	3.234886	H	-0.34131	-0.26725	3.241775
H	1.374128	-0.12167	3.161434	H	1.414562	-0.08853	3.152452
H	0.577561	-1.55486	2.45076	H	0.613849	-1.52846	2.460461
H	5.947209	1.277918	-0.45534	H	5.957633	1.296277	-0.50123
H	5.593853	0.07035	2.32835	H	5.626754	0.099668	2.290006
H	6.364606	-0.70853	0.927409	H	6.389052	-0.6838	0.887019
H	7.102331	0.721465	1.660996	H	7.129024	0.750897	1.608957
H	-2.05989	4.297287	-0.85844	H	-2.77324	4.591855	-0.65883
H	-2.33621	4.459961	-2.59572	H	-2.60537	4.647	-2.41026
H	-1.28621	3.171758	-1.97912	H	-1.46444	3.695268	-1.44236
H	-4.87226	4.331075	-2.2794	H	-5.07307	3.911389	-2.58716
H	-4.6865	4.156074	-0.52826	H	-5.21406	3.69622	-0.83256
H	-5.59742	2.947371	-1.44216	H	-5.57767	2.350672	-1.92025
H	4.729613	2.53219	2.069173	H	4.755333	2.55764	2.027085
H	6.151754	3.042311	1.167578	H	6.169759	3.067495	1.113295
H	4.54521	3.211214	0.443486	H	4.557657	3.230098	0.400194
H	3.189018	-2.80338	-2.48658	H	3.197706	-2.80584	-2.48895
H	2.746519	-1.4452	-3.52049	H	2.742733	-1.45578	-3.52812
H	1.496399	-2.3076	-2.61346	H	1.502431	-2.31676	-2.60651
H	4.825345	-1.23393	-2.85816	H	4.826124	-1.2359	-2.88082

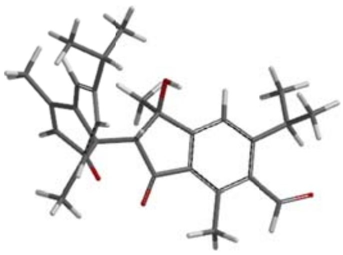

4c1 (47.3%, [α]$_D$ = -746.2)

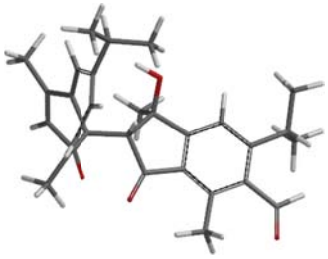

4c2 (20.4%, [α]$_D$ = -698.0)

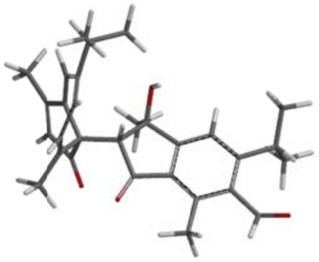

4c3 (15.3%, [α]$_D$ = -627.6)

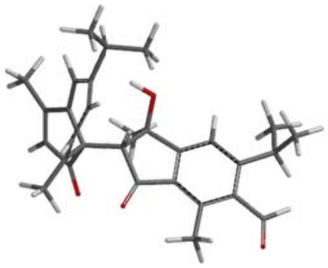

4c4 (11.4%, [α]$_D$ = -731.7)

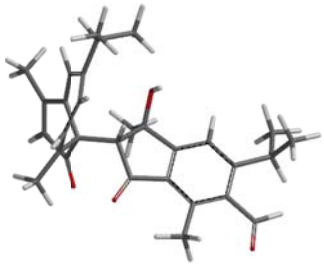

4c5 (5.0%)

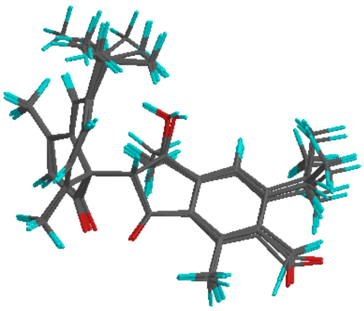

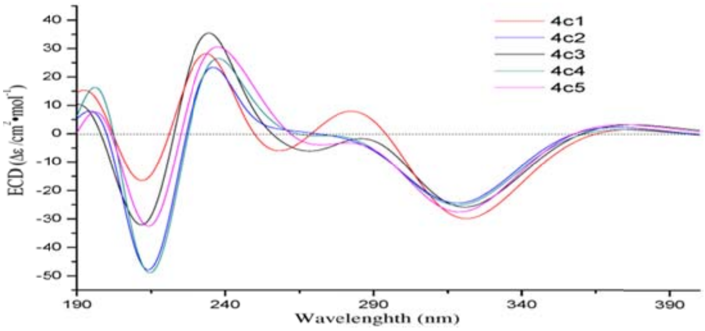

Figure S8. Structural candidates of compound **4** with 10*R*,1'*R*,2'*R* absolute configuration, and the optical rotation and overlaid ECD calculated on Gaussian program.

Table S14. Important thermodynamic parameters (a.u.) of the optimized **4c** with 10R,1'R,2'R absolute configuration at B3LYP/6-31G(d,p) level in the gas phase

conformations	E+ZPE	G
**4c1**	-1464.009853	-1464.073922
**4c2**	-1464.009104	-1464.073130
**4c3**	-1464.009638	-1464.072855
**4c4**	-1464.009061	-1464.072587
**4c5**	-1464.007597	-1464.071804

Table S15. Optimized Z-Matrixes of compound **4c** with 10R,1'R,2'R absolute configuration in the Gas Phase (Å) at B3LYP/6-31g(d,p) level.

4c1				4c2				4c3			
C	3.369424	1.29254	-1.21928	C	3.193931	1.617664	-0.89313	C	3.339021	1.592292	-0.89699
C	3.119537	0.350662	-2.18993	C	3.011047	0.824215	-2.00086	C	3.147622	0.801057	-2.00417
C	3.01911	-1.07596	-2.03239	C	3.026655	-0.61112	-2.07912	C	3.11527	-0.63674	-2.06856
C	2.613992	-1.79357	-0.94892	C	2.709817	-1.5323	-1.12721	C	2.756505	-1.54139	-1.11741
C	1.94055	-1.08364	0.238298	C	2.034856	-1.09555	0.183071	C	2.062292	-1.08145	0.175685
C	3.033274	-0.17248	0.77806	C	3.06174	-0.18517	0.845842	C	3.089898	-0.18856	0.855497
C	3.557061	0.920209	0.156105	C	3.450232	1.047144	0.402625	C	3.53597	1.018256	0.409991
C	1.708101	-2.05186	1.437671	C	1.943781	-2.27108	1.204285	C	1.920446	-2.24945	1.198949
C	2.746469	-1.77881	2.433442	C	2.98326	-2.05644	2.217787	C	2.926597	-2.04452	2.24378

C	3.472854	-0.68651	2.077437	C	3.593259	-0.85606	2.034815	C	3.563685	-0.85624	2.069659
C	3.678986	2.730822	-1.63327	C	3.276992	3.140219	-1.02944	C	3.527937	3.106713	-1.00168
C	2.797718	-3.2826	-0.89229	C	2.987337	-2.99137	-1.34335	C	3.017424	-3.00715	-1.31381
C	4.583956	-0.07402	2.873704	C	4.673383	-0.27682	2.896679	C	4.619403	-0.28708	2.96718
O	0.791392	-2.85336	1.527178	O	1.130625	-3.17887	1.169682	O	1.091663	-3.14341	1.137824
C	-0.43757	-1.42279	-0.74791	C	-0.37634	-1.34936	-0.81801	C	-0.30901	-1.32823	-0.89914
C	0.608427	-0.41371	-0.21544	C	0.65195	-0.4471	-0.10233	C	0.689571	-0.42122	-0.14288
C	-0.18778	0.568576	0.715673	C	-0.15862	0.280111	1.019713	C	-0.16694	0.295324	0.961561
C	-1.6366	0.276152	0.341532	C	-1.57395	0.203734	0.47258	C	-1.57629	0.165044	0.394428
C	-1.7863	-0.88675	-0.41505	C	-1.72312	-0.7896	-0.49771	C	-1.67508	-0.81707	-0.59315
C	-2.73271	1.052562	0.690585	C	-2.64651	0.982407	0.878277	C	-2.68363	0.899818	0.79053
C	-4.0225	0.667139	0.309772	C	-3.91437	0.755235	0.336462	C	-3.94119	0.635946	0.236004
C	-4.18067	-0.5439	-0.4358	C	-4.08572	-0.2947	-0.62056	C	-4.05256	-0.3994	-0.74573
C	-3.06057	-1.3353	-0.80608	C	-2.98019	-1.07557	-1.06351	C	-2.91272	-1.12835	-1.18204
O	0.131849	1.944055	0.459648	O	0.193957	1.663367	1.136698	O	0.197136	1.664043	1.154712
C	0.000997	0.402149	2.22642	C	-0.13136	-0.37037	2.414592	C	-0.1328	-0.31812	2.366437
C	-5.20258	1.558192	0.691078	C	-5.08144	1.622476	0.824956	C	-5.14236	1.447927	0.715972
O	-0.17821	-2.40751	-1.41634	O	-0.10361	-2.26658	-1.5696	O	-0.00614	-2.21692	-1.67453
C	-5.52077	2.53695	-0.45952	C	-5.82137	0.928321	1.988288	C	-4.79349	2.894405	1.106163
C	5.198352	2.972409	-1.73754	C	4.339988	3.60729	-2.03924	C	2.28223	3.850723	-0.47615
C	3.022506	3.771277	-0.70917	C	1.893815	3.737211	-1.35679	C	3.914094	3.623342	-2.39339
C	-5.00336	2.320904	2.011726	C	-4.66734	3.048028	1.23079	C	-5.84158	0.722818	1.885665
H	0.863524	0.187977	-1.09694	H	0.830629	0.350764	-0.83365	H	0.904951	0.370895	-0.87346
C	-3.19433	-2.61153	-1.61384	C	-3.11097	-2.16776	-2.09468	C	-2.99019	-2.22124	-2.23069
C	-5.51861	-1.04959	-0.83687	C	-5.44752	-0.57203	-1.14393	C	-5.35047	-0.74562	-1.37885
O	-6.56215	-0.41651	-0.86417	O	-5.7416	-1.39942	-1.99115	O	-6.46246	-0.41473	-0.99814
H	3.14732	0.70262	-3.22138	H	2.977816	1.331939	-2.96337	H	3.165744	1.295023	-2.97214
H	3.331292	-1.65239	-2.90238	H	3.348395	-1.00899	-3.04065	H	3.452567	-1.05111	-3.01787
H	4.228842	1.548276	0.738788	H	4.085162	1.639221	1.062449	H	4.162404	1.596583	1.08864
H	2.862918	-2.36454	3.337513	H	3.18881	-2.77021	3.006805	H	3.090013	-2.75156	3.048475
H	3.25903	2.864566	-2.63882	H	3.572594	3.529293	-0.0456	H	4.356272	3.357284	-0.3223
H	1.837611	-3.78729	-0.75616	H	2.062996	-3.57018	-1.26697	H	2.087918	-3.57738	-1.24146
H	3.431989	-3.55838	-0.0381	H	3.448963	-3.16436	-2.31829	H	3.689983	-3.38598	-0.53152
H	3.278182	-3.65447	-1.80046	H	3.671621	-3.37261	-0.57251	H	3.488625	-3.19589	-2.28135
H	5.51499	-0.05786	2.296522	H	4.363043	0.684159	3.323808	H	5.54953	-0.11368	2.414836
H	4.75937	-0.62519	3.79999	H	4.927023	-0.94811	3.719725	H	4.831905	-0.95632	3.803482
H	4.351425	0.965477	3.133164	H	5.579281	-0.08649	2.310708	H	4.304599	0.680832	3.374437
H	-2.57781	1.962673	1.258119	H	-2.48303	1.77118	1.601963	H	-2.5612	1.681441	1.531138
H	-0.11669	2.137501	-0.45492	H	1.116147	1.693405	1.42951	H	0.116498	2.121285	0.30648
H	1.025702	0.645589	2.510858	H	-0.43541	-1.41847	2.37861	H	0.859402	-0.20808	2.806974
H	-0.23543	-0.61322	2.546978	H	0.868481	-0.31862	2.854611	H	-0.40264	-1.37466	2.346211
H	-0.66727	1.096261	2.742274	H	-0.81759	0.173819	3.068858	H	-0.84575	0.217143	2.998861
H	-6.0783	0.917428	0.806413	H	-5.79056	1.743441	0.000999	H	-5.86328	1.488929	-0.10183
H	-5.76602	1.9956	-1.37517	H	-5.14871	0.789667	2.841414	H	-4.23444	3.406569	0.316349
H	-4.67035	3.199637	-0.65702	H	-6.67018	1.535309	2.320659	H	-4.20777	2.953897	2.029624
H	-6.38157	3.160241	-0.19518	H	-6.1995	-0.0574	1.701588	H	-5.71804	3.454217	1.27928
H	5.661833	2.279939	-2.44658	H	4.408679	4.700395	-2.04398	H	2.003913	3.508998	0.524528
H	5.689783	2.836742	-0.76728	H	4.094048	3.291225	-3.05843	H	1.429412	3.683439	-1.14672
H	5.405863	3.995039	-2.07141	H	5.328072	3.205102	-1.79423	H	2.460102	4.930723	-0.43435
H	3.492866	3.780192	0.280309	H	1.565244	3.431457	-2.35646	H	3.103194	3.488651	-3.11791
H	1.961238	3.560963	-0.55584	H	1.138046	3.400363	-0.64144	H	4.802888	3.116615	-2.78148
H	3.126477	4.777381	-1.12898	H	1.930719	4.832076	-1.33902	H	4.128991	4.695618	-2.34737
H	-4.25175	3.113682	1.932661	H	-5.56396	3.650765	1.406295	H	-5.16576	0.617729	2.741902
H	-4.70813	1.653241	2.827429	H	-4.08347	3.064872	2.156382	H	-6.18166	-0.26906	1.582204
H	-5.94447	2.802151	2.295861	H	-4.07833	3.536219	0.448839	H	-6.71684	1.294518	2.211924
H	-3.76557	-2.45327	-2.53339	H	-3.82442	-2.92729	-1.76281	H	-3.32183	-1.82947	-3.19884
H	-3.70889	-3.39077	-1.03983	H	-2.14382	-2.629	-2.2806	H	-3.69479	-3.00614	-1.93889
H	-2.20824	-2.98897	-1.87672	H	-3.52444	-1.77565	-3.02772	H	-2.01026	-2.67401	-2.36729
H	-5.53461	-2.11539	-1.13018	H	-6.25677	0.03468	-0.69794	H	-5.26426	-1.37024	-2.28664
		**4c4 4c5**									
C	3.092745	1.70927	-0.90607	C	3.215131	1.60207	-1.05471				
C	2.902897	0.939596	-2.02937	C	2.976748	0.769595	-2.1217				
C	2.967504	-0.4916	-2.14806	C	2.97269	-0.66984	-2.13625				
C	2.714454	-1.44895	-1.21275	C	2.690208	-1.54882	-1.13652				

C	2.067622	-1.07225	0.130028	C	2.063552	-1.05973	0.180243
C	3.084145	-0.14404	0.784236	C	3.108922	-0.12206	0.76798
C	3.412523	1.113641	0.364039	C	3.500863	1.077401	0.256598
C	2.051897	-2.27744	1.119982	C	2.009862	-2.19529	1.247602
C	3.117726	-2.05372	2.103871	C	3.069959	-1.93229	2.224061
C	3.679138	-0.82789	1.935056	C	3.667318	-0.73712	1.973947
C	3.115034	3.237017	-1.00216	C	3.362099	3.1158	-1.21974
C	3.036071	-2.89069	-1.47859	C	2.971347	-3.01471	-1.30076
C	4.768042	-0.23459	2.775978	C	4.758717	-0.11384	2.788897
O	1.26965	-3.21194	1.08695	O	1.201388	-3.10925	1.265522
C	-0.36694	-1.38379	-0.79631	C	-0.36204	-1.38409	-0.75056
C	0.655067	-0.46529	-0.09268	C	0.660693	-0.4376	-0.07674
C	-0.13892	0.206429	1.074538	C	-0.14601	0.291224	1.054434
C	-1.56971	0.093302	0.576039	C	-1.58035	0.128396	0.563538
C	-1.71963	-0.8784	-0.41535	C	-1.71994	-0.87612	-0.39465
C	-2.65508	0.812756	1.05165	C	-2.67394	0.856309	1.009475
C	-3.93365	0.55519	0.550638	C	-3.94929	0.57129	0.515544
C	-4.099	-0.45319	-0.45193	C	-4.1024	-0.46934	-0.45595
C	-2.98569	-1.1909	-0.94635	C	-2.98157	-1.21458	-0.92176
O	0.169557	1.598456	1.212542	O	0.20438	1.670121	1.198512
C	-0.03837	-0.47495	2.451171	C	-0.02943	-0.28832	2.46918
C	-5.10545	1.397847	1.0694	C	-5.13032	1.418852	1.004603
O	-0.09012	-2.27099	-1.58165	O	-0.08235	-2.29765	-1.50349
C	-4.91142	1.925222	2.501633	C	-4.94353	1.995832	2.418484
C	4.130064	3.77076	-2.0282	C	3.63961	3.594324	-2.65031
C	1.701789	3.790221	-1.27427	C	2.138625	3.850809	-0.63278
C	-5.39325	2.569388	0.105739	C	-5.42986	2.553751	0.001508
H	0.782643	0.3547	-0.81008	H	0.816136	0.337012	-0.84083
C	-3.11622	-2.26239	-1.99903	C	-3.1003	-2.32021	-1.93987
C	-5.45883	-0.71467	-0.98814	C	-5.4582	-0.75731	-0.98979
O	-5.75475	-1.5392	-1.83761	O	-5.74116	-1.59688	-1.82863
H	2.820248	1.47119	-2.97581	H	2.926368	1.229875	-3.10504
H	3.271923	-0.85155	-3.12995	H	3.262362	-1.10937	-3.08994
H	4.047514	1.71075	1.019309	H	4.153333	1.691955	0.876563
H	3.374692	-2.78125	2.864734	H	3.296315	-2.60805	3.040289
H	3.424609	3.610558	-0.01656	H	4.229002	3.406047	-0.60747
H	2.13525	-3.50433	-1.39261	H	3.693893	-3.35438	-0.54556
H	3.474525	-3.01995	-2.47085	H	2.059509	-3.60022	-1.15906
H	3.755675	-3.26853	-0.73889	H	3.392238	-3.22575	-2.28669
H	4.442299	0.706099	3.235429	H	4.442539	0.858687	3.183912
H	5.068957	-0.9161	3.574385	H	5.038115	-0.74981	3.631595
H	5.648359	-0.00269	2.166433	H	5.649927	0.064334	2.177204
H	-2.49494	1.572163	1.806454	H	-2.52079	1.64114	1.740206
H	1.104766	1.655417	1.456464	H	0.095144	2.100418	0.339466
H	-0.3088	-1.53172	2.400881	H	-0.28074	-1.34942	2.487386
H	0.97448	-0.39923	2.856429	H	0.981915	-0.15115	2.855237
H	-0.71802	0.030165	3.142565	H	-0.71823	0.250068	3.125454
H	-5.9973	0.765091	1.104567	H	-6.0155	0.778292	1.059888
H	-4.13991	2.699557	2.558312	H	-4.17982	2.779361	2.450515
H	-5.84506	2.376387	2.851812	H	-5.88199	2.449084	2.752551
H	-4.6426	1.12371	3.196255	H	-4.66743	1.220783	3.139607
H	4.155497	4.865529	-2.00686	H	3.832153	4.671836	-2.65339
H	3.86806	3.469966	-3.04802	H	2.783965	3.418396	-3.31176
H	5.139717	3.402569	-1.82049	H	4.511115	3.093979	-3.08332
H	1.356445	3.499928	-2.2729	H	1.245743	3.644113	-1.23702
H	0.979115	3.406111	-0.54849	H	1.938263	3.536543	0.395274
H	1.699121	4.884805	-1.22655	H	2.293835	4.935099	-0.63691
H	-6.2445	3.159421	0.461624	H	-6.28977	3.144551	0.334355
H	-4.52379	3.232068	0.040402	H	-4.5691	3.225819	-0.08268
H	-5.62072	2.220464	-0.90578	H	-5.6489	2.168368	-0.99858
H	-3.7963	-3.05043	-1.66402	H	-3.78524	-3.09627	-1.5874
H	-2.14208	-2.68971	-2.22515	H	-2.12381	-2.75484	-2.13985
H	-3.56975	-1.86163	-2.90971	H	-3.54253	-1.94947	-2.86871
H	-6.26232	-0.08546	-0.56379	H	-6.27117	-0.13424	-0.57454

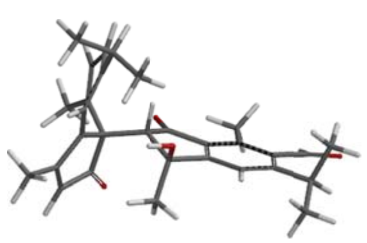

4d1 (47.3%, [α]$_D$ = 693.4)

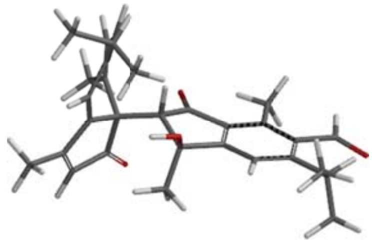

4d2 (27.3%, [α]$_D$ = 568.8)

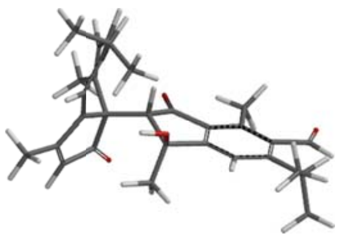

4d3 (17.3%, α]$_D$ = 589.9)

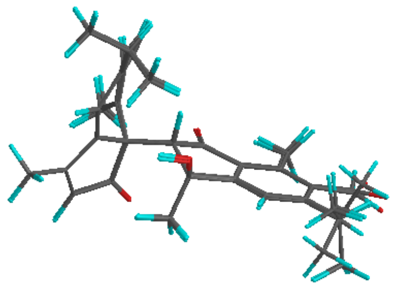

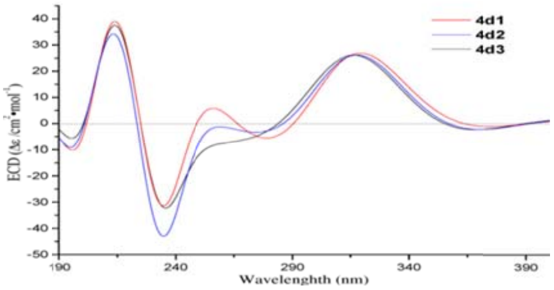

Figure S9. Structural candidates of compound **4** with 10*S*,1'*S*,2'*S* absolute configuration, and the optical rotation and overlaid ECD calculated on Gaussian program.

Table S16. Important thermodynamic parameters (a.u.) of the optimized **4d** with 10S,1'S,2'S absolute configuration at B3LYP/6-31G(d,p) level in the gas phase

conformations	E+ZPE	G
**4d1**	-1464.011774	-1464.074928
**4d2**	-1464.011620	-1464.074410
**4d3**	-1464.009848	-1464.073978

Table S17. Optimized Z-Matrixes of compound **4** with 10R,1'S,2'S absolute configuration in the Gas Phase (Å) at B3LYP/6-31g(d,p) level.

4d1				4d2				4d3			
C	-3.11885	1.479636	-1.27184	C	-3.24657	1.367007	-1.24569	C	-3.22512	1.26863	-1.33372
C	-2.898	0.553086	-2.2622	C	-3.03853	0.412803	-2.21246	C	-2.98041	0.275655	-2.25158
C	-2.93268	-0.88187	-2.1638	C	-3.01366	-1.01777	-2.06177	C	-2.93102	-1.14571	-2.03517
C	-2.6655	-1.68012	-1.09466	C	-2.66575	-1.76551	-0.97925	C	-2.59016	-1.83648	-0.91325
C	-2.03141	-1.08961	0.175614	C	-1.99186	-1.10549	0.235544	C	-1.95474	-1.10812	0.283387
C	-3.06523	-0.08927	0.683405	C	-3.03681	-0.12495	0.758493	C	-3.02966	-0.12665	0.740181
C	-3.42968	1.077584	0.076141	C	-3.47463	1.00476	0.129726	C	-3.47557	0.96463	0.051878
C	-1.99593	-2.12934	1.336396	C	-1.86096	-2.09925	1.429314	C	-1.82983	-2.04439	1.523642
C	-3.06487	-1.77836	2.278007	C	-2.89732	-1.75391	2.408625	C	-2.89279	-1.67564	2.465579
C	-3.64727	-0.60211	1.92707	C	-3.53986	-0.61321	2.046014	C	-3.54966	-0.56589	2.038559
C	-3.23934	2.957347	-1.64014	C	-3.44485	2.824107	-1.66005	C	-3.43853	2.702118	-1.81748
C	-2.96277	-3.15107	-1.1374	C	-2.91172	-3.24617	-0.95292	C	-2.80597	-3.31935	-0.82473
C	-4.7459	0.091645	2.673029	C	-4.62839	0.066272	2.819261	C	-4.66804	0.124945	2.757466
O	-1.19586	-3.04356	1.443395	O	-1.02179	-2.97834	1.529267	O	-0.97741	-2.90194	1.680609
C	0.403023	-1.51772	-0.69132	C	0.41301	-1.48385	-0.72838	C	0.476333	-1.47387	-0.62274
C	-0.62336	-0.50784	-0.13505	C	-0.6234	-0.48738	-0.16704	C	-0.59235	-0.47852	-0.12049
C	0.167788	0.33907	0.918311	C	0.181061	0.433091	0.811544	C	0.174352	0.49675	0.832918
C	1.603211	0.141337	0.459269	C	1.60342	0.257697	0.305368	C	1.606206	0.33972	0.348962
C	1.752184	-0.97257	-0.37	C	1.755409	-0.88496	-0.48347	C	1.801776	-0.82788	-0.39097
C	2.690889	0.919561	0.82267	C	2.679676	1.082317	0.592545	C	2.652636	1.210211	0.612439
C	3.974487	0.589902	0.372745	C	3.959673	0.756791	0.129962	C	3.940154	0.90588	0.163201
C	4.135417	-0.57129	-0.44755	C	4.126906	-0.43794	-0.63919	C	4.158664	-0.31544	-0.55028
C	3.021377	-1.369	-0.82441	C	3.018415	-1.26406	-0.96904	C	3.080699	-1.19585	-0.85129
O	-0.13087	1.734676	0.821718	O	-0.16796	1.812206	0.6603	O	-0.20675	1.861096	0.629656
C	0.049195	-0.10659	2.38671	C	0.135547	0.055833	2.303163	C	0.120643	0.17148	2.33665
C	5.145711	1.489098	0.761882	C	5.124334	1.679673	0.481766	C	5.077834	1.879751	0.493924

O	0.135281	-2.50557	-1.35163	O	0.150857	-2.50301	-1.34173	O	0.244073	-2.52709	-1.18626
C	4.977039	2.170986	2.130168	C	4.729094	3.163775	0.570665	C	5.80664	1.442897	1.782847
C	-2.36647	3.856363	-0.74673	C	-2.5615	3.789699	-0.85012	C	-2.59164	3.71812	-1.03048
C	-4.70564	3.433602	-1.64065	C	-4.92693	3.243812	-1.59502	C	-4.92854	3.097825	-1.80603
C	5.392992	2.540492	-0.34132	C	5.794095	1.214929	1.792548	C	4.627257	3.34661	0.608285
H	-0.7559	0.197438	-0.96492	H	-0.82115	0.174813	-1.01901	H	-0.7888	0.143599	-1.00245
C	3.153481	-2.59423	-1.70813	C	3.155081	-2.52646	-1.79847	C	3.262404	-2.47403	-1.63008
C	5.468363	-1.01735	-0.92613	C	5.453448	-0.86095	-1.15434	C	5.539676	-0.66152	-0.9722
O	6.498837	-0.36249	-0.94287	O	6.544425	-0.44494	-0.79668	O	5.878475	-1.6559	-1.59302
H	-2.82232	0.943236	-3.2768	H	-3.02783	0.767517	-3.24278	H	-2.95438	0.583341	-3.29663
H	-3.2286	-1.39051	-3.08035	H	-3.33469	-1.57065	-2.94362	H	-3.22227	-1.74451	-2.89713
H	-4.08441	1.743997	0.63641	H	-4.12657	1.666928	0.698181	H	-4.15198	1.63852	0.57627
H	-3.30783	-2.38629	3.141493	H	-3.07584	-2.33855	3.303338	H	-3.07885	-2.22286	3.382185
H	-2.86791	3.054124	-2.66805	H	-3.13374	2.895347	-2.70982	H	-3.10325	2.732634	-2.86177
H	-3.68399	-3.42193	-0.35368	H	-3.37888	-3.58356	-1.88117	H	-3.48651	-3.56064	0.003769
H	-2.05502	-3.7318	-0.95374	H	-1.9755	-3.78844	-0.79641	H	-1.86184	-3.83305	-0.62548
H	-3.39092	-3.43739	-2.10094	H	-3.58119	-3.51064	-0.12248	H	-3.24685	-3.70806	-1.74568
H	-4.4388	1.098166	2.980724	H	-4.34705	1.09434	3.076071	H	-4.41688	1.171681	2.965641
H	-5.03015	-0.46512	3.568348	H	-5.54795	0.12658	2.226678	H	-4.89188	-0.366	3.70683
H	-5.63292	0.209135	2.040728	H	-4.8483	-0.4671	3.746394	H	-5.57795	0.131633	2.147123
H	2.534021	1.788695	1.449608	H	2.516687	1.983756	1.170557	H	2.453698	2.127385	1.152537
H	-1.05449	1.847058	1.0872	H	-1.08886	1.905609	0.94245	H	-1.13868	1.93606	0.879491
H	0.313802	-1.15805	2.515553	H	-0.87224	0.176674	2.709827	H	0.459004	-0.84559	2.544736
H	-0.9681	0.039127	2.760721	H	0.451398	-0.97573	2.471769	H	-0.89427	0.282119	2.72828
H	0.723852	0.50327	2.993146	H	0.805335	0.722496	2.85253	H	0.767663	0.87351	2.869201
H	6.041117	0.867169	0.807713	H	5.874699	1.582484	-0.30422	H	5.802044	1.855402	-0.32556
H	4.728296	1.451226	2.916687	H	4.190221	3.496351	-0.32219	H	6.2102	0.429395	1.701024
H	4.203706	2.946478	2.121954	H	5.633818	3.772948	0.664303	H	5.118559	1.457692	2.63486
H	5.916039	2.660219	2.407764	H	4.105225	3.383552	1.443533	H	6.635921	2.12297	2.004396
H	-1.3276	3.515699	-0.73157	H	-1.5103	3.490109	-0.88052	H	-1.53499	3.436628	-1.0225
H	-2.73335	3.864318	0.287113	H	-2.87204	3.824787	0.201515	H	-2.92819	3.792668	0.011004
H	-2.38731	4.891352	-1.10403	H	-2.64287	4.808314	-1.24396	H	-2.68112	4.717057	-1.47034
H	-5.14891	3.382488	-0.63948	H	-5.55173	2.587258	-2.20805	H	-5.33793	3.103047	-0.78927
H	-5.31971	2.823808	-2.31024	H	-5.31165	3.211289	-0.56927	H	-5.52705	2.404342	-2.40444
H	-4.77017	4.475742	-1.97157	H	-5.05145	4.270206	-1.95658	H	-5.06153	4.105159	-2.21517
H	4.517138	3.186602	-0.46837	H	6.643198	1.864041	2.031806	H	4.019078	3.527163	1.500204
H	5.617268	2.059453	-1.29525	H	6.167425	0.193345	1.69891	H	4.050421	3.662558	-0.26591
H	6.24623	3.172638	-0.07318	H	5.087877	1.257369	2.62958	H	5.50794	3.992024	0.684909
H	3.676174	-2.36792	-2.6423	H	3.861587	-3.22877	-1.34519	H	3.96094	-3.14078	-1.11647
H	2.167233	-2.98742	-1.94692	H	3.517154	-2.30768	-2.80924	H	3.71923	-2.27681	-2.60346
H	3.715867	-3.38847	-1.20391	H	2.189216	-3.02001	-1.8852	H	2.3065	-2.97584	-1.76069
H	5.494876	-2.05926	-1.29531	H	5.413095	-1.63662	-1.94092	H	6.319432	0.06296	-0.67415

Table S18. Calculated OR for the structural candidates of **4** at RB3LYP-SCRF(PCM, methanol)/6-311+G(2d,p) level

conformations	OR	configuration	OR_b
**4a1**	276.4	10*S*,1'*R*,2'*R*	**276.4**
**4b1**	-278.5		
**4b2**	-513.1		
**4b3**	-513.5	10*R*,1'*S*,2'*S*	**-402.2**
**4b4**	-513.3		
**4b5**	-457.7		
**4c1**	-746.2		
**4c2**	-698.0	10R,1'*R*,2'*R*	**-675.7**
**4c3**	-627.6		
**4c4**	-731.7		
**4d1**	693.4		
**4d2**	568.8	10*S*,1'*S*,2'*S*	**584.6**
**4d3**	568.9		

OR_b: The combined OR values after Boltzmann weighting according to their population contribution.

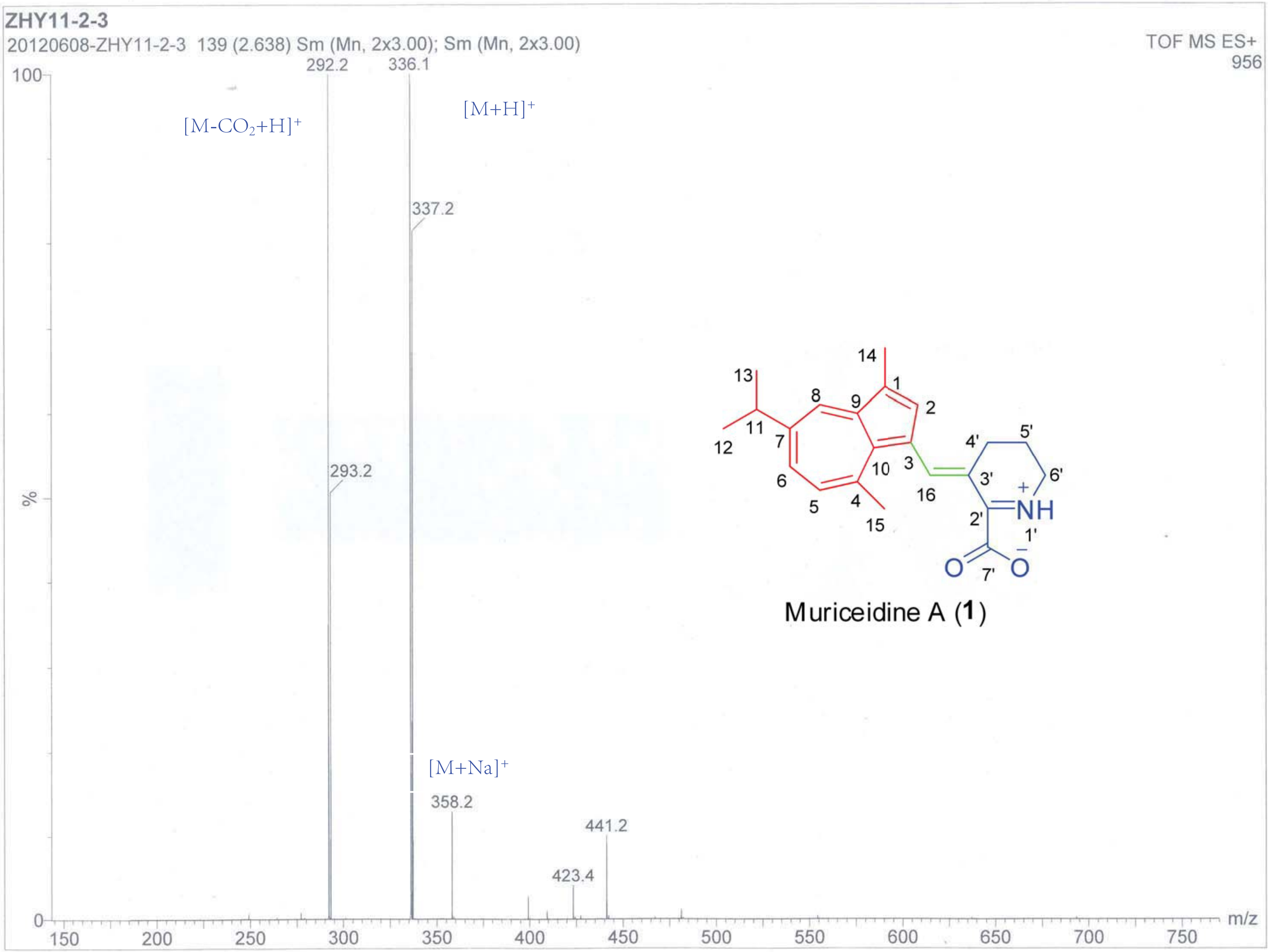

SS1 The positive ESIMS spectrum of muriceidine A (1)

20120706-ZHY-11-2-3_120706105310 7/6/2012 11:23:22 AM ZHY-11-2-3

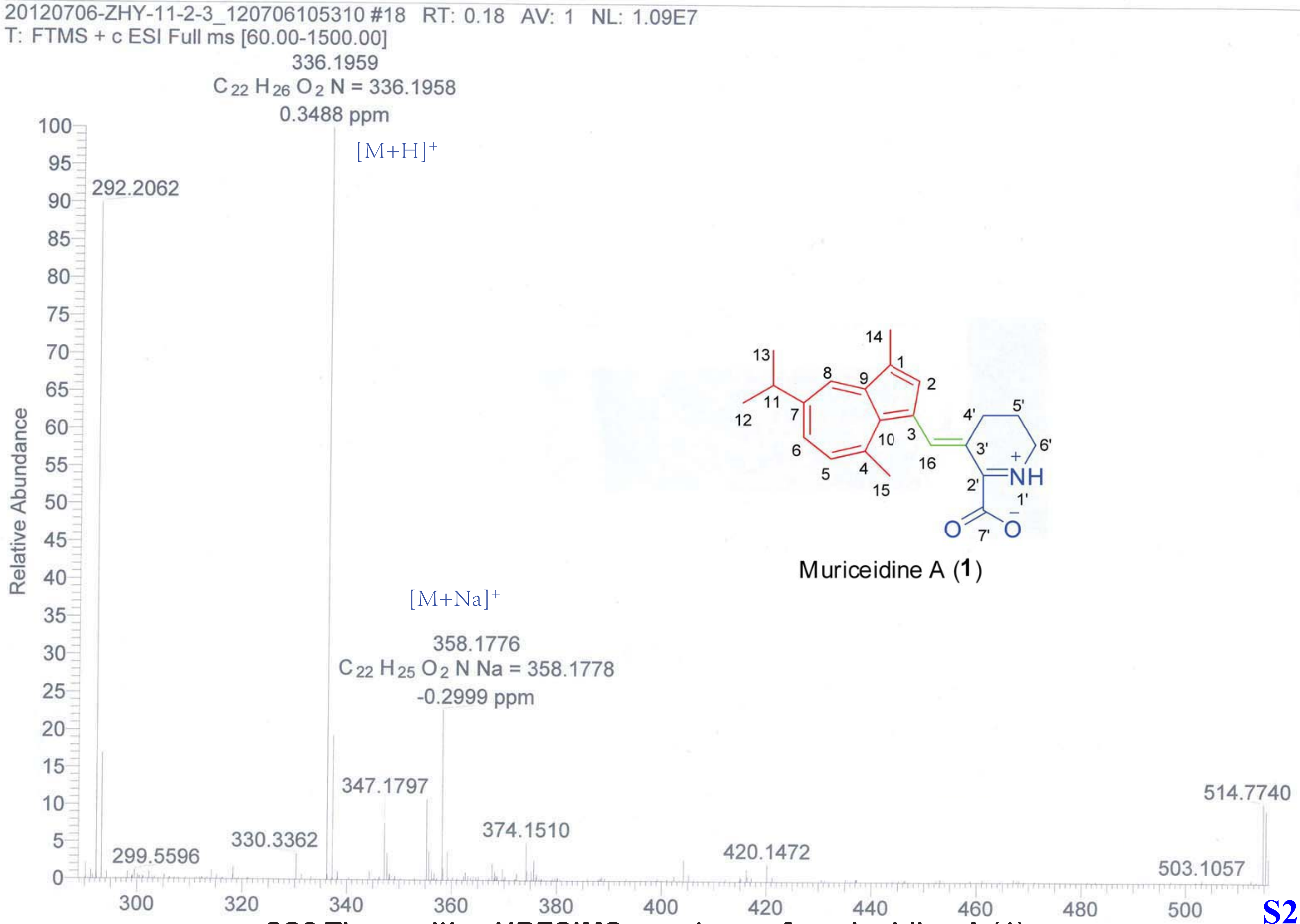

SS2 The positive HRESIMS spectrum of muriceidine A (1)

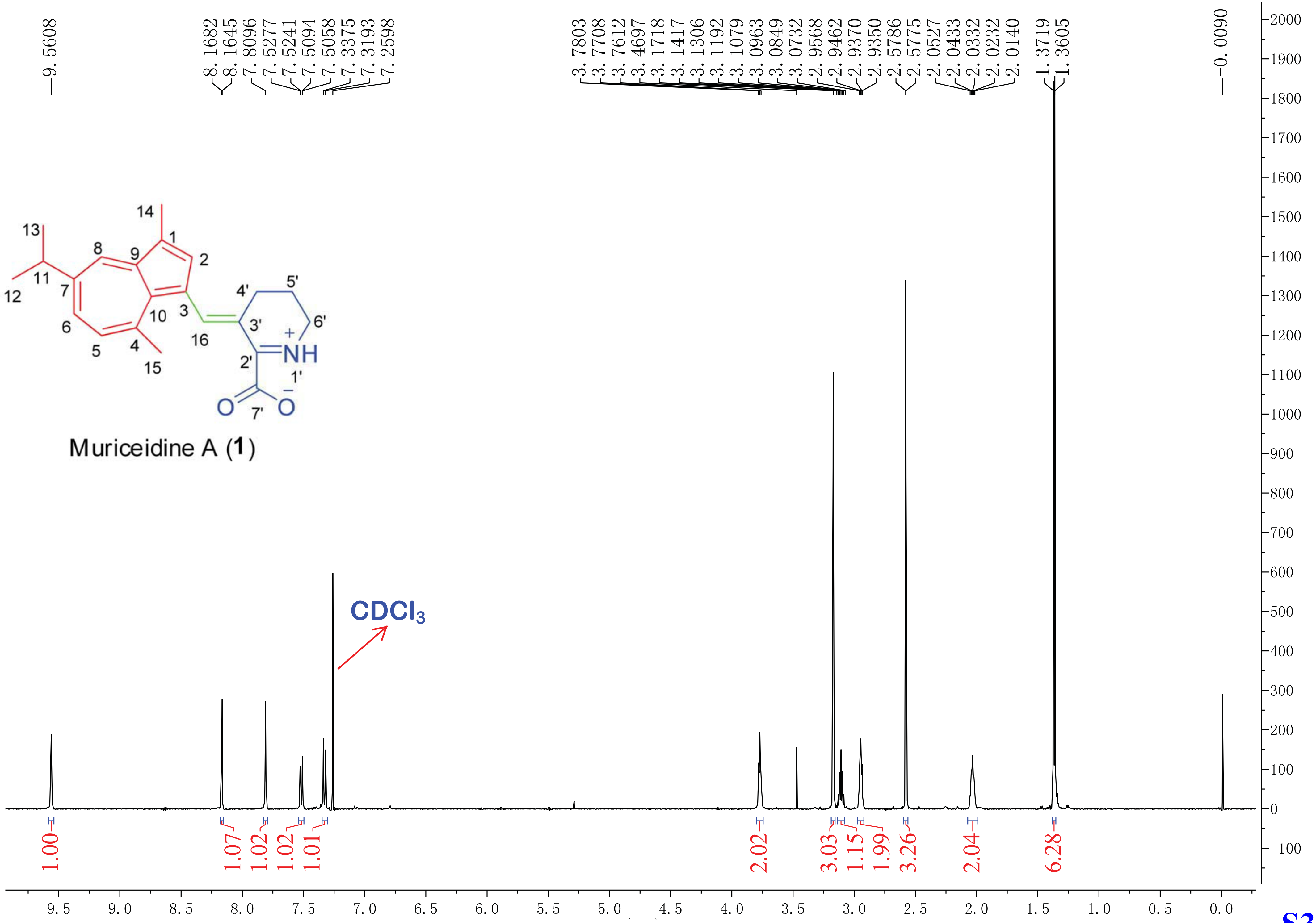

SS3 ^1H NMR (600 MHz, $CDCl_3$) spectrum of muriceidine A (1)

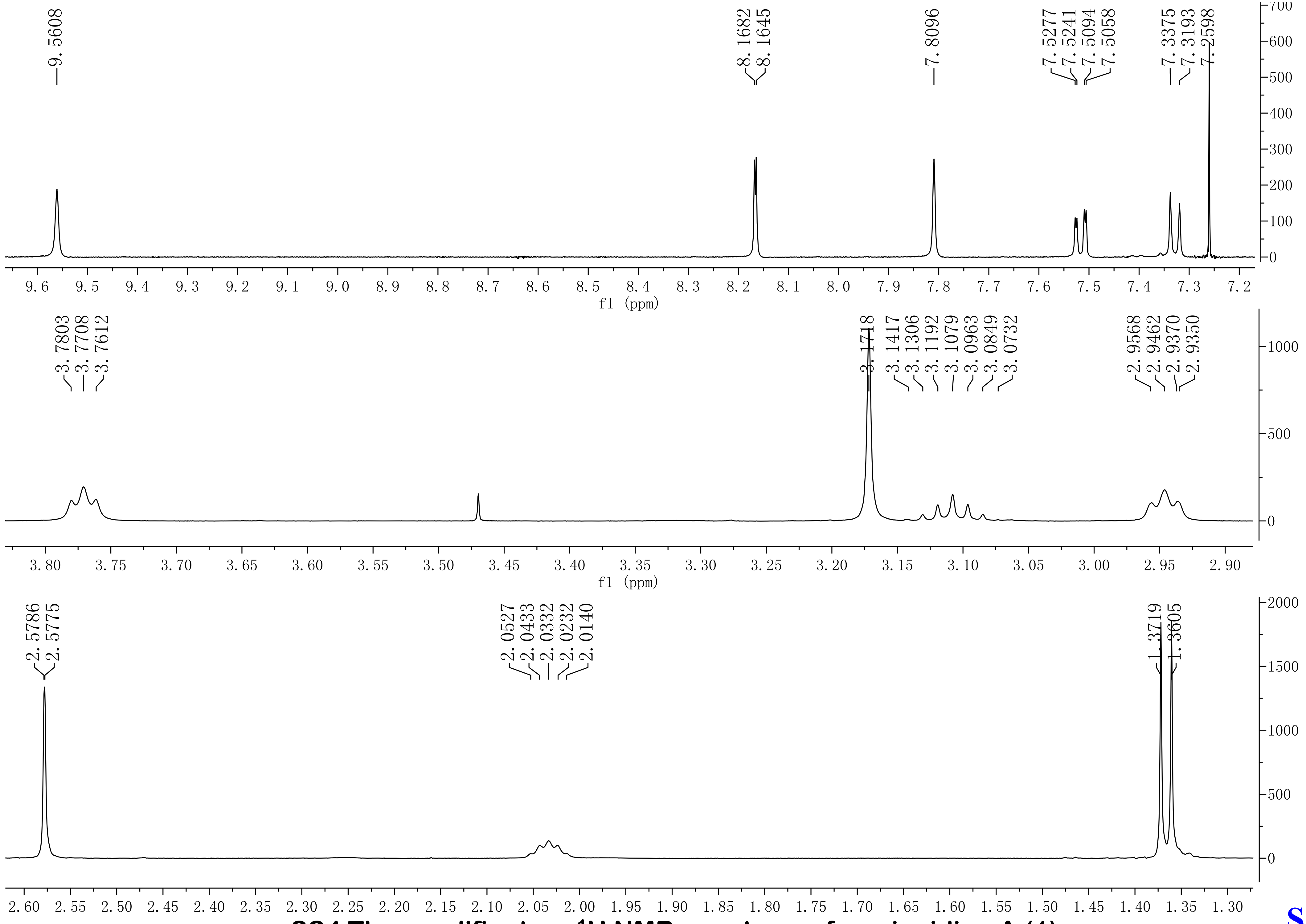

SS4 The amplificatory ¹H NMR spectrum of muriceidine A (1)

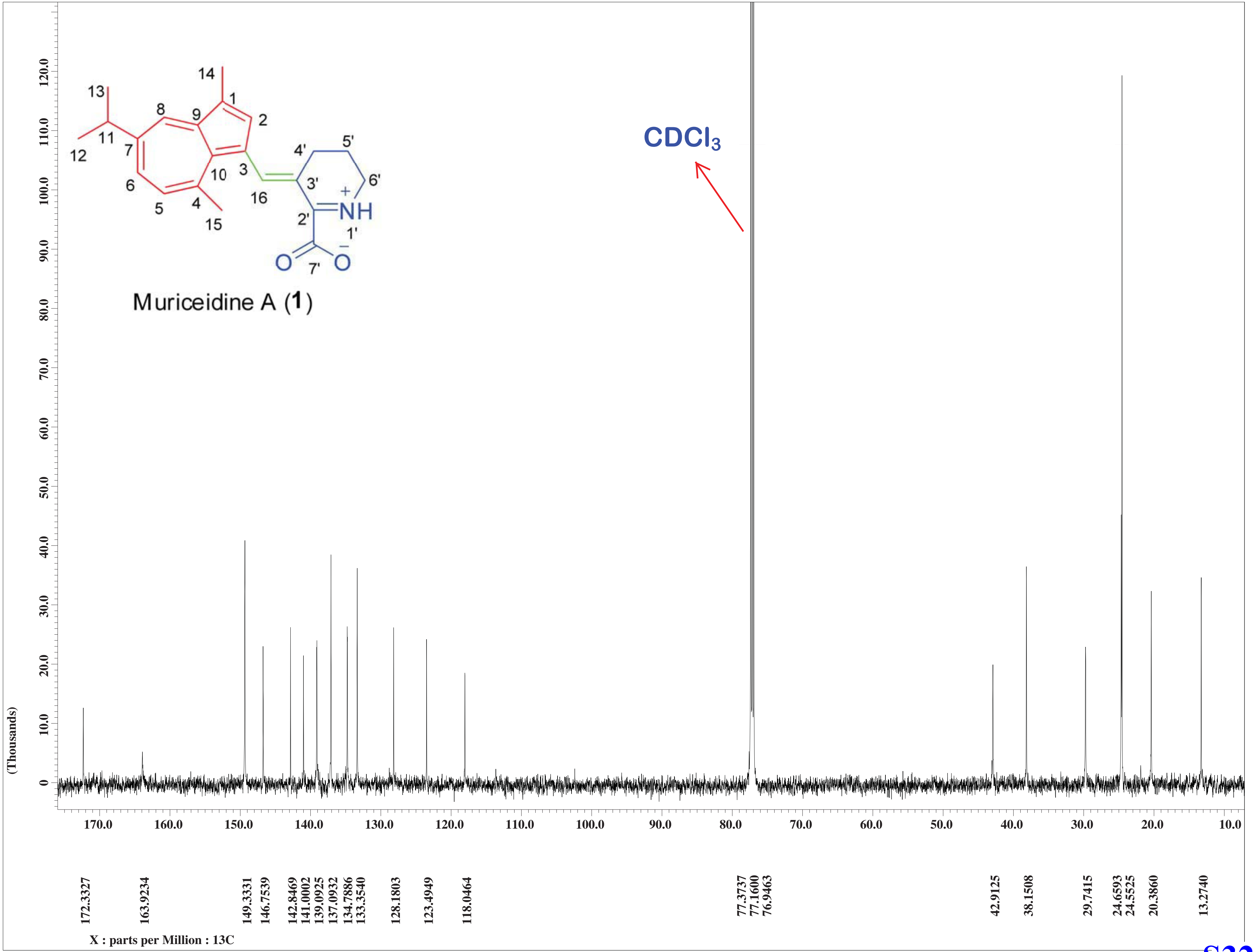

SS5 ^{13}C NMR (150 MHz, $CDCl_3$) spectrum of muriceidine A (1)

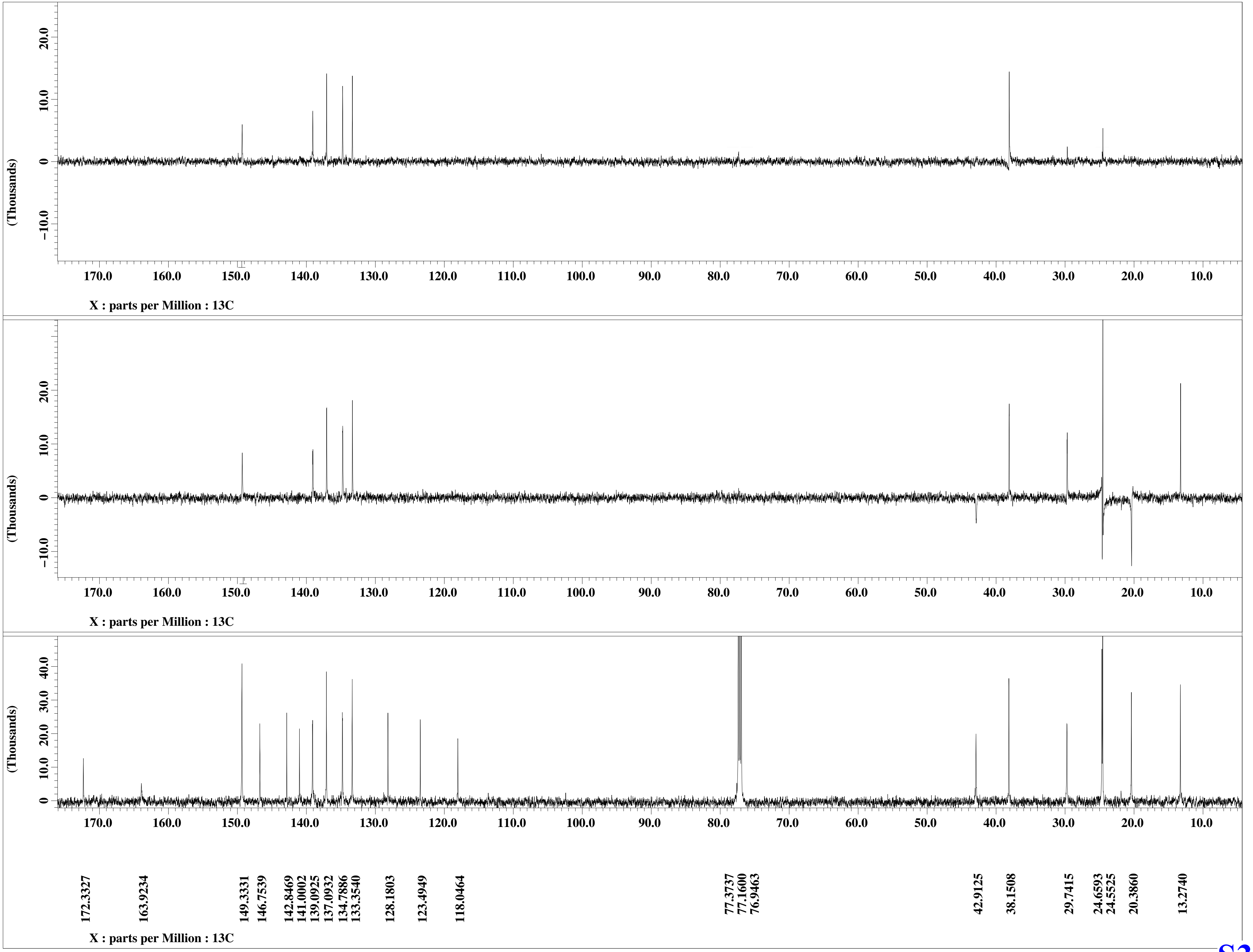

SS6 DEPT spectrum of muriceidine A (1)

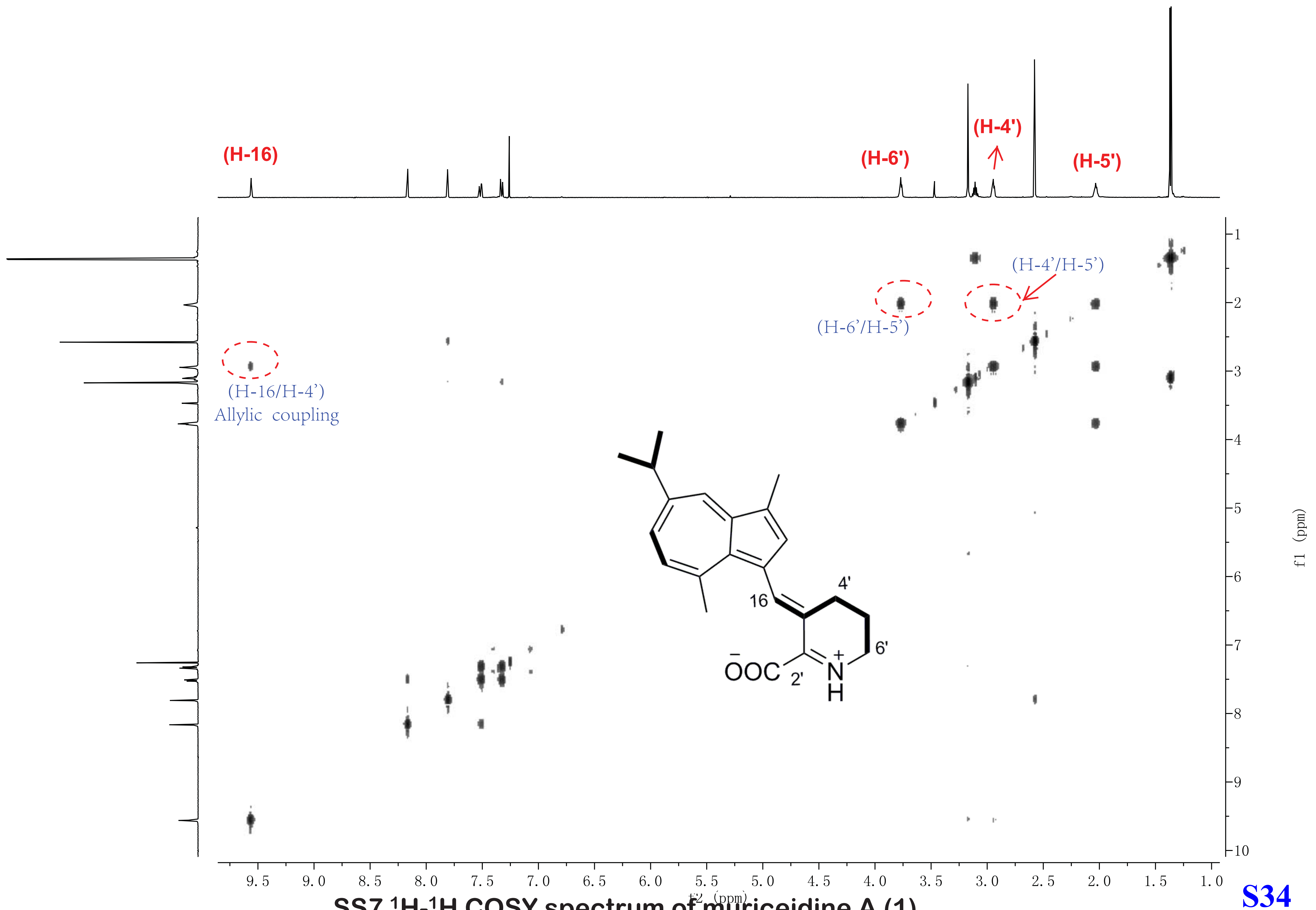

SS7 ^{1}H-^{1}H COSY spectrum of muriceidine A (1)

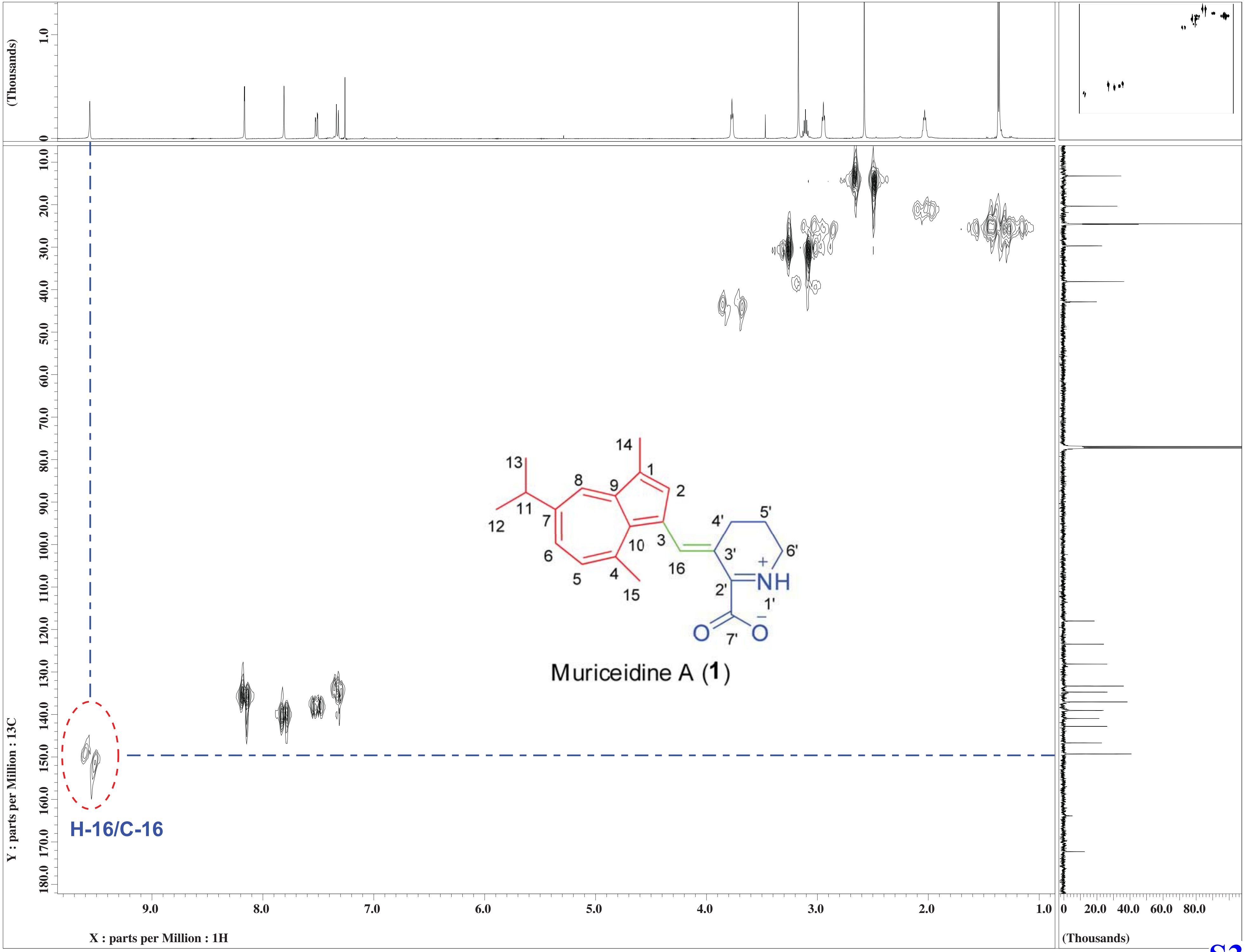

SS8 HMQC spectrum of muriceidine A (1)

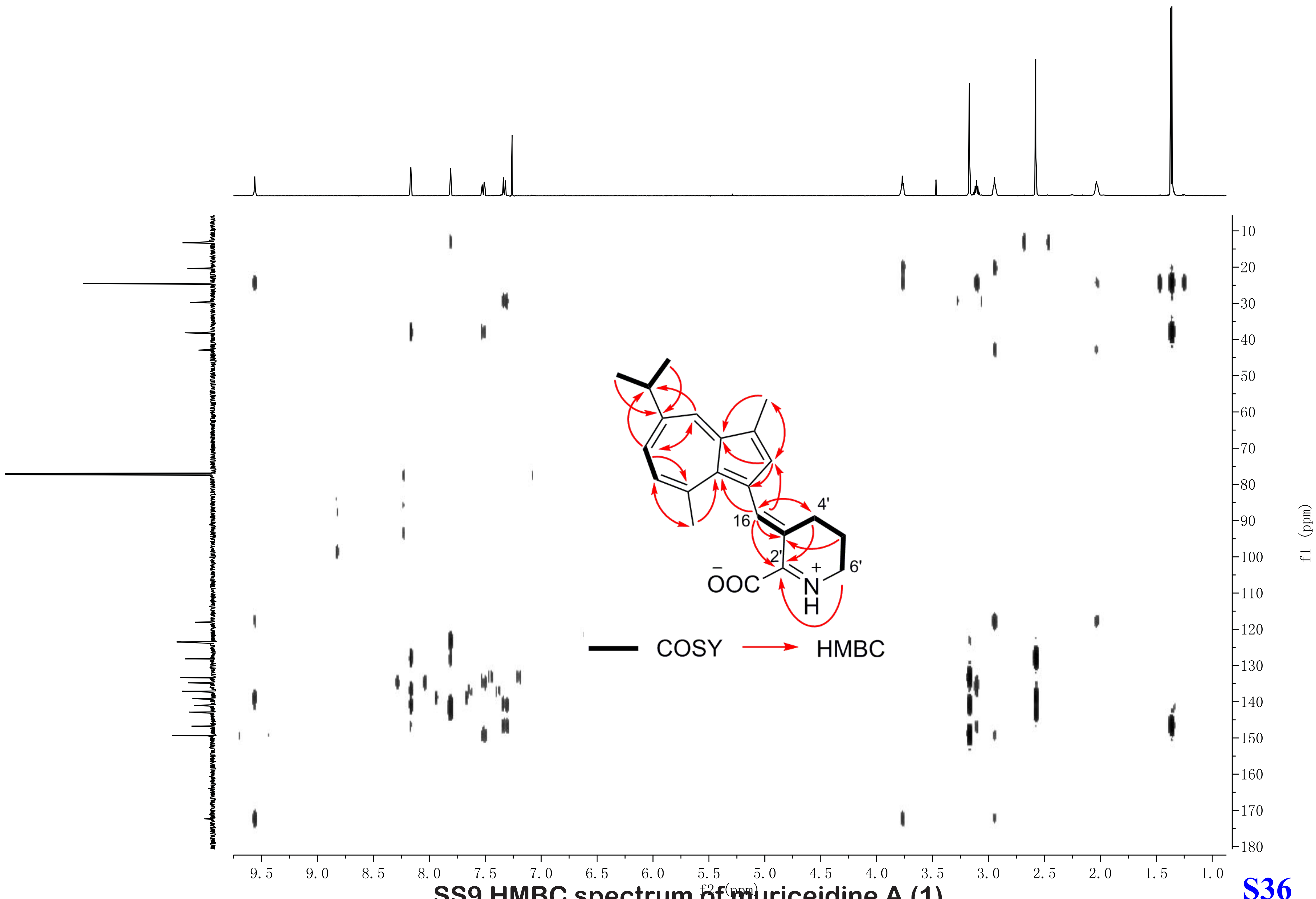

SS9 HMBC spectrum of muriceidine A (1)

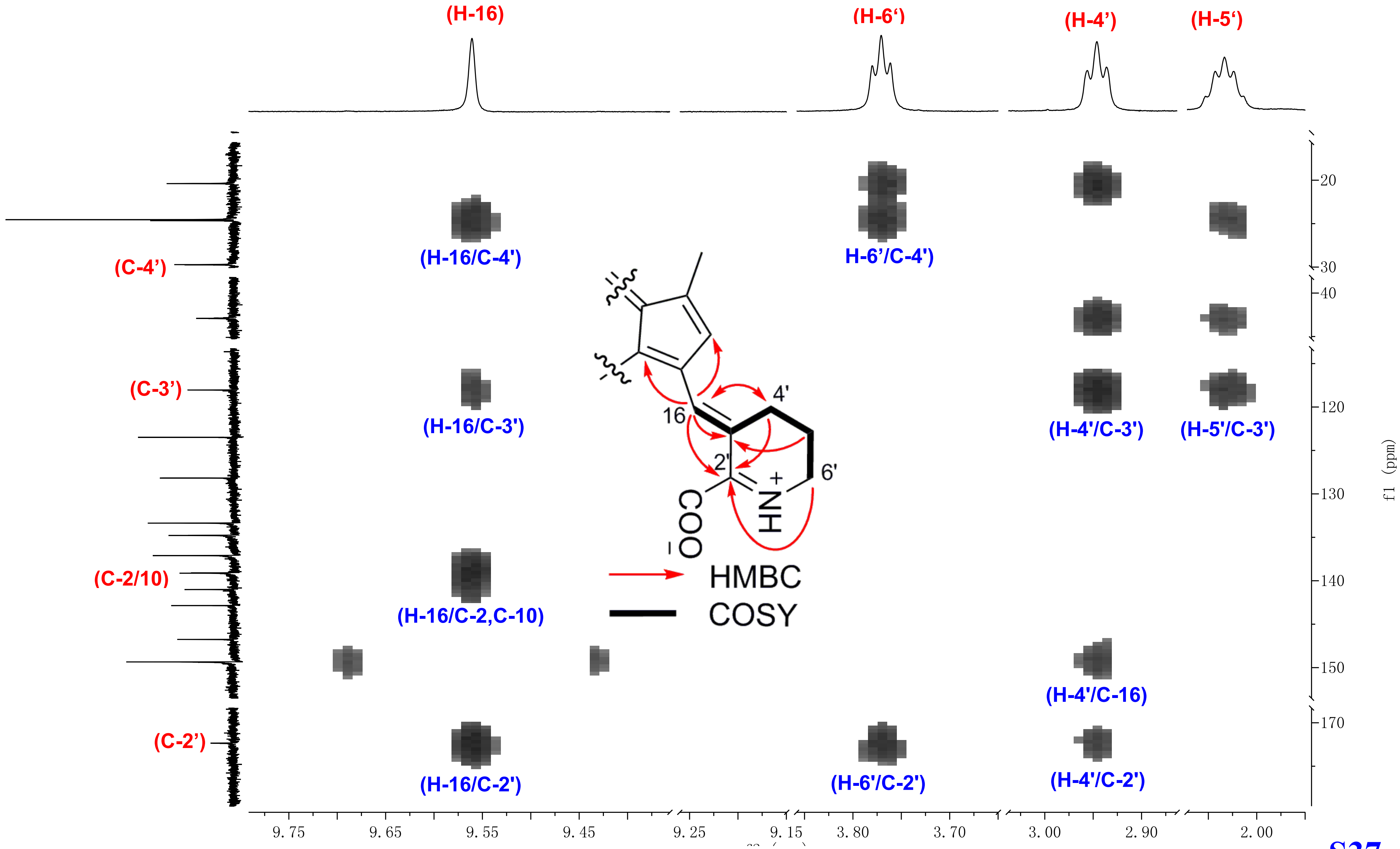

SS10 The key amplificatory HMBC spectrum of muriceidine A (1)

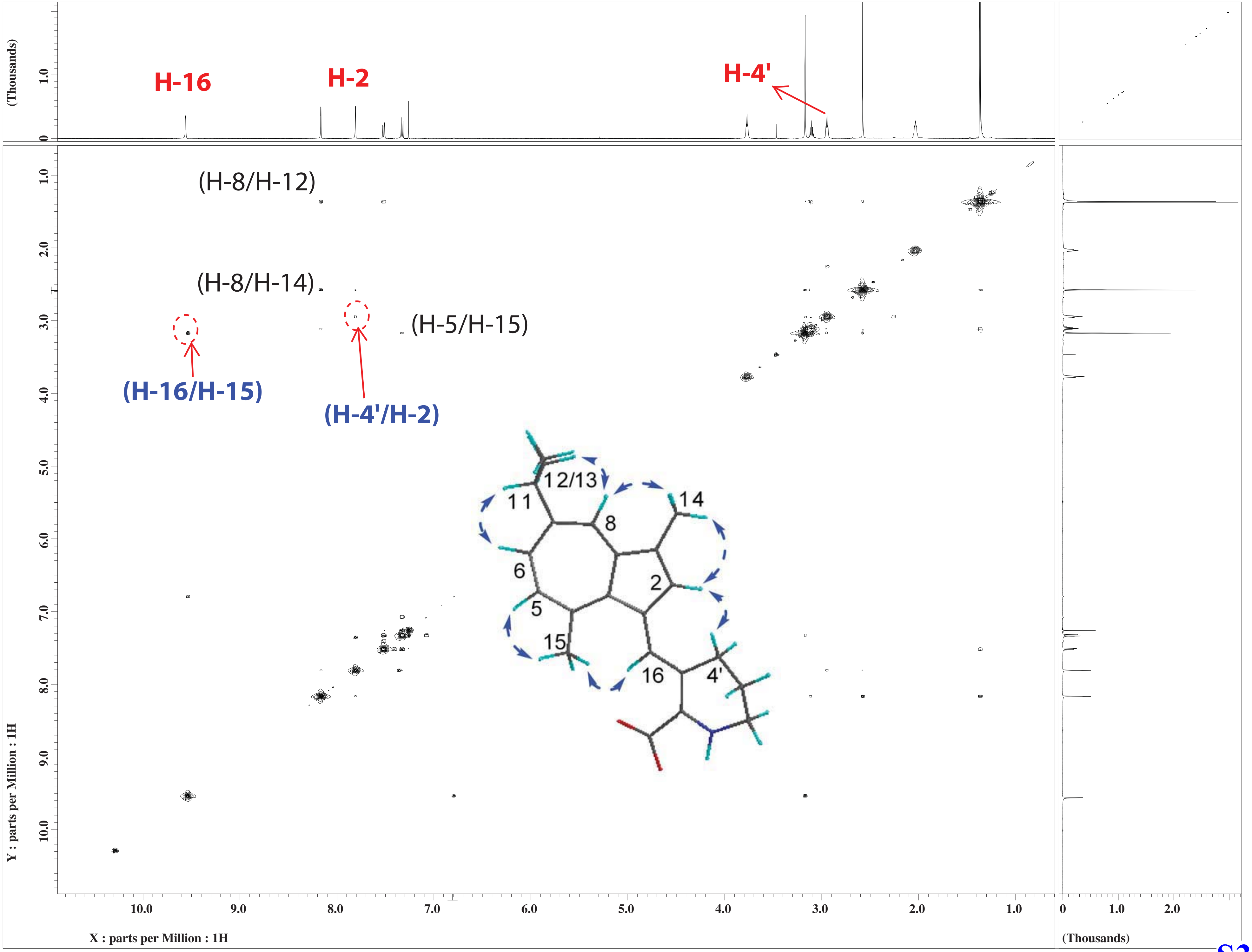

SS11 NOESY spectrum of muriceidine A (1)

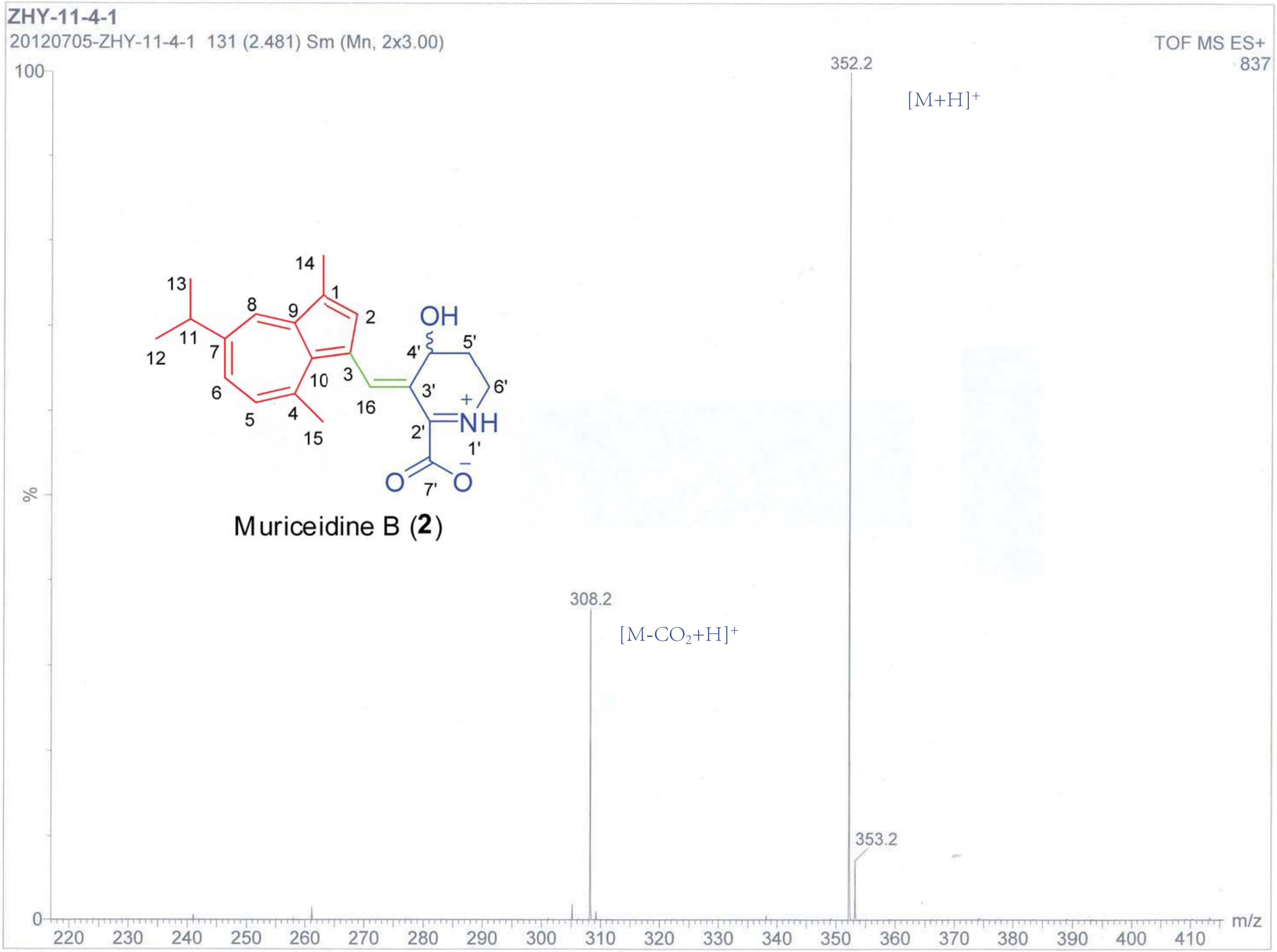

SS12 The positive ESIMS spectrum of muriceidine B (2)

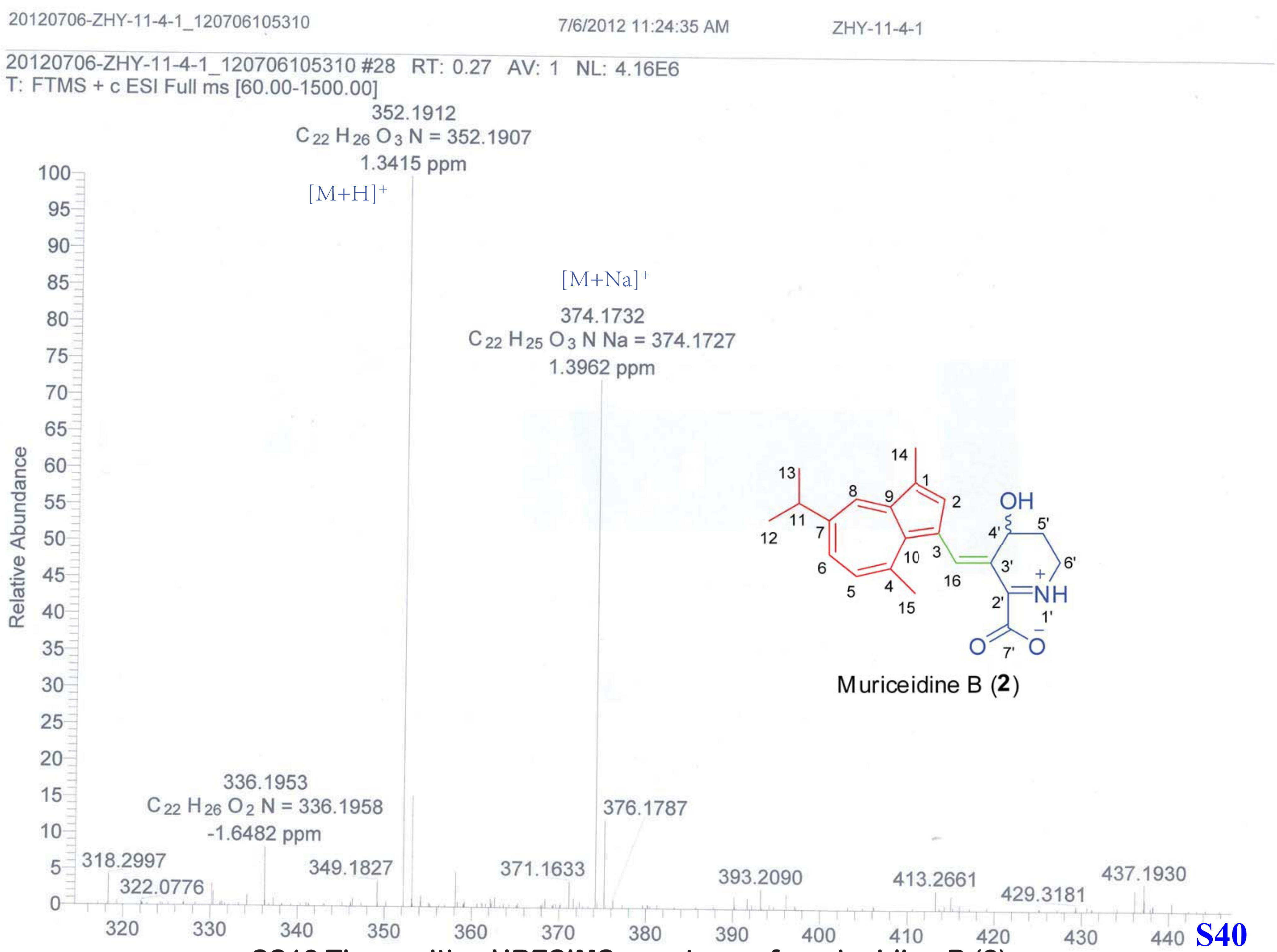

SS13 The positive HRESIMS spectrum of muriceidine B (2)

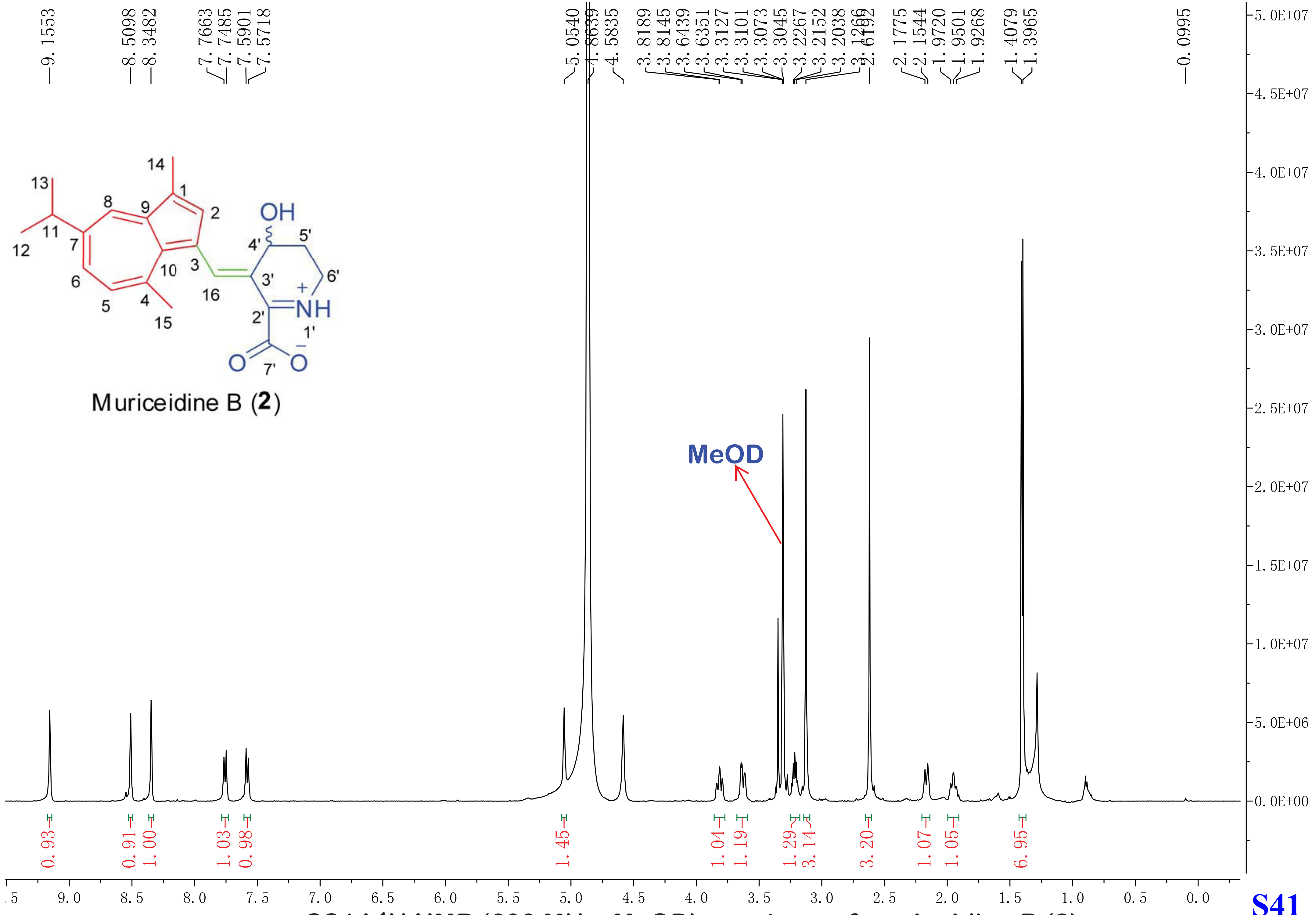

SS14 ^{1}H NMR (600 MHz, MeOD) spectrum of muriceidine B (2)

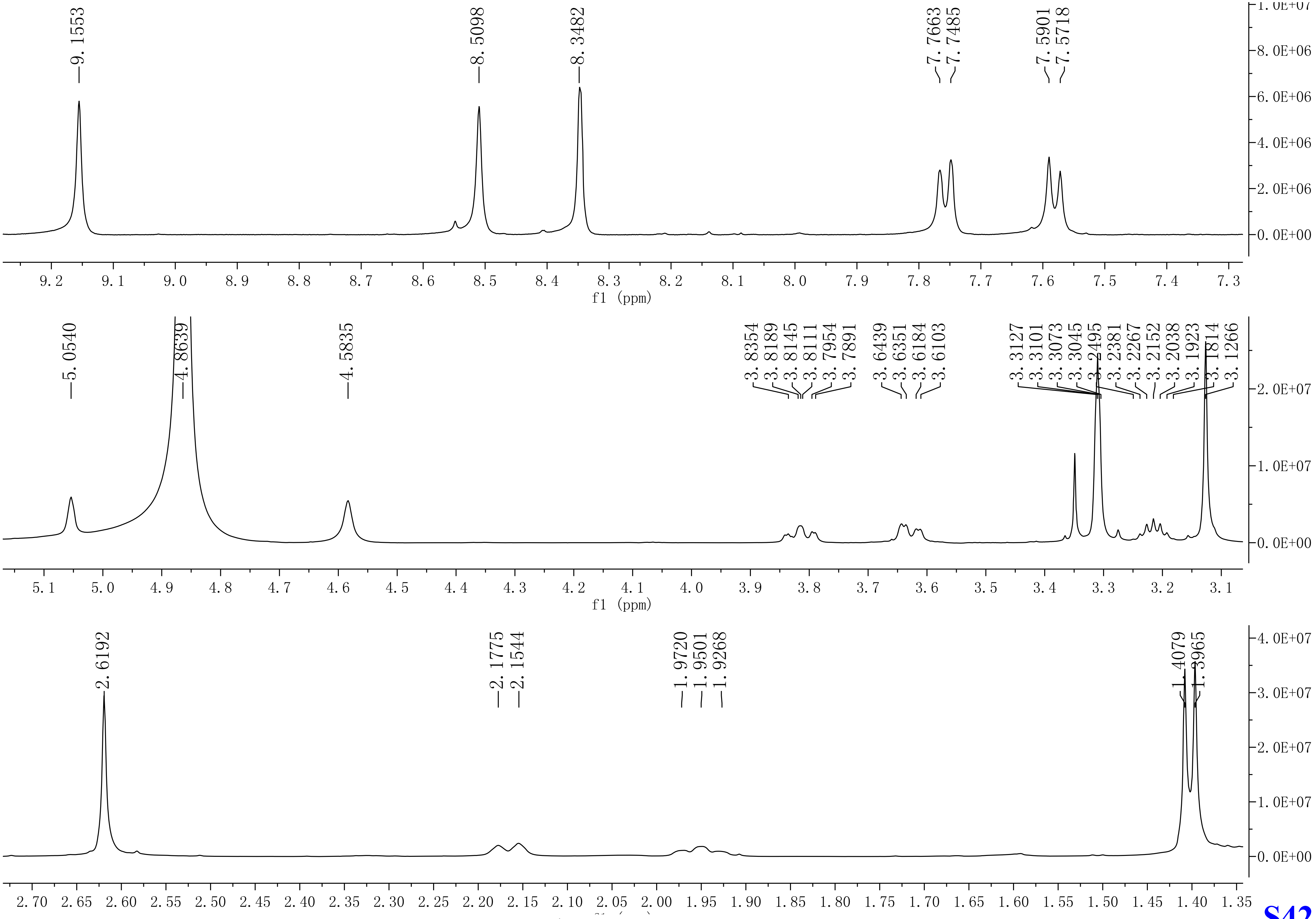

SS15 The amplificatory ¹H NMR spectrum of muriceidine B (2)

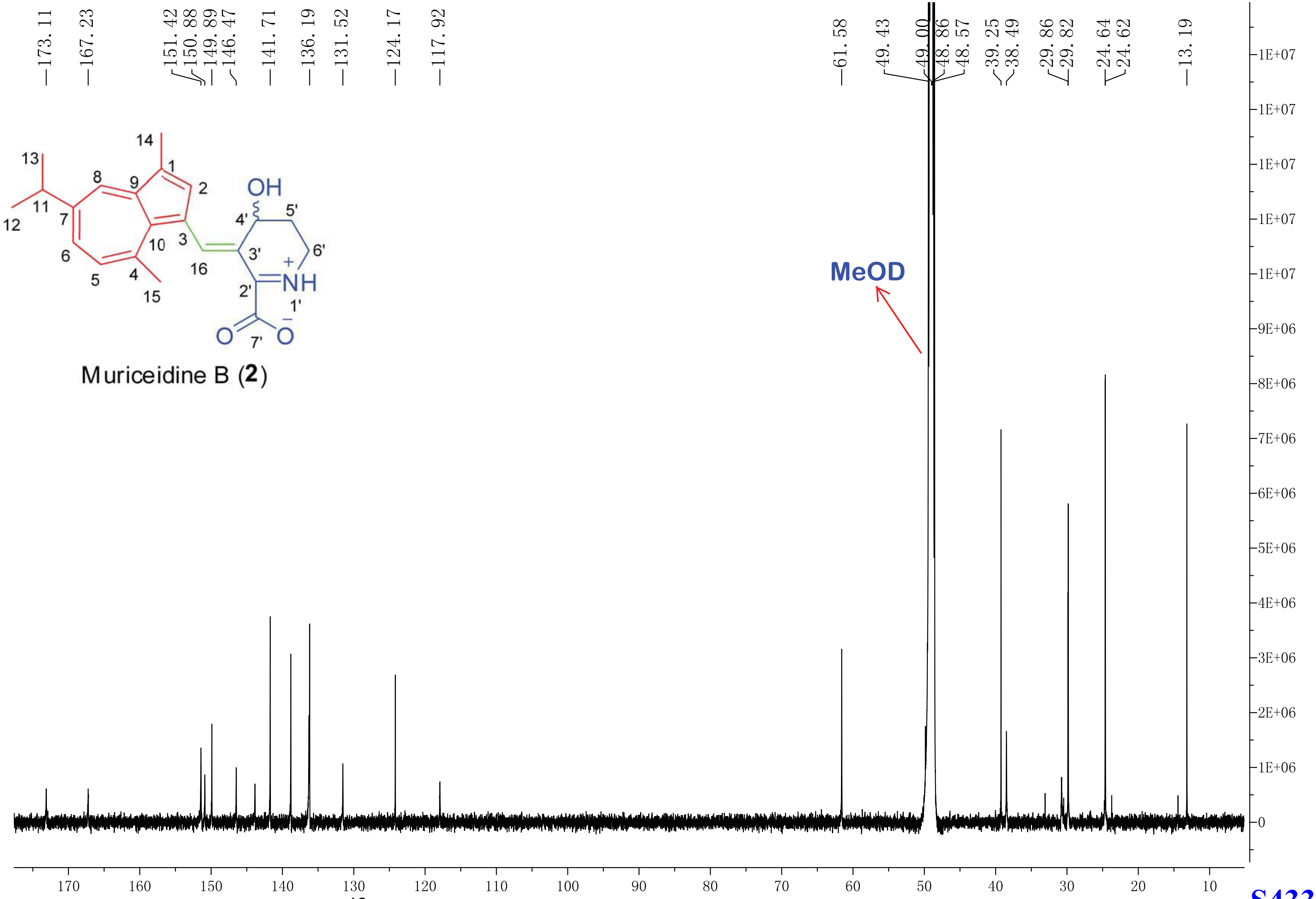

SS16 ^{13}C NMR (600 MHz, MeOD) spectrum of muriceidine B (2)

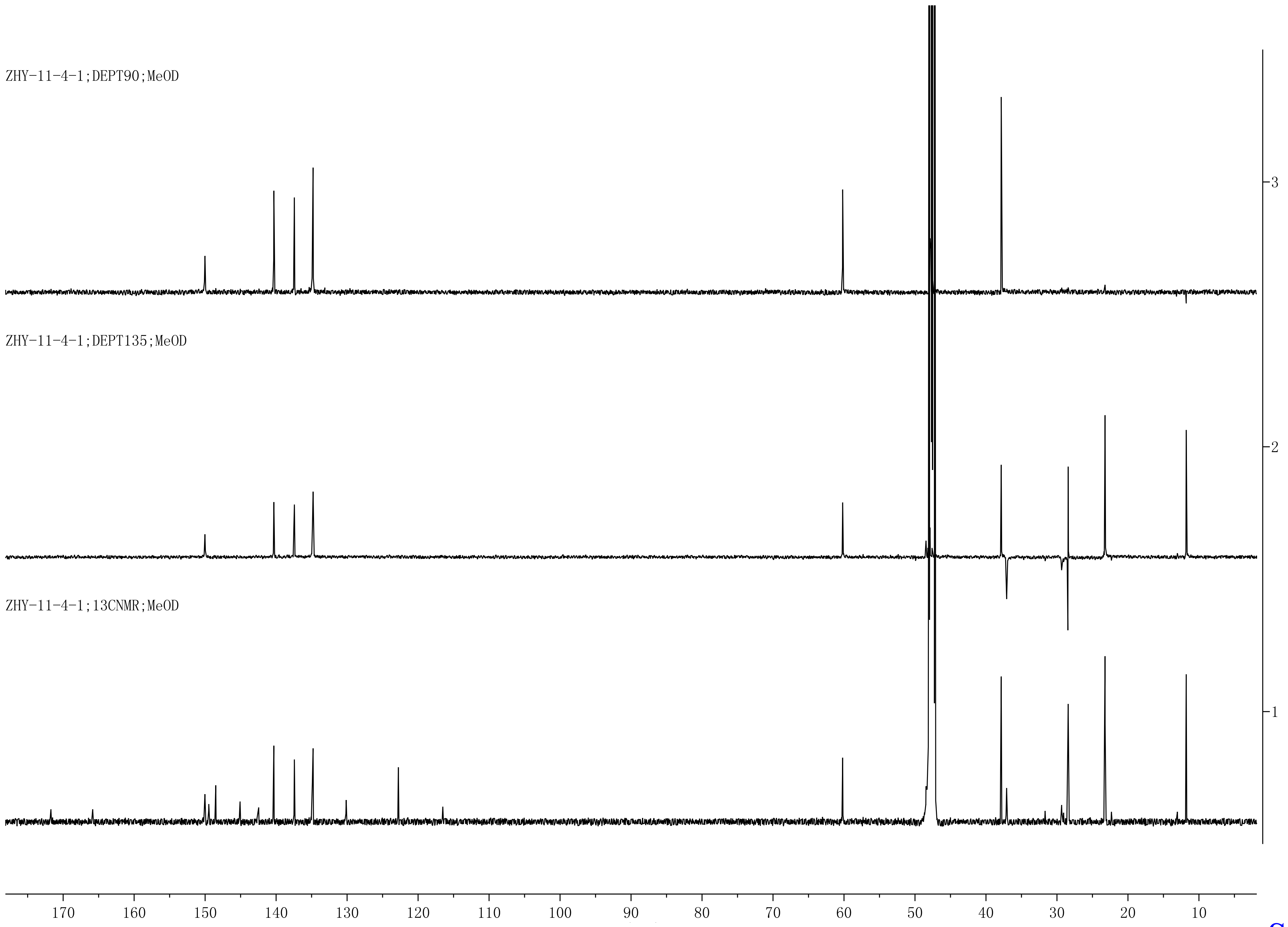

SS17 DEPT spectrum of muriceidine B (2)

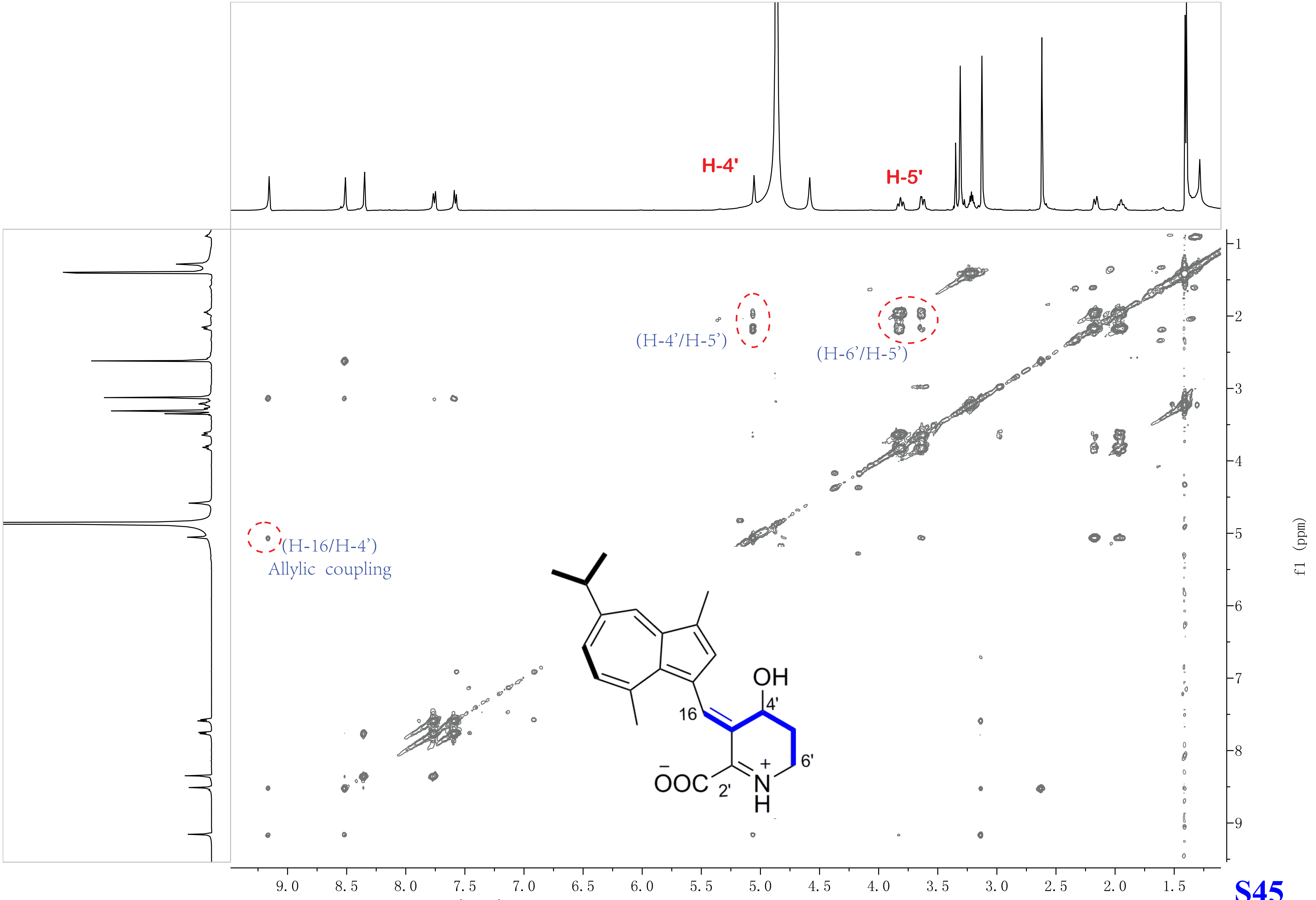

SS18 ^{1}H-^{1}H COSY spectrum of muriceidine B (2)

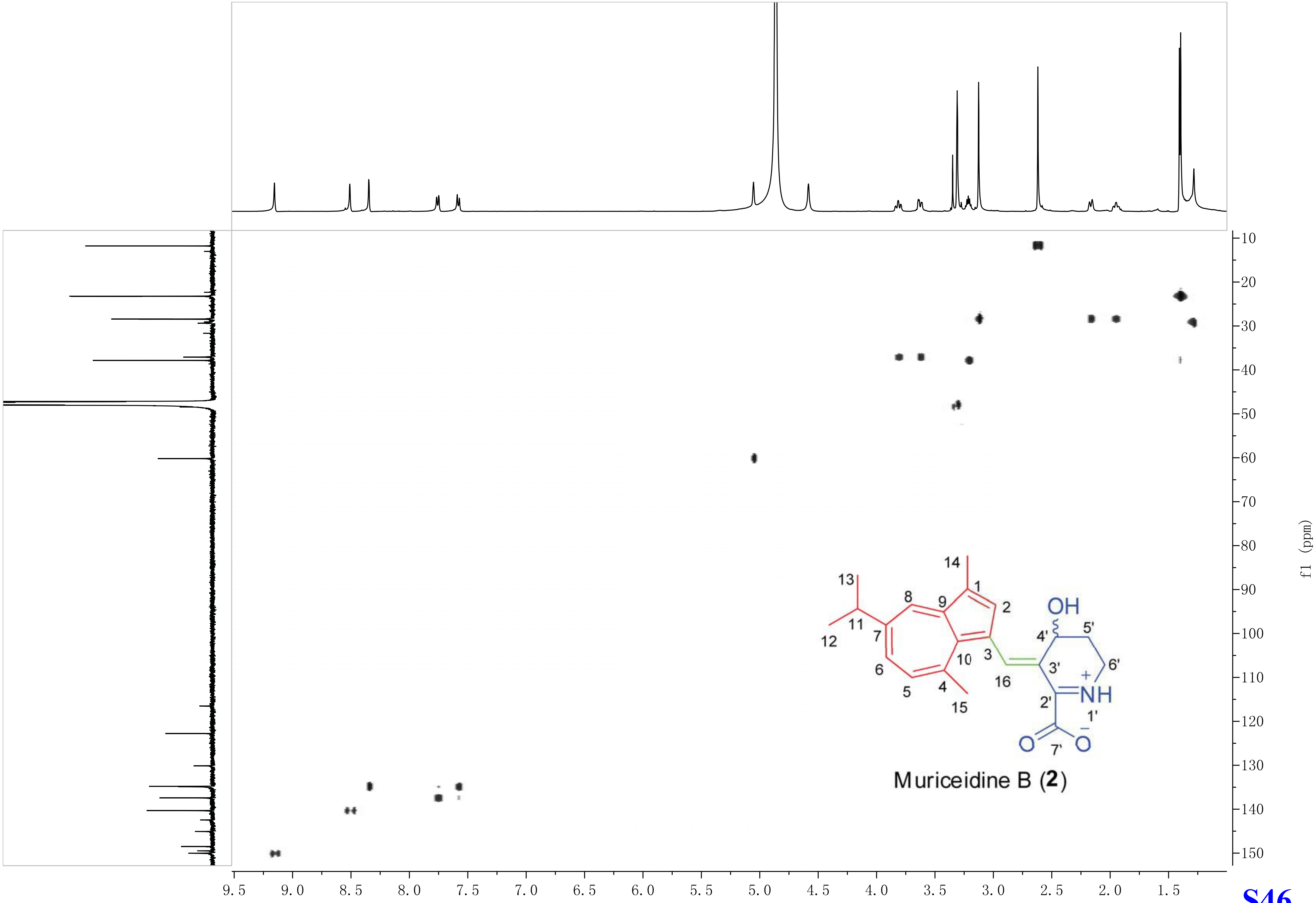

SS19 HMQC spectrum of muriceidine B (2)

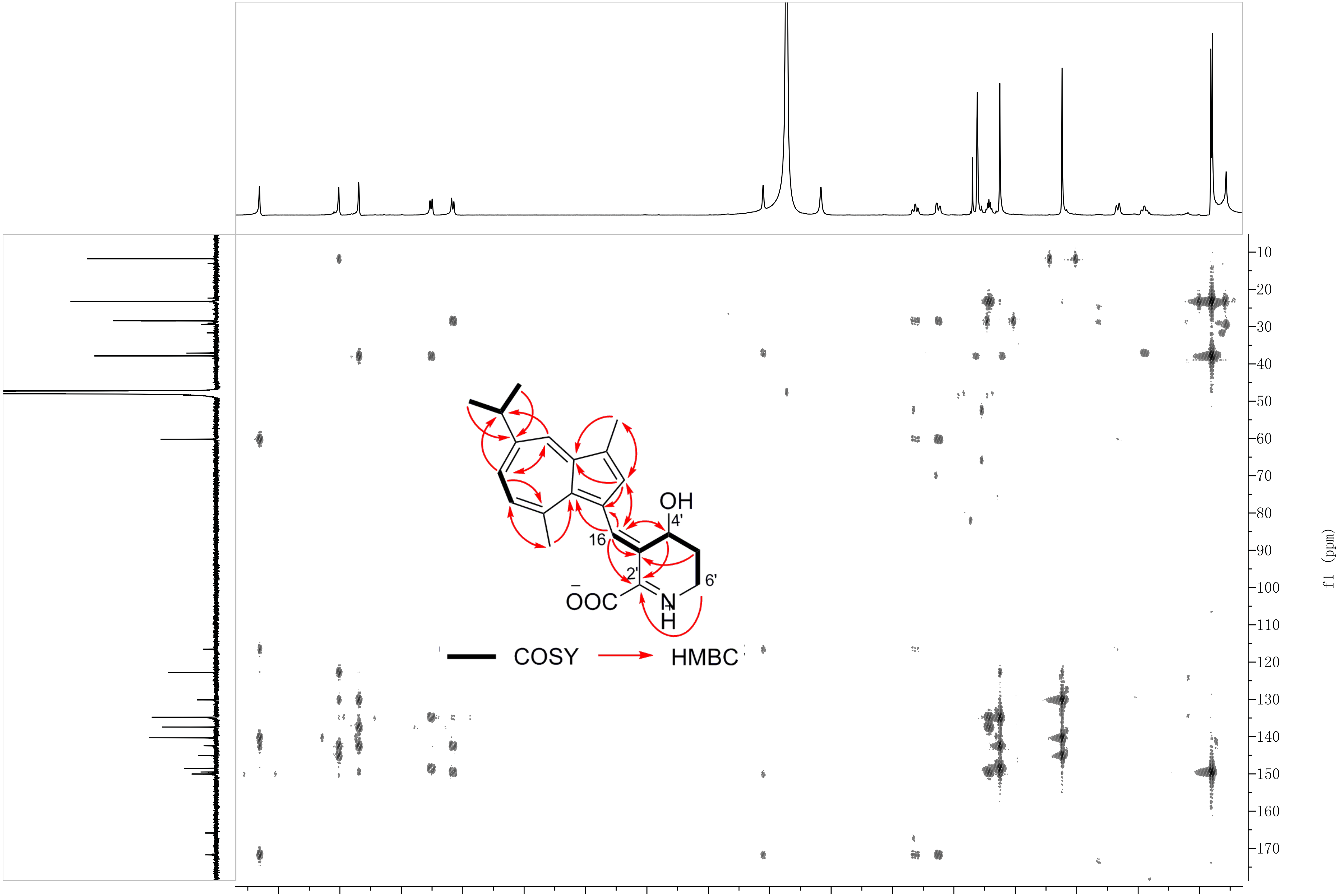

SS20 HMBC spectrum of muriceidine B (2)

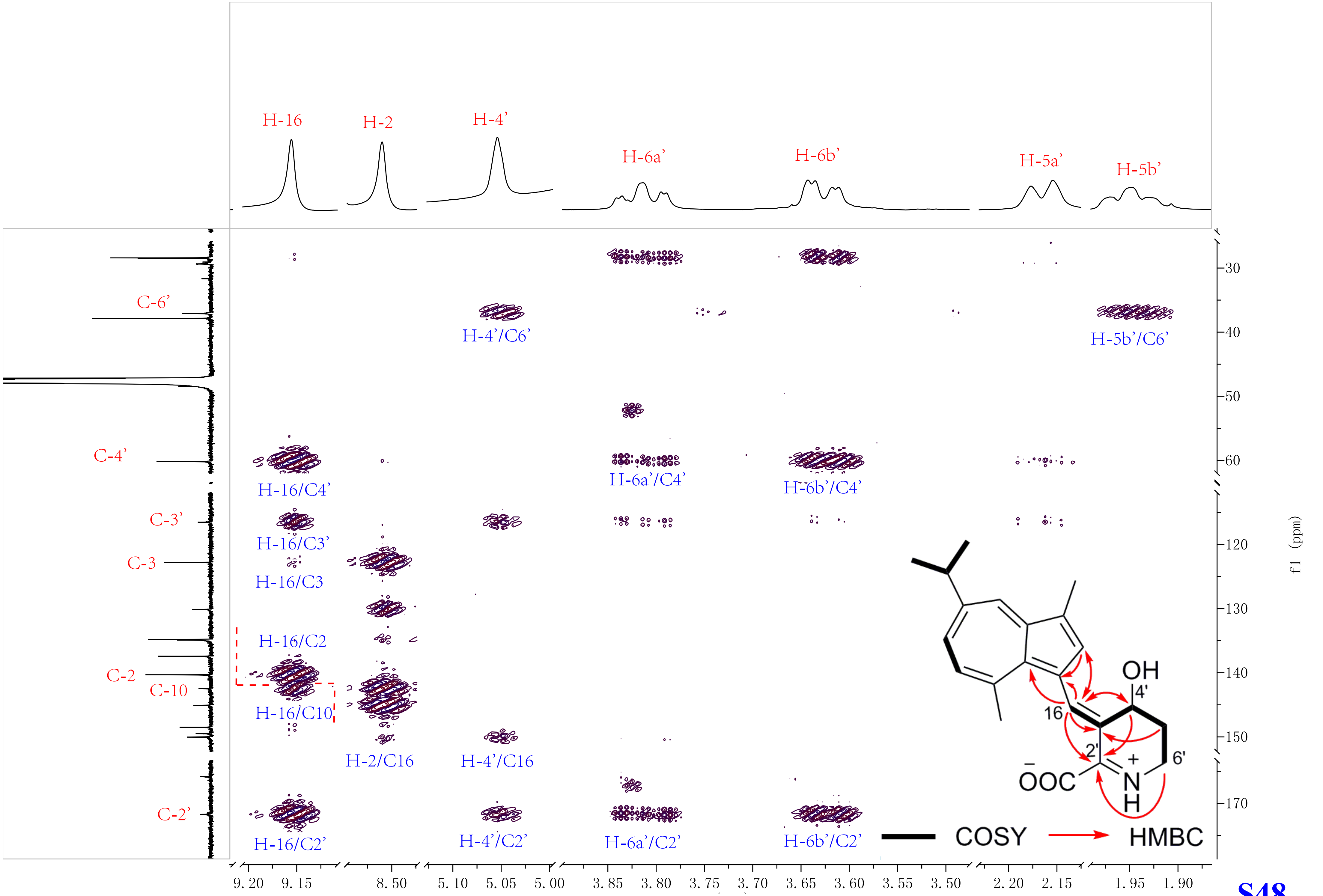

SS21 key amplificatory HMBC spectrum of muriceidine B (2)

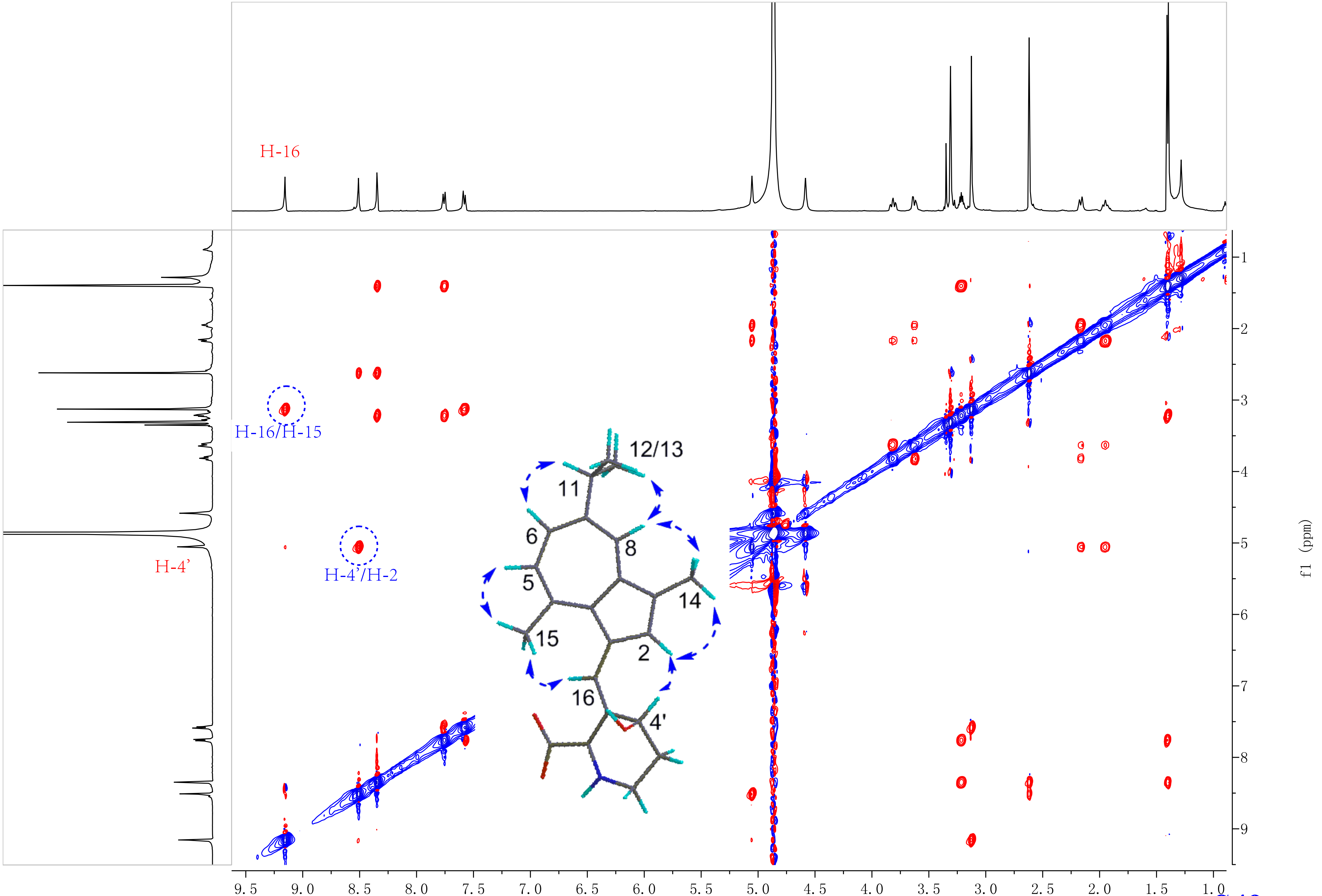

SS22 NOESY spectrum of muriceidine B (2)

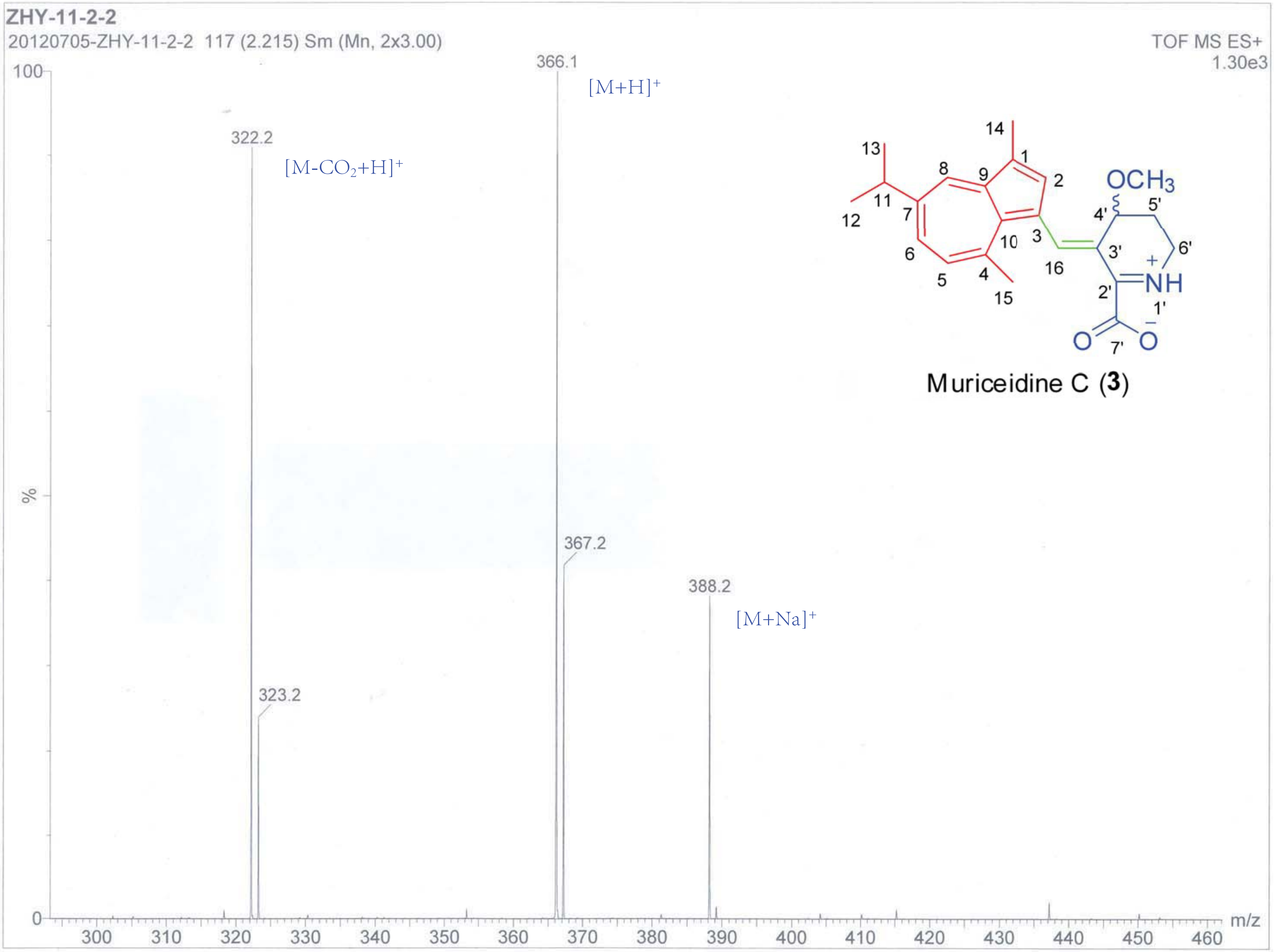

SS23 The positive ESIMS spectrum of muriceidine C (3)

20120706-ZHY-11-2-2_120706105310 7/6/2012 11:25:52 AM ZHY-11-2-2

20120706-ZHY-11-2-2_120706105310 #27 RT: 0.25 AV: 1 NL: 6.23E6
T: FTMS + c ESI Full ms [60.00-1500.00]

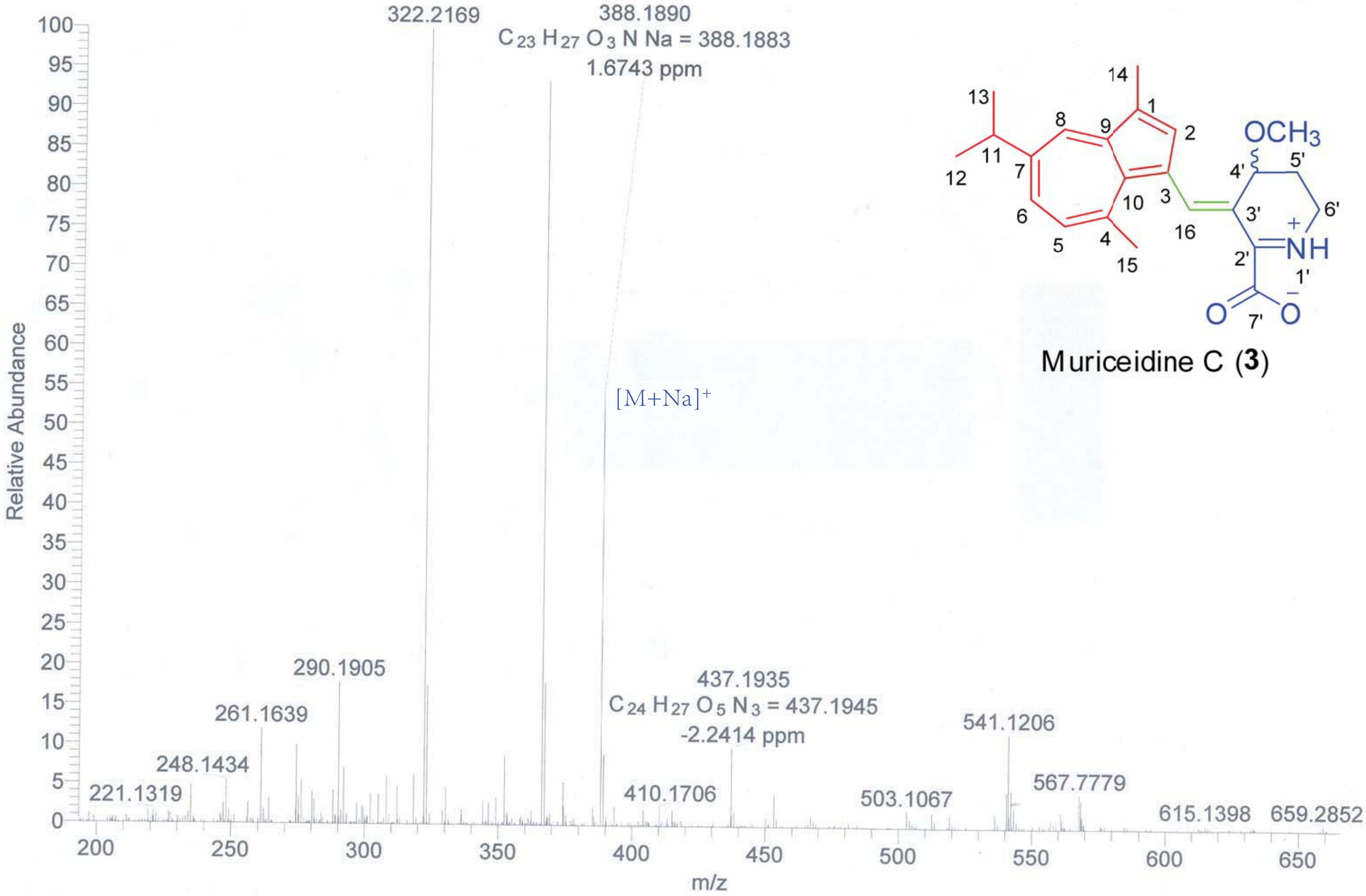

SS24 The positive HRESIMS spectrum of muriceidine C (3)

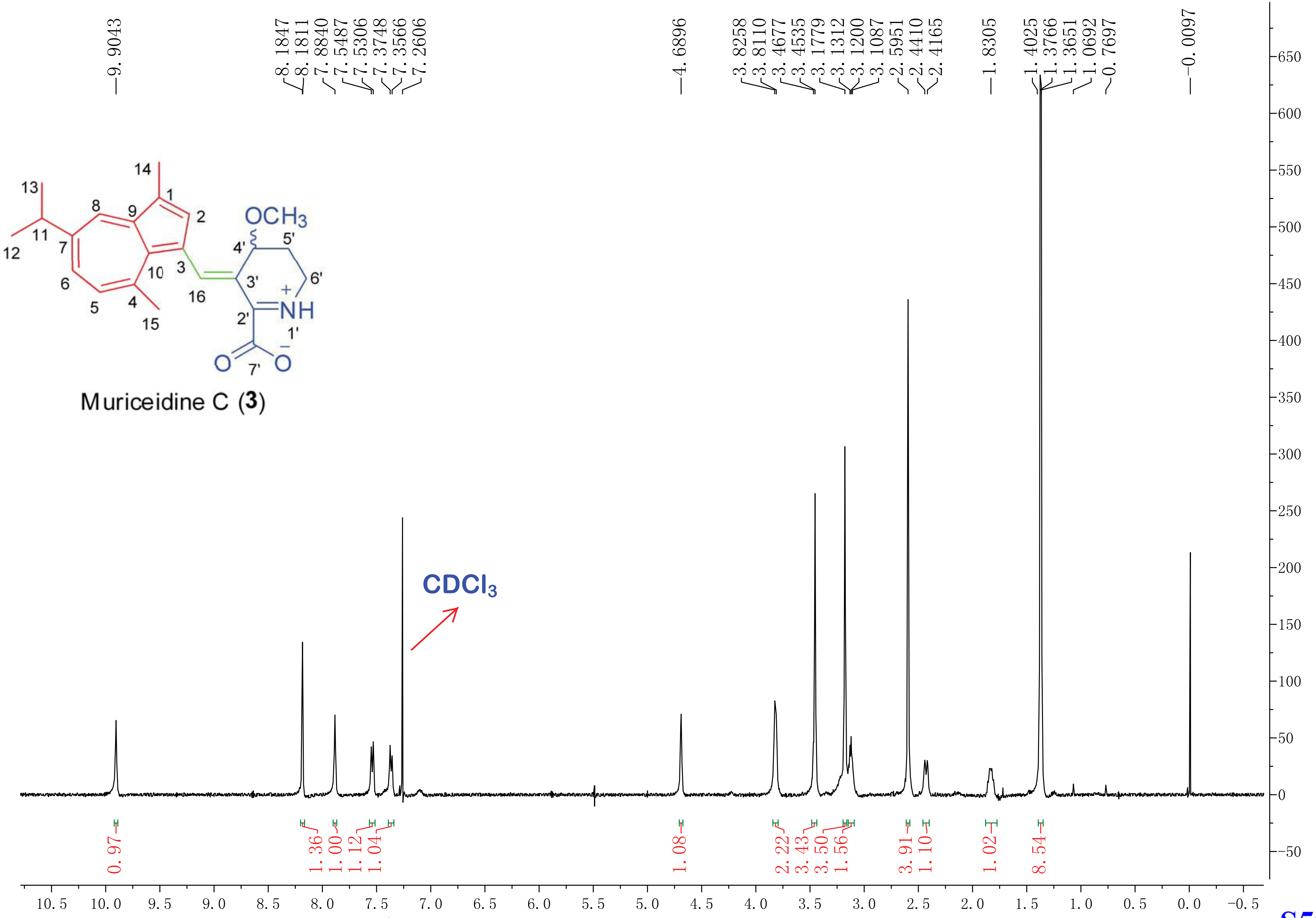

SS25 ^1H NMR (600 MHz, $CDCl_3$) spectrum of muriceidine C (3)

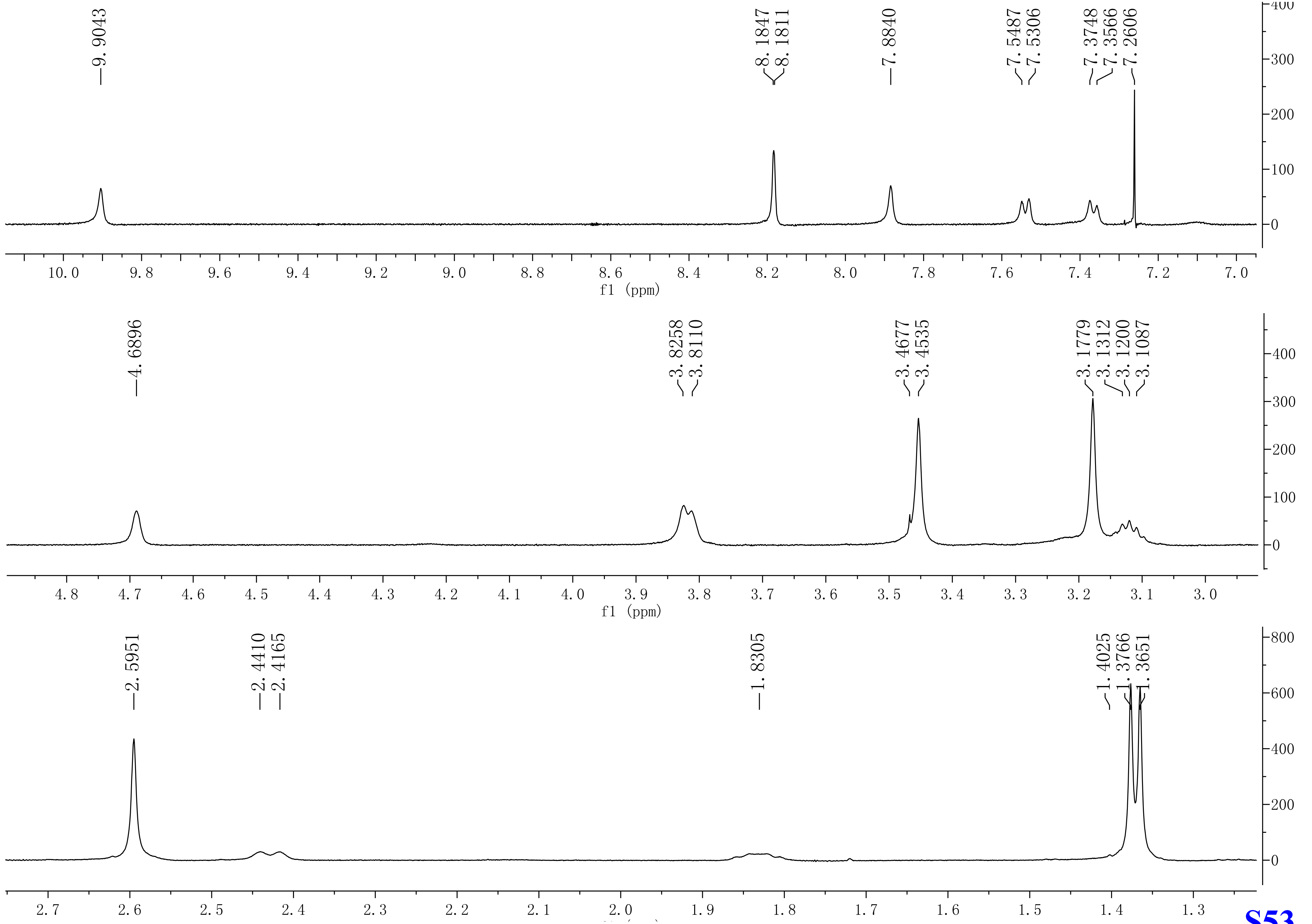

SS26 The amplificatory ^{1}H NMR spectrum of muriceidine C (3)

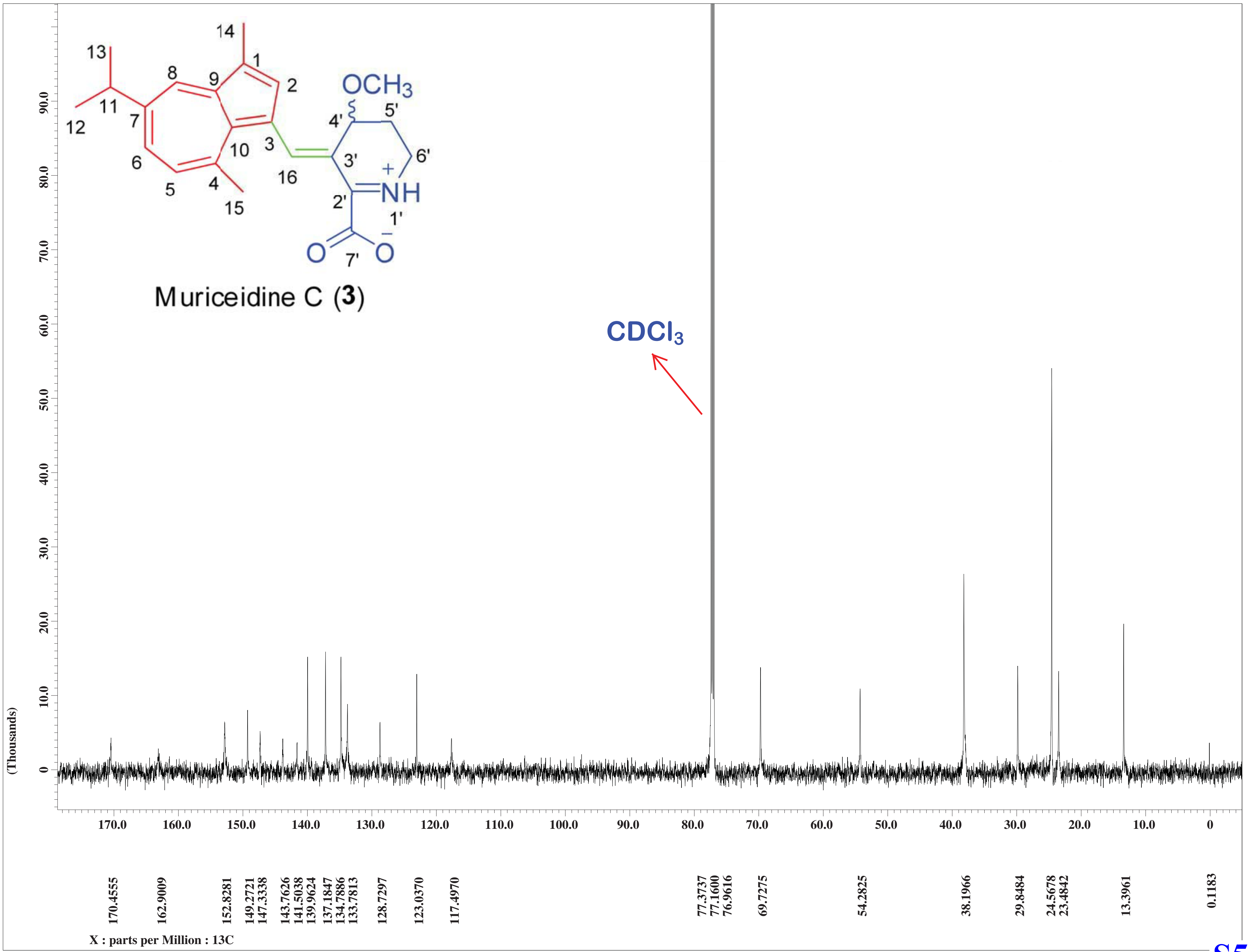

SS27 ^{13}C NMR (150 MHz, $CDCl_3$) spectrum of muriceidine C (3)

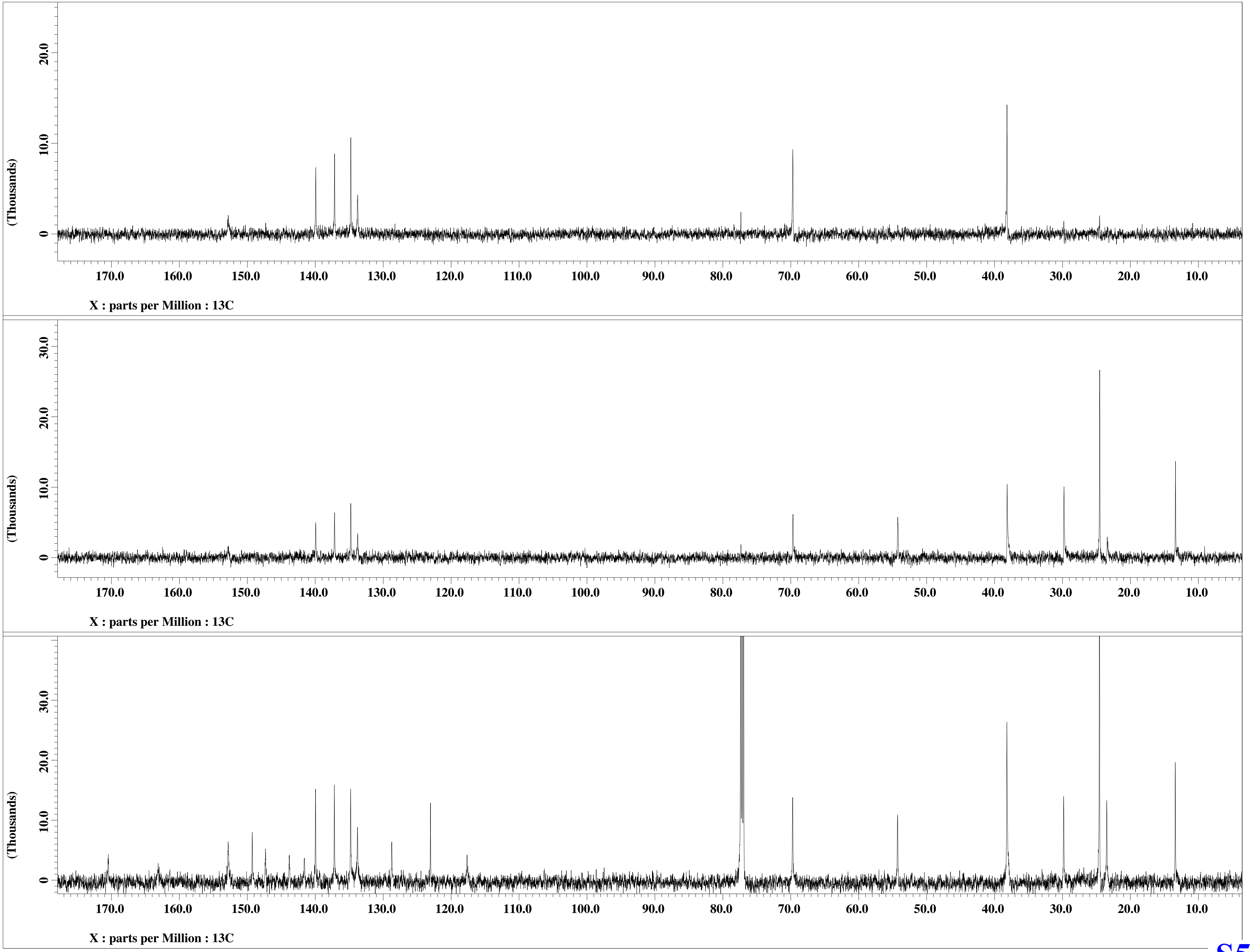

SS28 DEPT spectrum of muriceidine C (3)

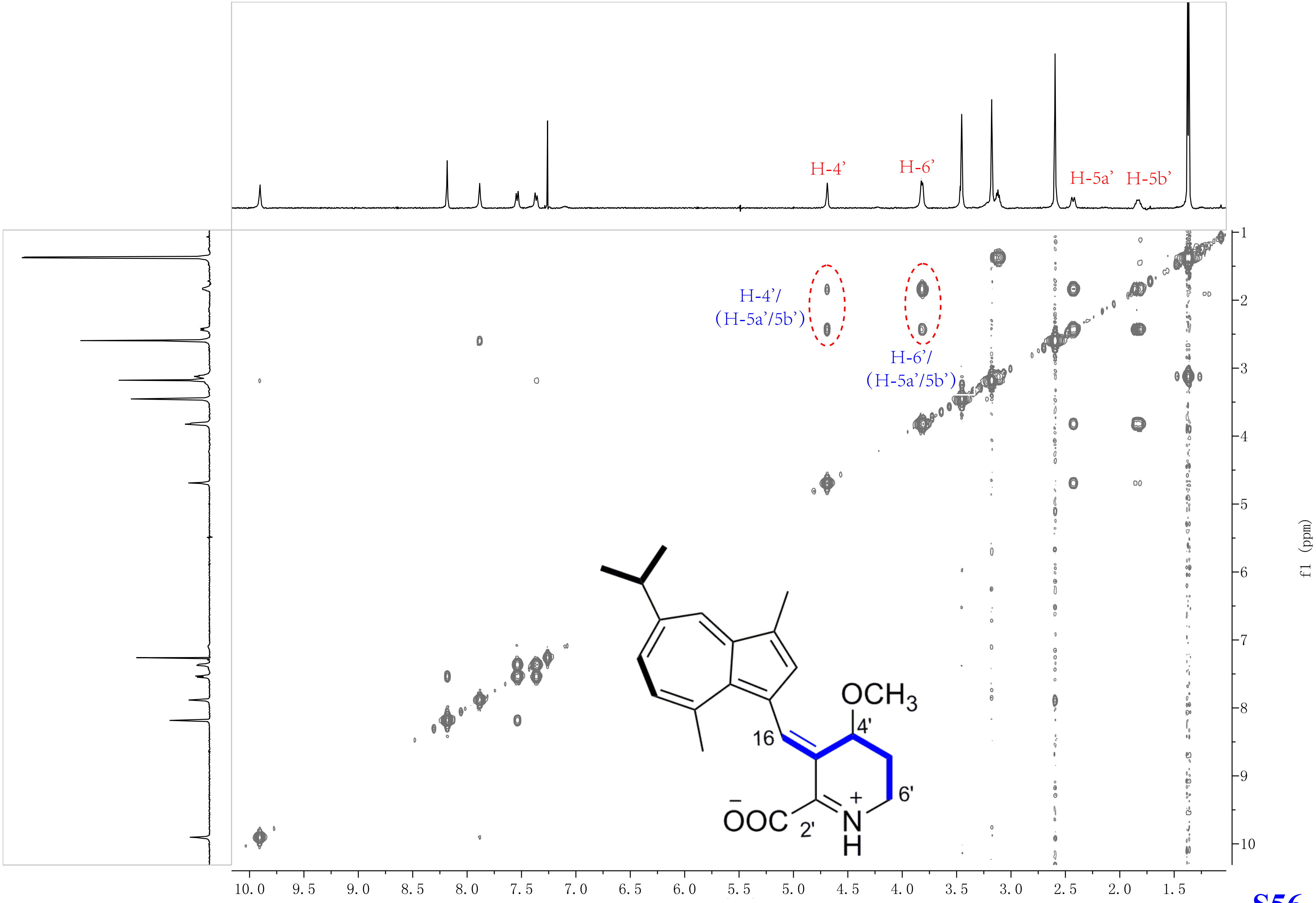

SS29 ^{1}H-^{1}H COSY spectrum of muriceidine C (3)

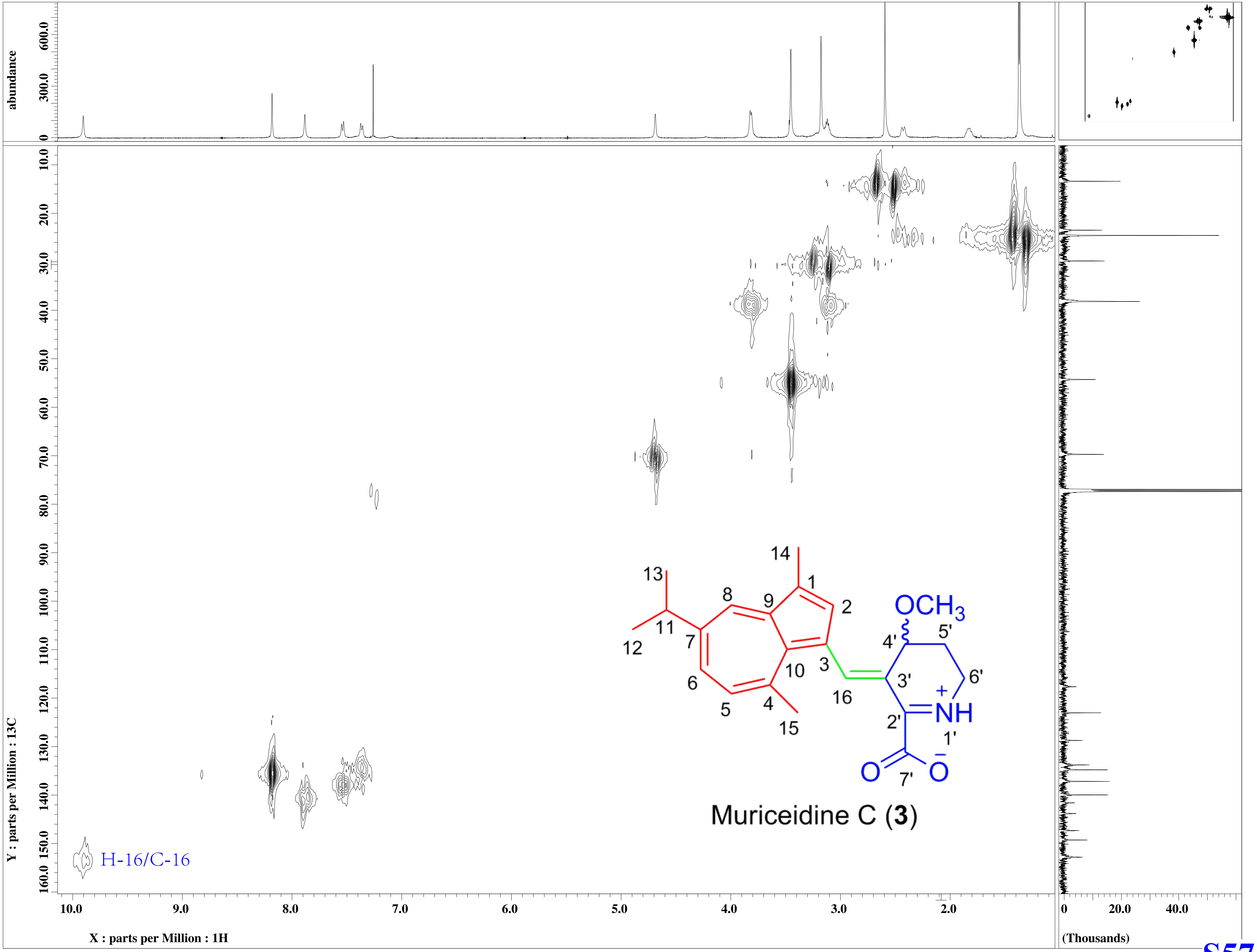

SS30 HMQC spectrum of muriceidine C (3)

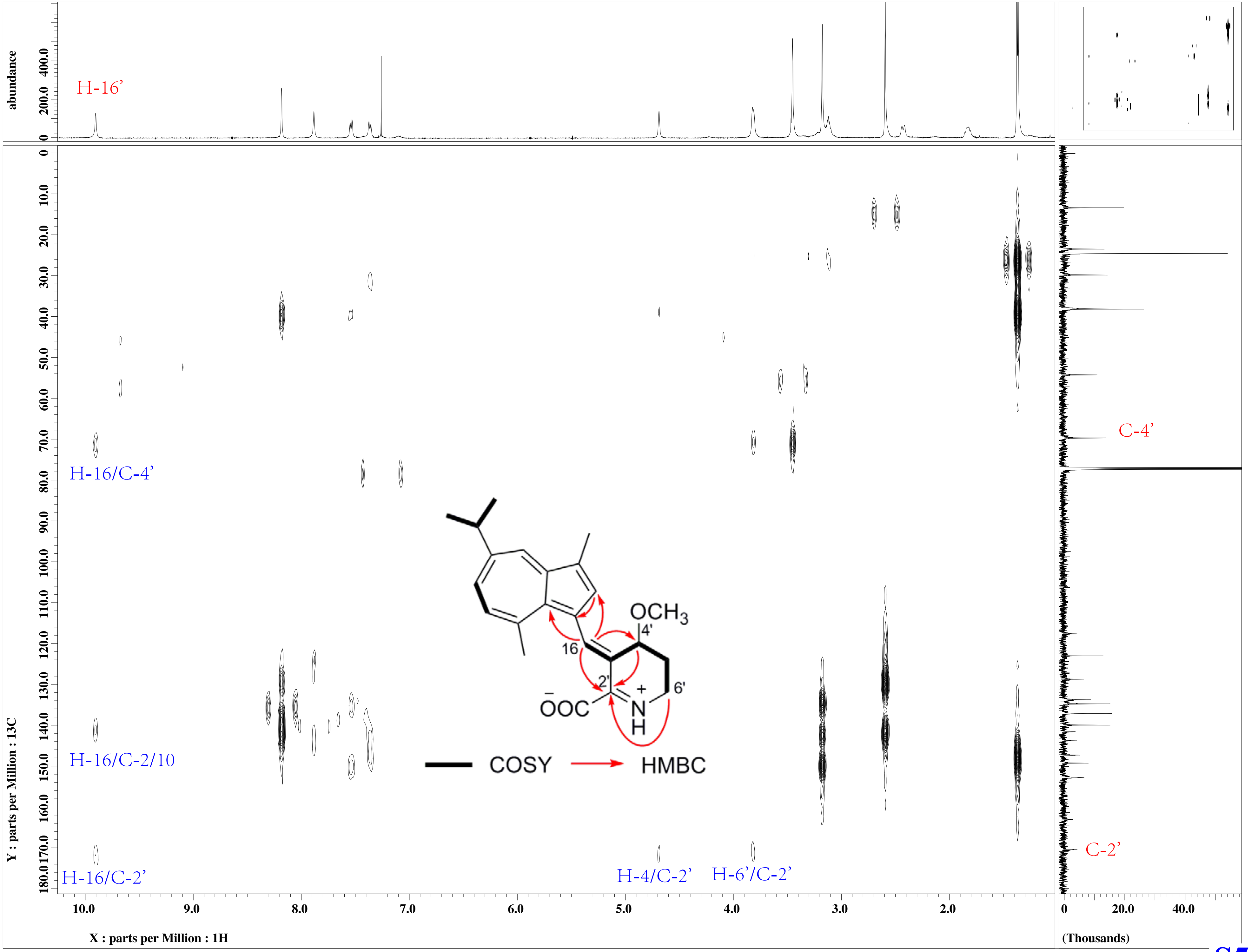

SS31 HMBC spectrum of muriceidine C (3)

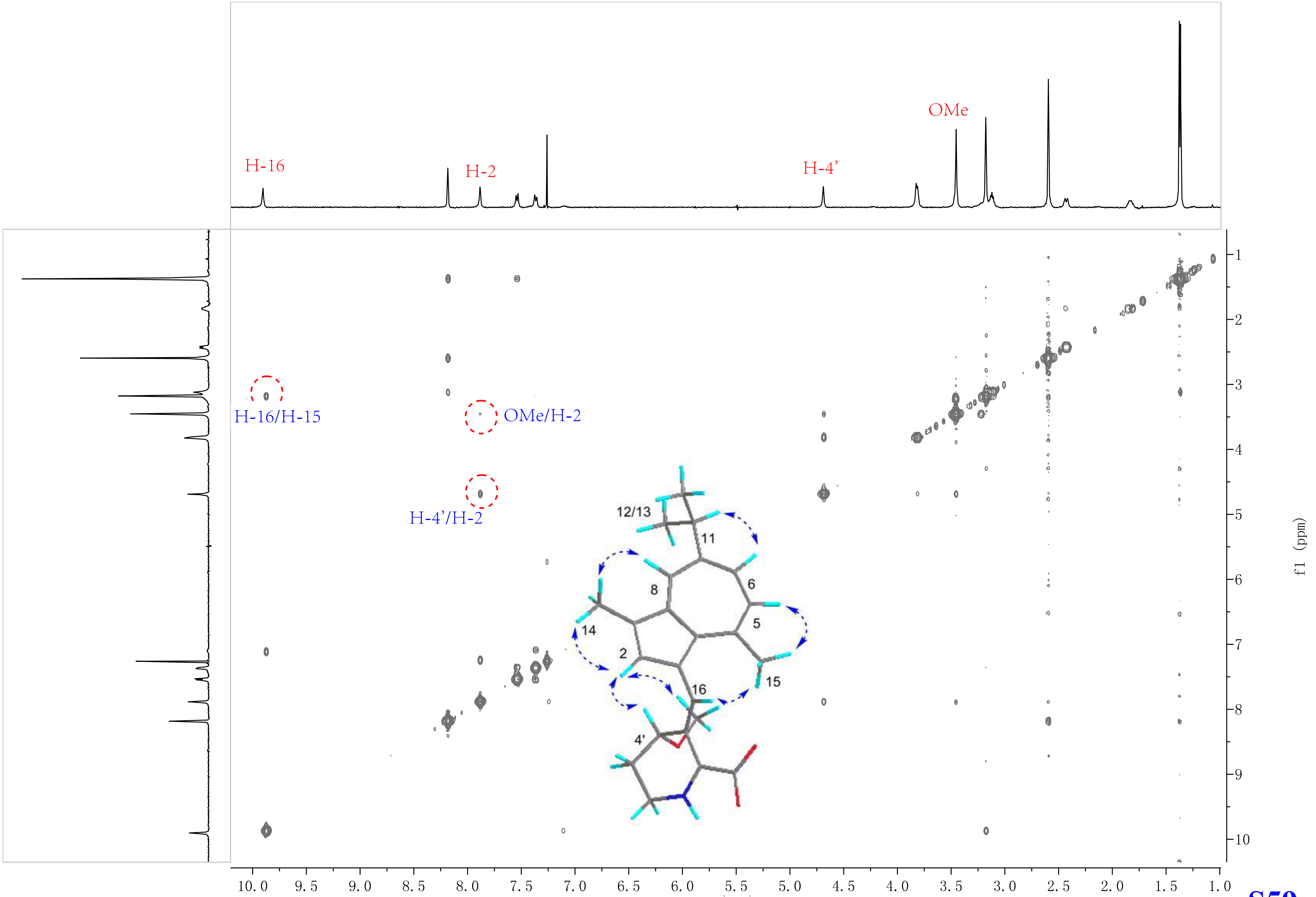

SS32 NOESY spectrum of muriceidine C (3)

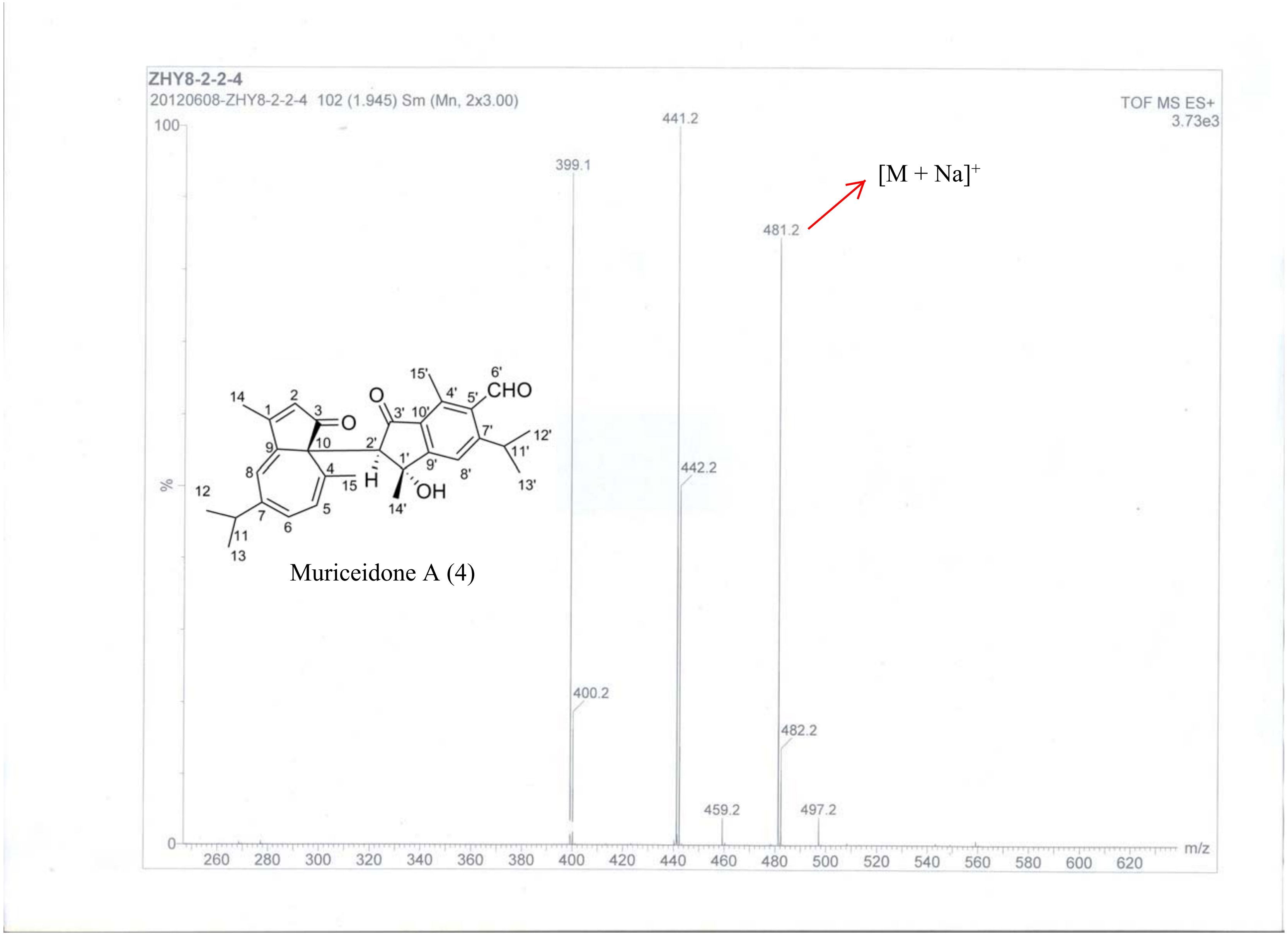

SS33 The positive ESIMS spectrum of muriceidone A (4)

20120613-ZHY-8-2-2-4_120612151233 #11-13 RT: 0.25-0.30 AV: 3 NL: 1.60E7
T: FTMS + p ESI Full ms [155.00-2000.00]

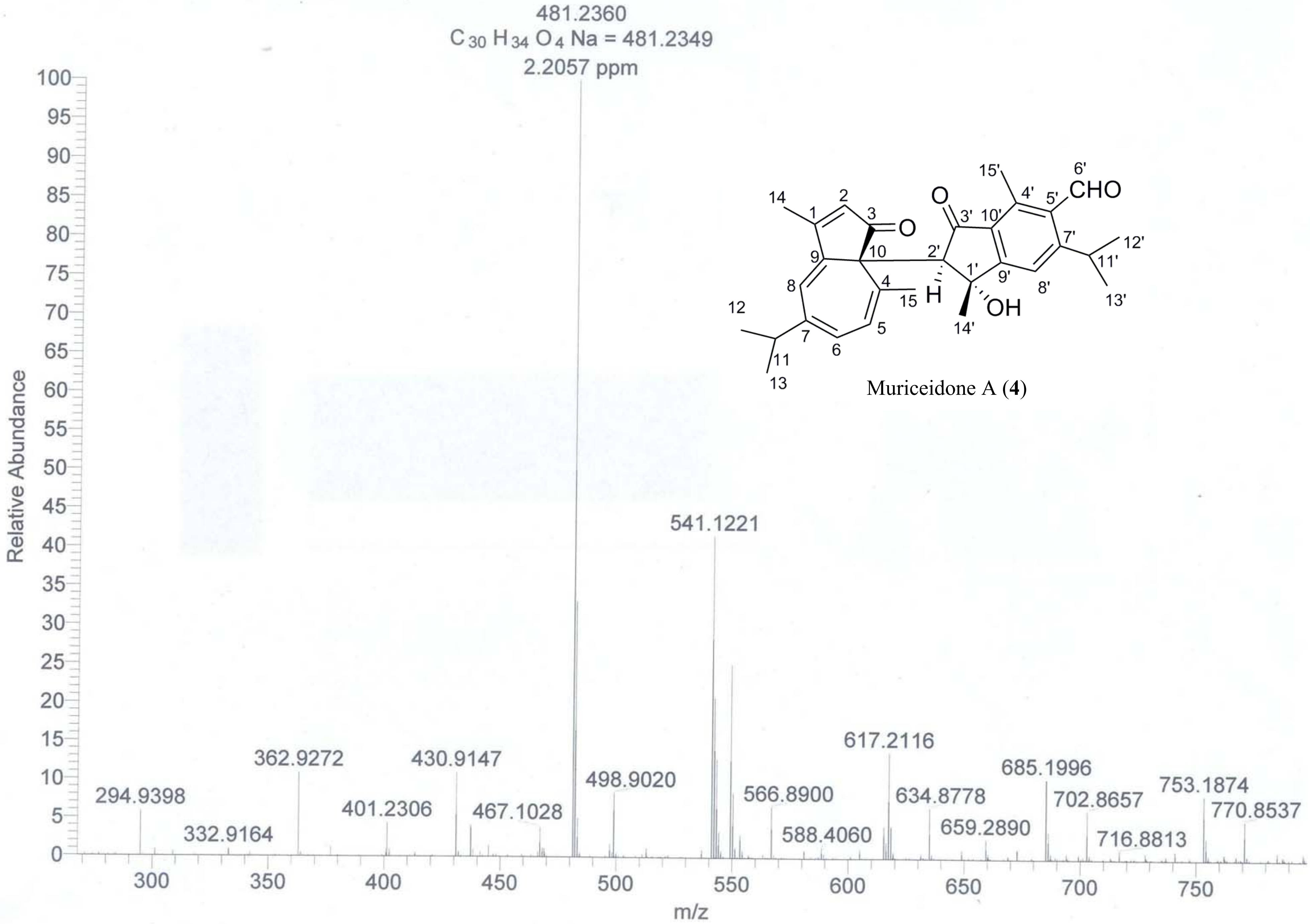

Muriceidone A (**4**)

SS34 The positive HRESIMS spectrum of muriceidone A (4)

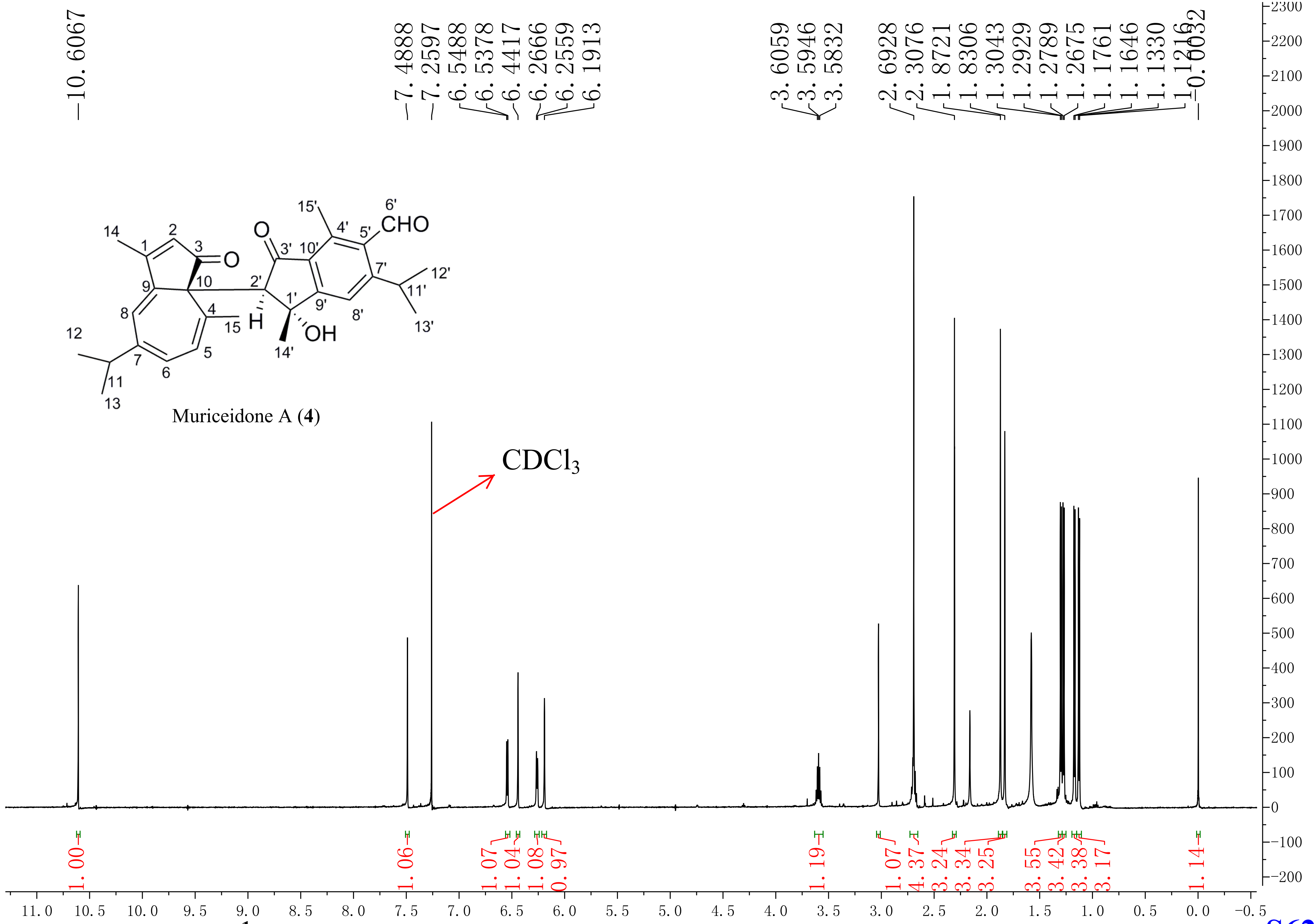

SS35 ^{1}H NMR (600 MHz, $CDCl_3$) spectrum of muriceidone A (**4**)

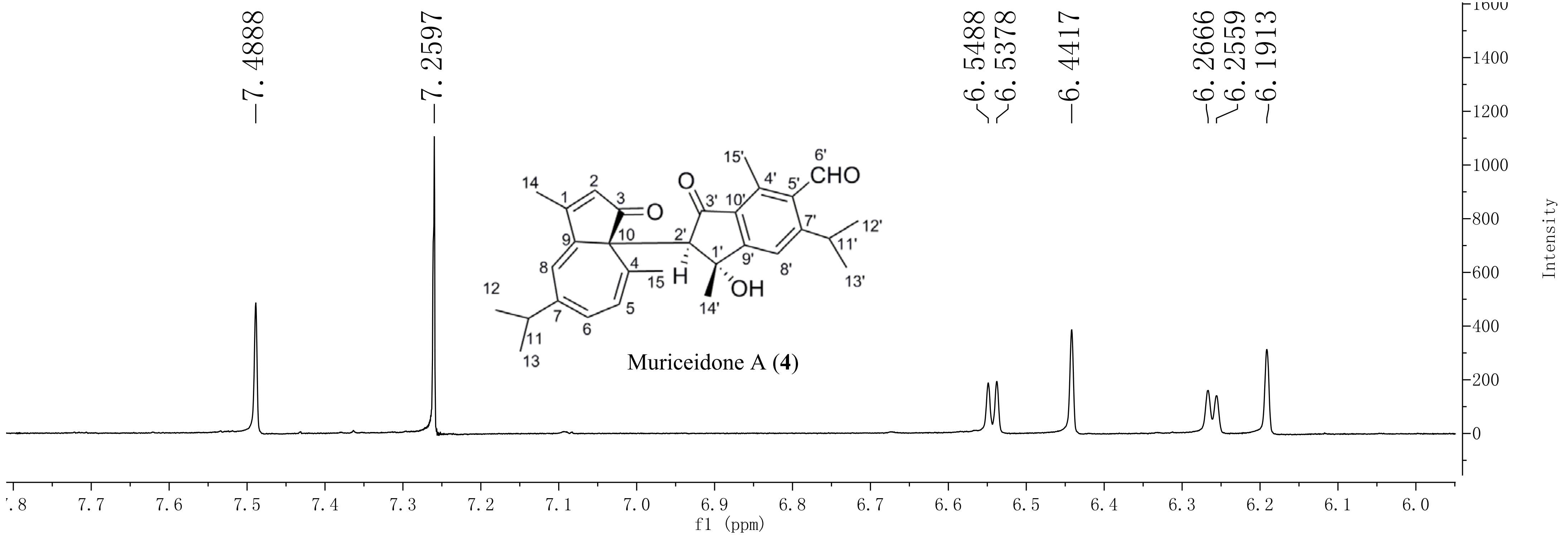

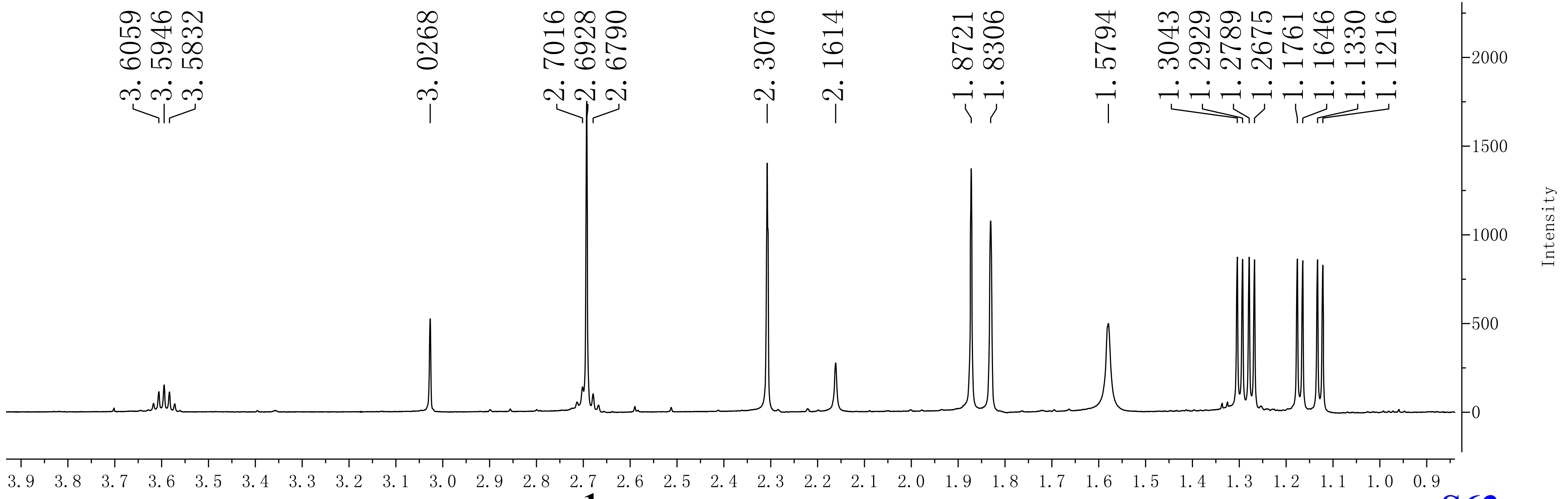

SS36 The amplificatory ^{1}H NMR spectrum of muriceidone A (4)

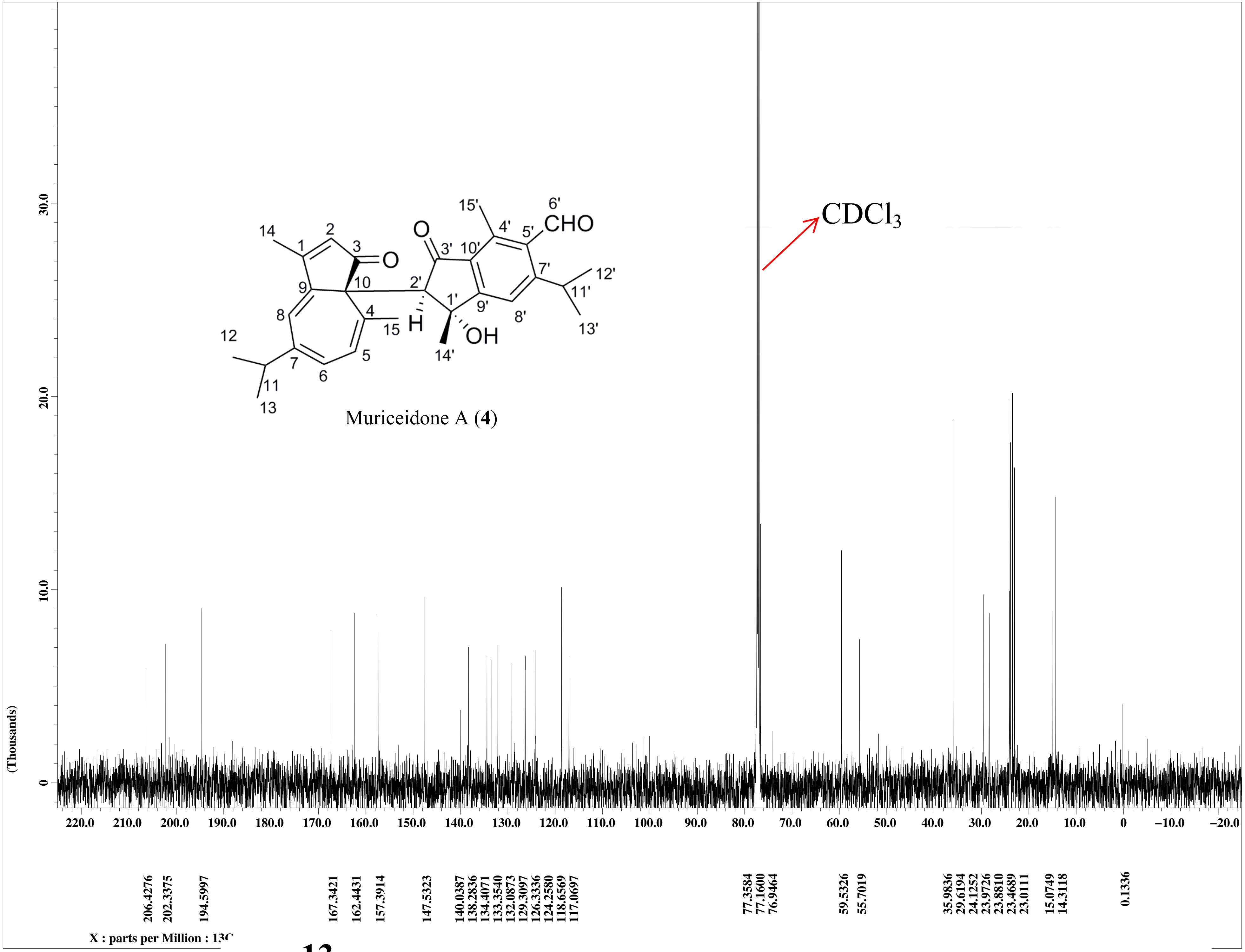

SS37 ^{13}C NMR (150 MHz, CDCl$_3$) spectrum of muriceidone A (4)

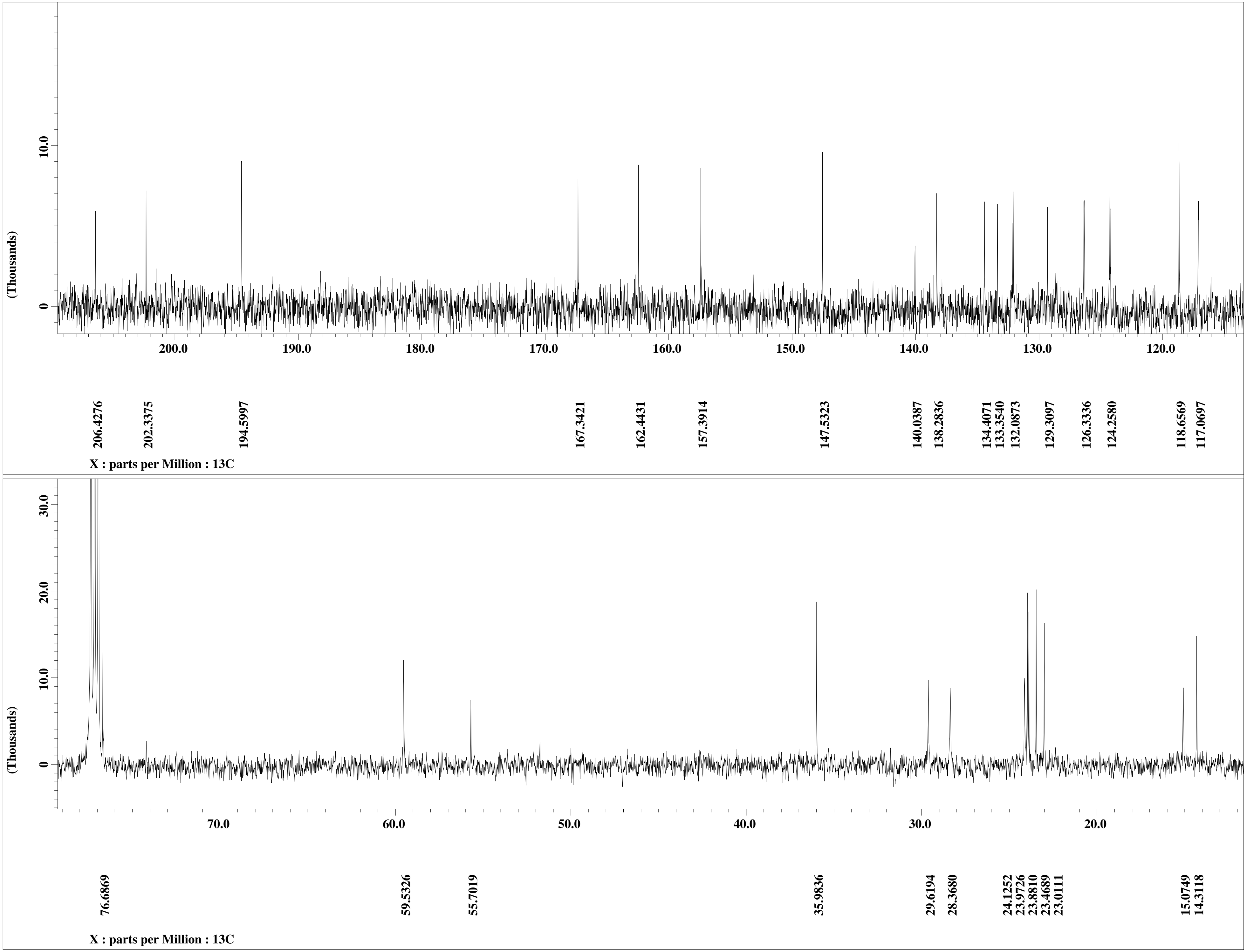

SS38 The amplificatory ^{13}C NMR spectrum of muricidone A (4)

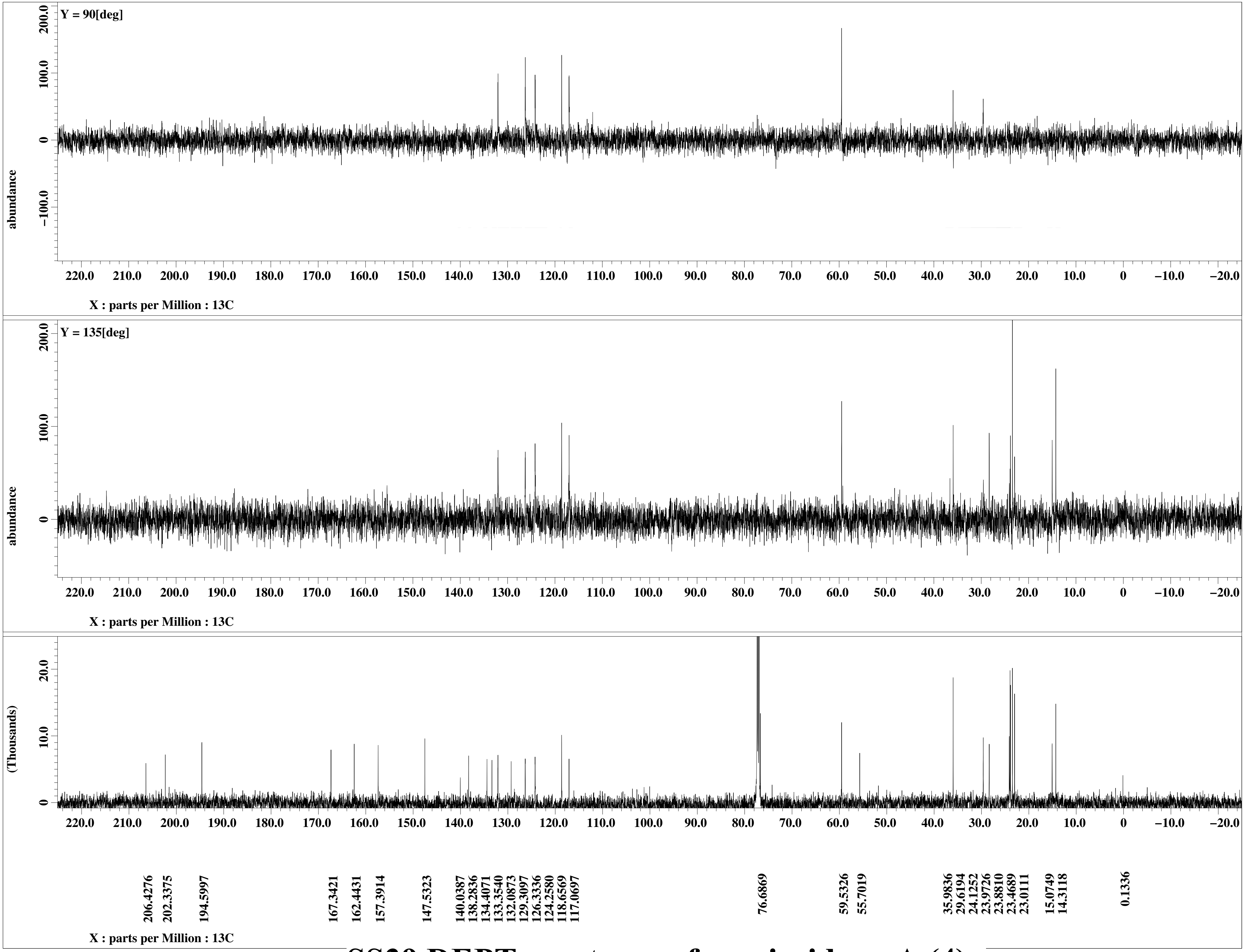

SS39 DEPT spectrum of muriceidone A (4)

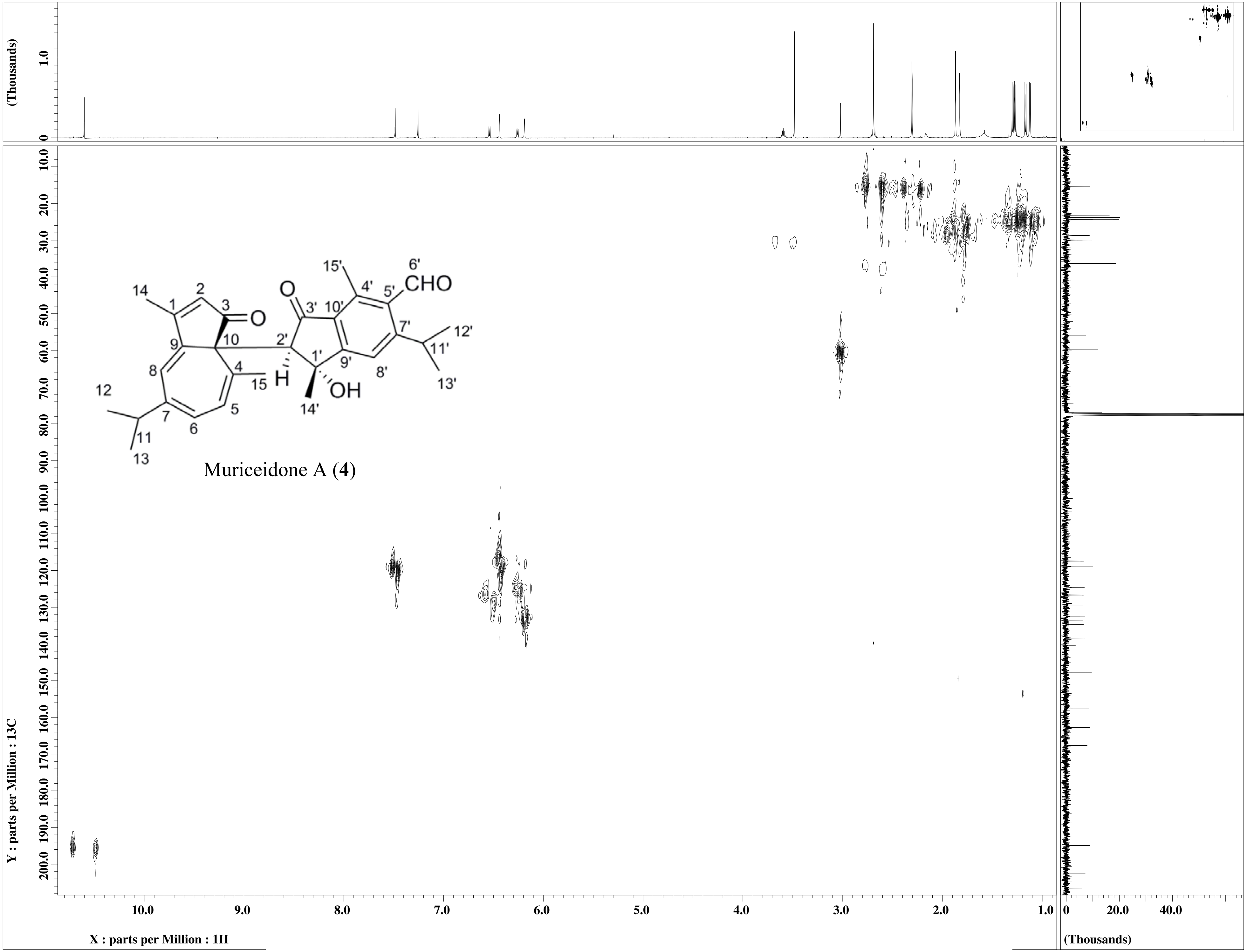

SS40 HMQC spectrum of muriceidone A (4)

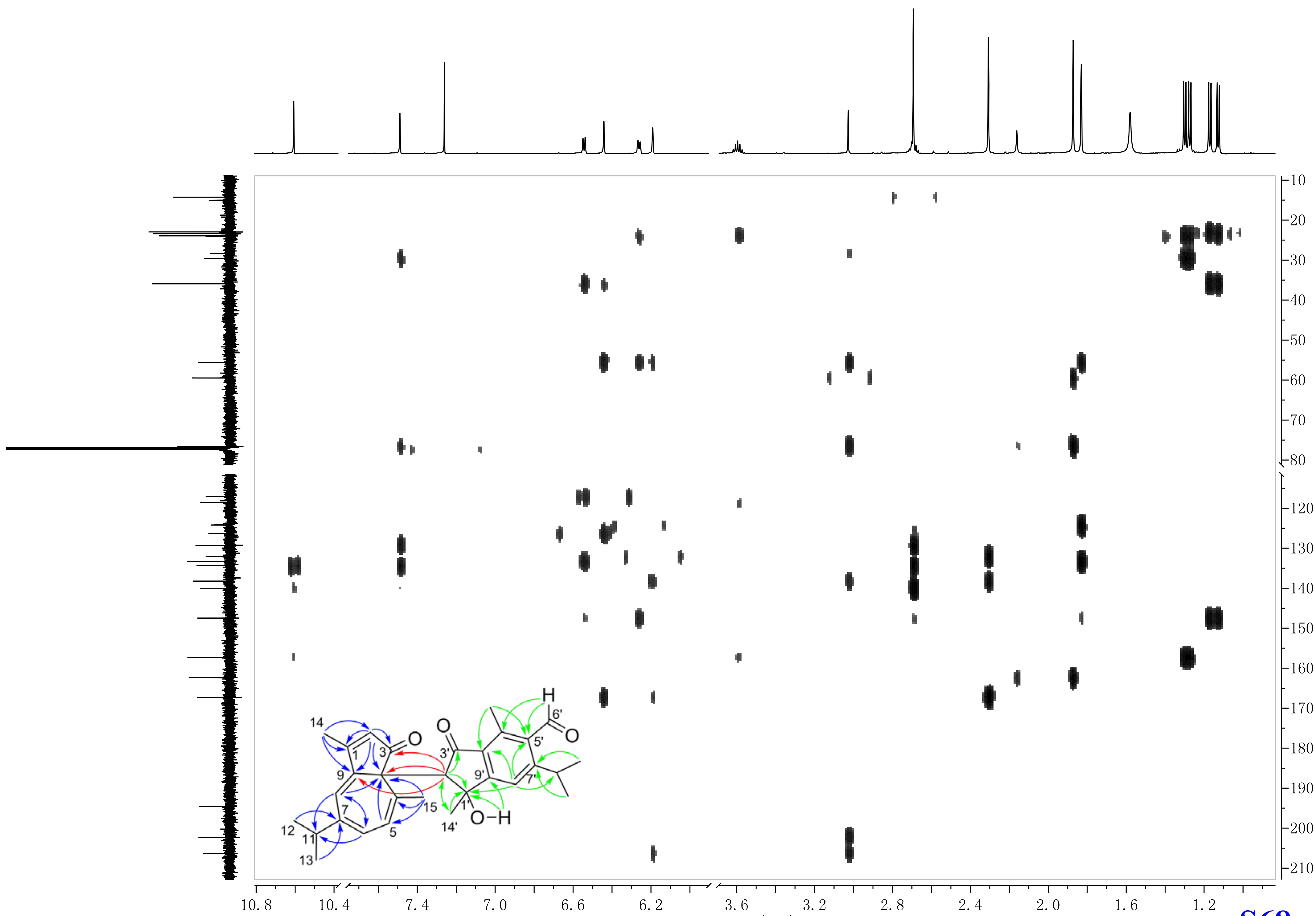

SS41 HMBC spectrum of muriceidone A (4)

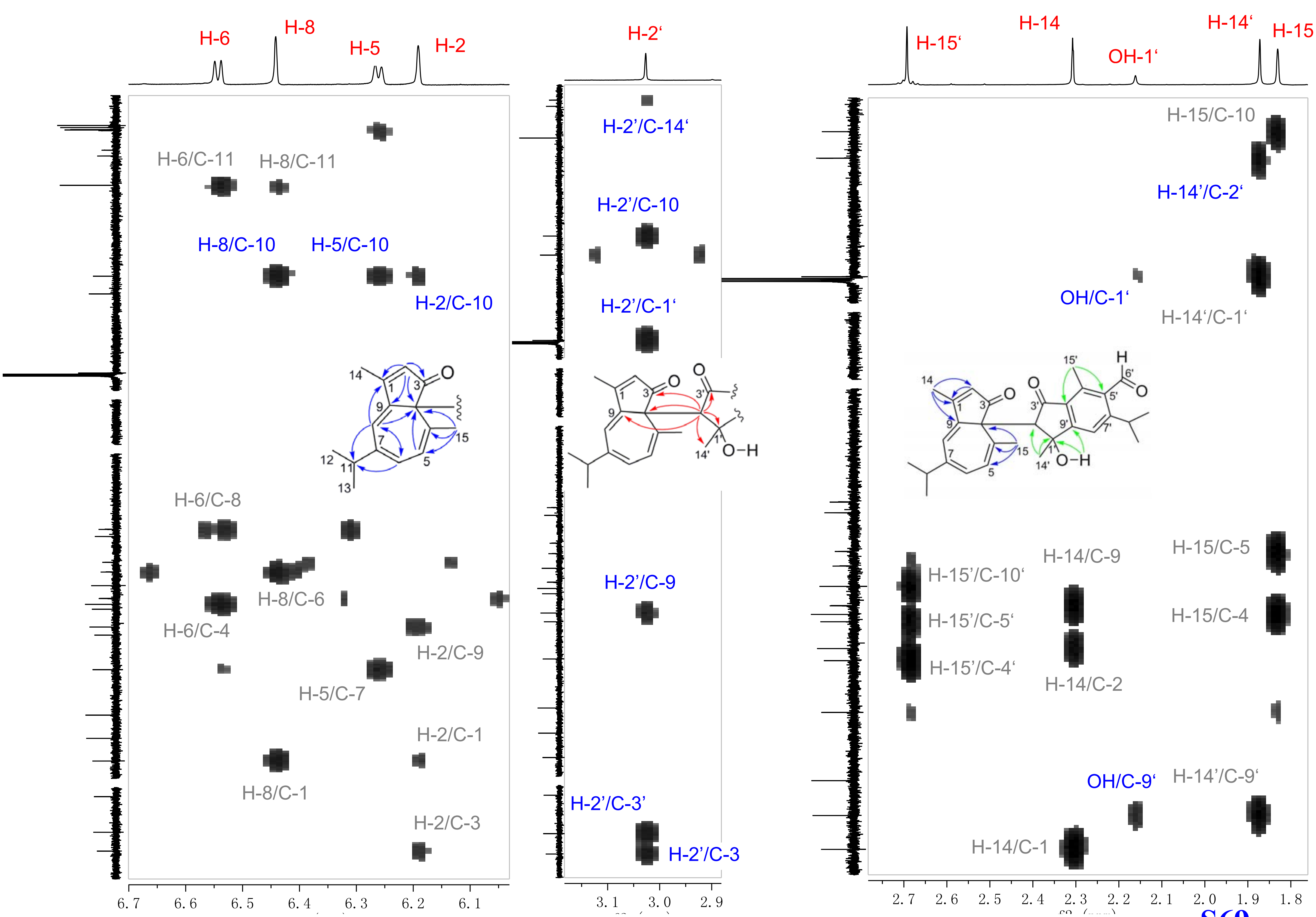

SS42 The amplificatory HMBC spectrum of muriceidone A (4)

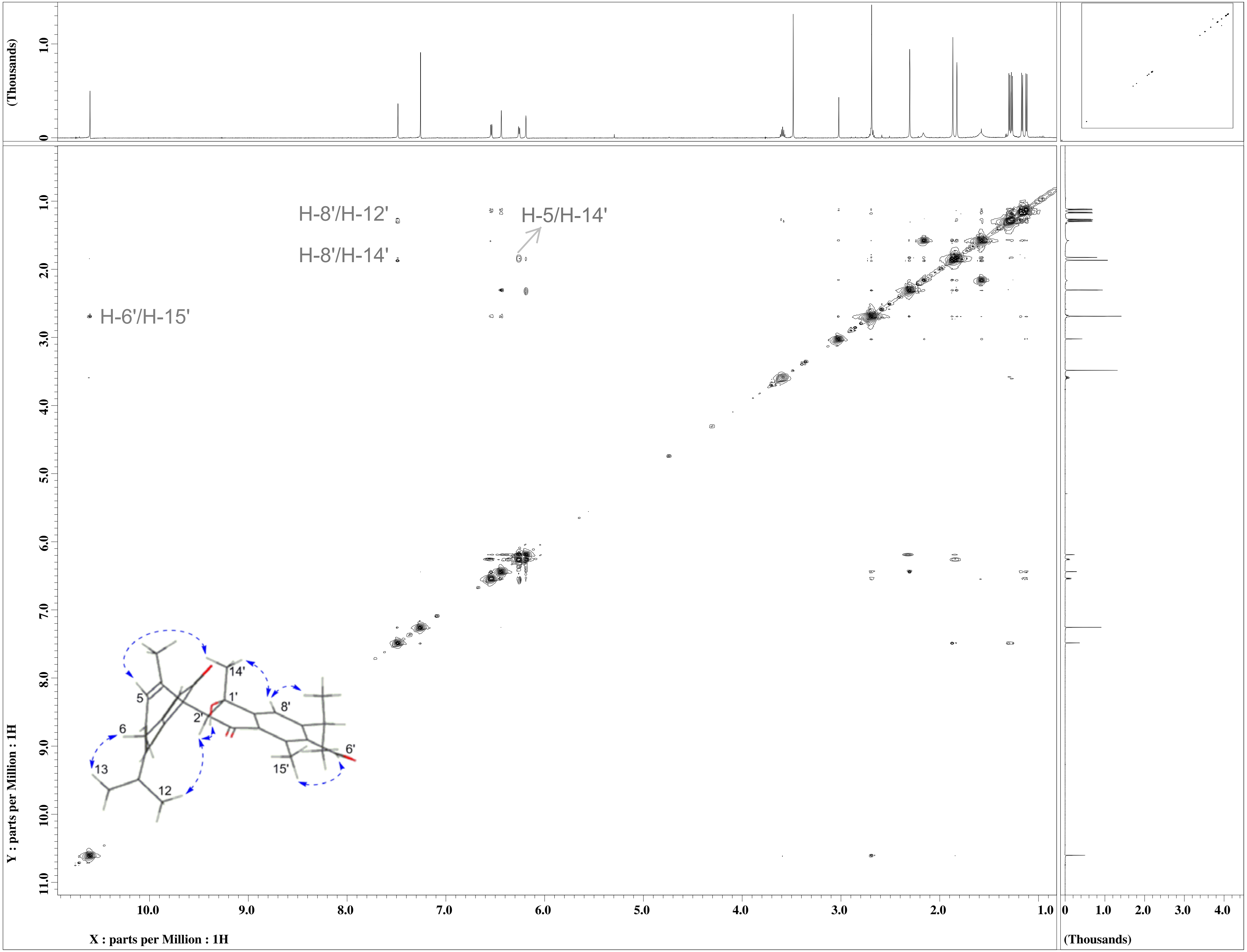

S43 NOESY spectrum of muriceidine C (4)

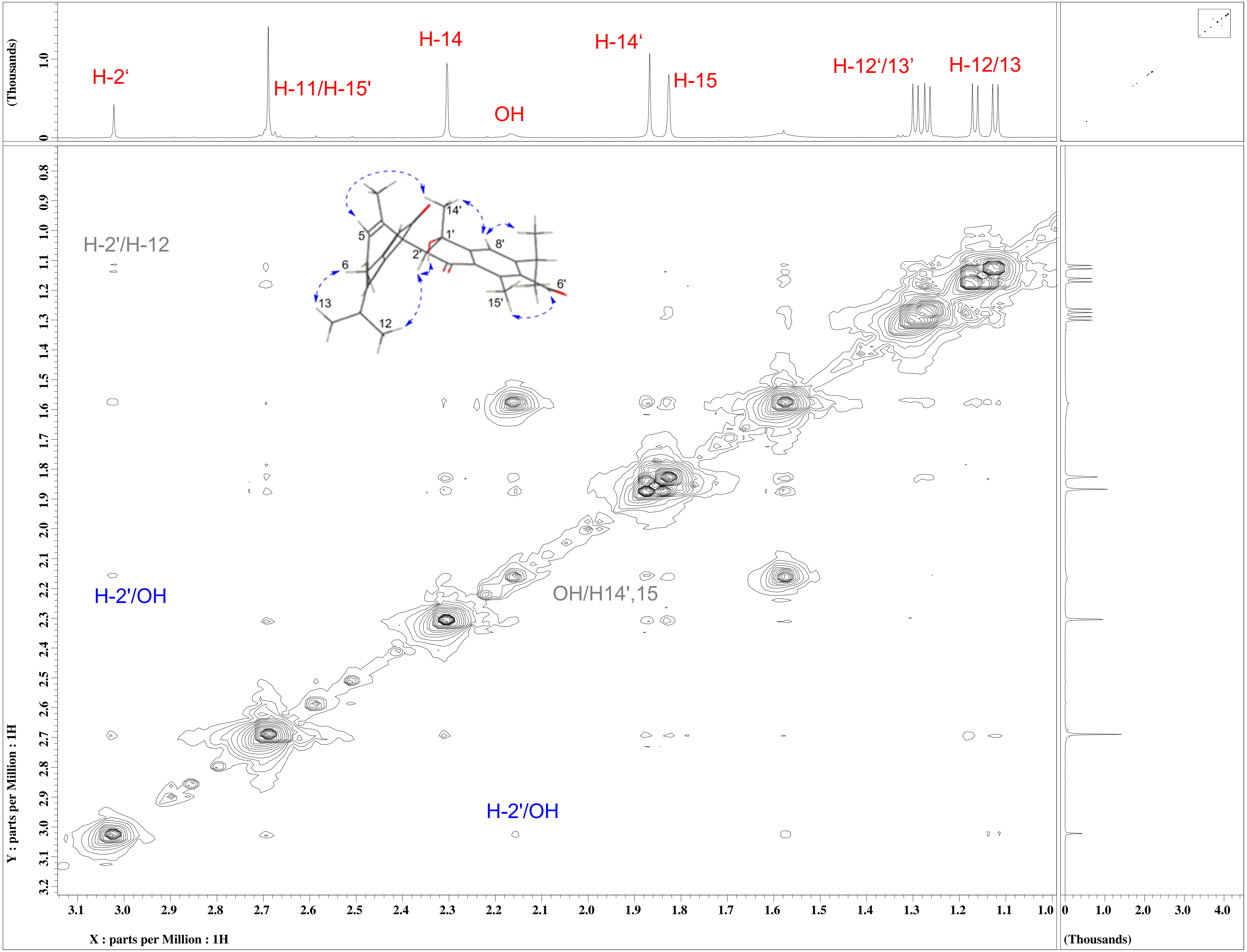

SS44 The amplificatory NOESY spectrum of muriceidine C (4)
